# An integrated group decision-making method for the evaluation of hypertension follow-up systems using interval-valued q-rung orthopair fuzzy sets

**DOI:** 10.1007/s40747-022-00953-w

**Published:** 2023-01-20

**Authors:** Benting Wan, Zhaopeng Hu, Harish Garg, Youyu Cheng, Mengjie Han

**Affiliations:** 1 Shenzhen Research Institute, Jiangxi University of Finance and Economics, Shenzhen, 518000 China; 2 School of Mathematics, Thapar Institute of Engineering and Technology (Deemed University), Patiala, Punjab 147004 India; 3 Department of Mathematics, Graphic Era Deemed to Be University, Dehradun, Uttarakhand 248002 India; 4 Applied Science Research Center, Applied Science Private University, Amman, 11931 Jordan; 5 School of Information and Engineering, Dalarna University, 79188 Falun, Sweden; 6 School of Software and IoT Engineering, Jiangxi University of Finance and Economics, Nanchang, 330013 China

**Keywords:** Interval-valued q-rung orthopair, WFA operator, LINMAP-ARAS decision-making method

## Abstract

It is imperative to comprehensively evaluate the function, cost, performance and other indices when purchasing a hypertension follow-up (HFU) system for community hospitals. To select the best software product from multiple alternatives, in this paper, we develop a novel integrated group decision-making (GDM) method for the quality evaluation of the system under the interval-valued q-rung orthopair fuzzy sets (IVq-ROFSs). The design of our evaluation indices is based on the characteristics of the HFU system, which in turn represents the evaluation requirements of typical software applications and reflects the particularity of the system. A similarity is extended to measure the IVq-ROFNs, and a new score function is devised for distinguishing IVq-ROFNs to figure out the best IVq-ROFN. The weighted fairly aggregation (WFA) operator is then extended to the interval-valued q-rung orthopair WFA weighted average operator (IVq-ROFWFAWA) for aggregating information. The attribute weights are derived using the LINMAP model based on the similarity of IVq-ROFNs. We design a new expert weight deriving strategy, which makes each alternative have its own expert weight, and use the ARAS method to select the best alternative based on these weights. With these actions, a GDM algorithm that integrates the similarity, score function, IVq-ROFWFAWA operator, attribute weights, expert weights and ARAS is proposed. The applicability of the proposed method is demonstrated through a case study. Its effectiveness and feasibility are verified by comparing it to other state-of-the-art methods and operators.

## Introduction

Hypertension is a chronic disease that requires long-term medication for patients. Some hypertensive patients can cause stroke and other comorbidities and can even lead to disability and death [65]. When about 1.28 billion people suffer from hypertension worldwide, failure to manage this disease effectively poses a heavy economic burden on both patients and the whole society [32, 61, 66]. Research results show that scientific intervention can control and reduce the risk of hypertension, and disease the development of associated cardiovascular and cerebrovascular in some cases prevent [32, 45, 47, 61, 65, 66]. However, a shortage of knowledgeable medical staff and poor patient self-management awareness continues to impede the further improvement of hypertension prevention levels [36]. To this end, researchers proposed the hypertension community management system that can provide better services for patients [27, 37, 54, 58, 82]. This system is safe, reliable and easy-to-use and it enables health facilities to treat hypertension more efficiently. It is no surprise that many community hospitals have purchased and started to use this system, thus raising the question of quality evaluation. Researchers are now calling for hypertension and software experts to come together to evaluate hypertension management systems [7, 8, 74]. For the software evaluation, the Institute of Electrical and Electronics Engineers (IEEE) has published different versions of the ISO/IEC standard [28], even though it is challenging to evaluate the application using these standards [4]. Researchers have proposed other evaluation methods and indices for different application scenarios according to actual requirements [70, 77]. For hospitals, the hypertension management system needs to meet performance, security, and scalability requirements while also meeting functional requirements [7, 8]. Its public-facing nature means it also needs to be easy-to-use [53, 64]. After the system is deployed and implemented, high-quality maintenance services are essential [26]. The evaluation indices of the hypertension community management system can be divided into product quality and post-services according to user aspects. Product quality indices are cost, function, performance, reliability, safety, scalability, integration, ease-of-use, and maintainability [11, 60] and post-service indices are supplier’s stability, follow-up services, and software deployment time [1, 6]. The indices required for the evaluation of the HFU system studied in this paper are described in “Evaluation indices of the HFU system”.

In the process of software evaluation decision-making, the flexibility of the information that experts can express is different depending on the fuzzy environment [46, 51, 56, 63]. As a software evaluation environment, researchers have proposed Fuzzy Sets [26], Intuitionistic Fuzzy Sets (IFSs) [60, 74], Type-2 Fuzzy Sets [7, 8], Pythagorean Fuzzy Sets (PFSs) [11], Interval-valued Intuitionistic Fuzzy Sets (IVIFSs) [7, 8], and Triangular Fuzzy Numbers (TFNs) [1]. A q-rung orthopair fuzzy set (q-ROFS) introduced by Yager [68] is also widely used in GDM [38]. The q-ROFSs are further generalized and applied to the fusion of various operators and decision methods. For example, Liu and Hussain proposed a new aggregation operator based on q-ROFSs [23, 39]. With non-cooperative game method introduced into q-ROFSs, Yang [71] theorized competitive strategy GDM problems based on a hybrid dynamic experts’ weight-determining model. The q-ROFSs were used to solve multi-attribute decision-making (MADM problems [15, 40], and they also have been applied to the group MADM (MAGDM) problem. To express the information of experts more freely, researchers developed IVq-ROFSs [31]. Yang [72] studied the GDM with incomplete interval-valued q-rung orthopair fuzzy (IVq-ROF) preference relations. New exponential operation laws and operators of IVq-ROFSs were developed by Garg [16, 17]. The IVq-ROFS FMEA was applied to improve the risk evaluation process of the tool changing manipulator [29]. Zhang [81] proposed an NA Operator based IVq-ROFSs, and Khan [34] used the combinative distance-based method to evaluate and select the strategy for a green supply chain under IVq-ROF environment. Moreover, the IVq-ROFSs were applied to sustainable smart waste management system evaluation using the multi-criteria decision-making (MCDM) model [57]. IVq-ROFSs have been applied in GDM [14, 16, 17, 72], but have not yet been applied to software management and assessment. In order to make it easier for software experts to evaluate the hypertension management system, IVq-ROFS is applied to the evaluation process in this paper.

Aggregation operators are used for fusing information provided by experts [3, 5, 13], such as weighted averaging (WA) [67], neutral aggregating (NA) [18], and power aggregating (PA) [69] operators. Some researchers have also extended their applications by considering the features of the IVq-ROFSs, for example, IVq-ROFWAMM and IVq-ROFAMM [13, 63]. Saha et al. [55] developed the q-rung orthopair fuzzy weighted fairly aggregation operator (q-ROFWFA) which exhibits neutral characteristics in the aggregating process. To minimize the impact of aggregating information from different software experts and enhance the quality of the evaluation information coming from the hypertension system, we extend the q-ROFWFA operator to interval-valued q-rung orthopair fuzzy weighted fairly aggregation operator (IVq-ROFWFAWA) in this paper.

Although aggregation operators can fuse information, they cannot handle complex problems. Many decision-making methods can be used, for example, the Linear Programming Technique for Multidimensional Analysis of Preference (LINMAP), Technique for Order Preference by Similarity to Ideal Solution (TOPSIS), multi-attributive border approximation area comparison (MABAC), MACBETH, VlseKriterijumska Optimizacija I Kompromisno Resenje (VIKOR), complex proportional assessment (COPRAS) and Interactive and Multicriteria Decision Making (TODIM) [2, 24, 42, 48, 59]. Among them, LINMAP [59] is a typical compromise model which can be used to derive weights [80] and widely applied in practical decision-making problems [9, 12, 35, 80]. Yu [73] integrated the LINMAP with prospect theory to find attribute weights. Mehrabadi and Boyaghchi [44] used the LINMAP for decision-making in geothermal multi-generational energy systems. Fetanat and Tayebi [12] employed LINMAP to design household water systems. However, according to the collected literature, there is no research on the application of the LINMAP model under IVq-ROFS environment. The Additive Ratio Assessment (ARAS), which was presented by Zavadskas and Turskis [79], selects the best alternative by employing a utility degree to reflect the difference between diverse alternatives and the ideal one. ARAS eliminates the influence of unlike measurement units. For this reason, it has received considerable attention from researchers. Heidary et al. [22] used the ARAS to rank high-performance human resource practices. Gül [21] employed ARAS to deal with problems related to the selection of covid-19 experiments. Jovčić et al. [30] used it to make decisions about goods distribution. This paper extends the ARAS method to IVq-ROFS and further devises a new GDM method. However, the result of this integrated GDM method is still IVq-ROFNs. To clarify the aggregated values, it has been necessary to use, like many other researchers have done [20, 38, 62, 67] the score function. These researchers have put forward their own score functions for IVIFNs. Although these score function are effective for solving MADM problems, there still are some deficiencies. To overcome their weaknesses, this article investigates and applies a new score function so that the different software suppliers can be distinguished from each other and allow the best software supplier to be identified.

Community hospitals want an objective means for evaluating their software system and one that is cost-effective. They also want the evaluation results so they can be mapped directly onto the judgment matrix provided by the experts. These desires transform the software evaluation decision into a MAGDM problem with unknown expert weights and attribute weights [52]. In order to derive the attribute weights and expert weights, the LINMAP can be fused by similarity [33]. While the LINMAP is a mature method for solving attribute weights [59], it has not yet been used to determine expert weights. Currently, Yue [75, 76] suggested an expert weight based on similarity and projection. The two methods cannot distinguish the influence of the external environment because experts cannot always maintain their objectivity and fairness. For this reason, we will study the weights of the experts by examining the similarity of the distinct alternatives, and we will derive the experts’ weight matrices that will be used to gather information on the software suppliers from the different experts.

The evaluation method proposed in this article takes the software evaluation information provided by the experts and then integrates the IVq-ROFWFAWA operator, the ARAS method, a novel score function and the similarity under IVq-ROFS to capture the optimal software supplier. This method will improve decision-making efficiency and save evaluation costs. System purchasers will not need to know the expert weights or attribute weights. Our contributions, therefore, are as follows.

1. A novel score function is defined to rank the IVq-ROFNs.
2. The IVq-ROFWFA and IVq-ROFWFAWA operators are extended based on the q-ROFWFA operator.
3. Attribute weights are derived from LINMAP based on the similarity of IVq-ROFNs.
4. Expert weights of different alternatives are proposed. To reduce the decision results affected by experts’ judgments and the external environment, we suggest that different alternatives should have different expert weights.
5. A new integrated MAGDM method has been developed based on the ARAS in this paper. This method combines an IVq-ROFWFAWA operator, LINMAP and ARAS to address decision-making problems.
6. The community HFU management system was evaluated by our MAGDM method. The results confirm that the MAGDM method has strong adaptability and is compatible with existing algorithms. Comparative analysis results confirm that the proposed MAGDM method is effective.

The remainder of this paper is arranged as follows. The next section introduces the preliminaries. “Proposed score function and operators” extends the WFA operator to the IVq-ROFNs. “Integrated group decision method” develops an integrated MAGDM method based on ARAS. “Evaluation and analysis of the HFU system” presents the evaluation process and its analysis of the HFU management system. “Conclusion” offers a conclusion and some suggestions for the direction of future research.

## Preliminaries

### IVq-ROFS

#### Definition 1

 [63] Let *X* be the domain of discourse. An IVq-ROFS in *X* is indicated by

1 $$A=\left\{\left(x,{u}_{A}\left(x\right),{v}_{A}\left(x\right)\right)\left|x\in X\right.\right\},$$

where the membership and non-membership functions are the mapping range of values to meet ${u}_{A}\left(x\right)=\left[{u}_{A}^{-}\left(x\right),{u}_{A}^{+}\left(x\right)\right]\subseteq \left[{0,1}\right]$,${v}_{A}\left(x\right)=\left[{v}_{A}^{-}\left(x\right),{v}_{A}^{+}\left(x\right)\right]\subseteq \left[{0,1}\right]$,$0\le ({u}_{A}^{+}\left(x\right){)}^{q}+({v}_{A}^{+}\left(x\right){)}^{q}\le 1,\left(q\ge 1\right)$. The hesitation degree of $A$ is shown in Eq. (2):

2 $${\pi }_{A}\left(x\right)=\left[{\pi }_{A}^{-}\left(x\right),{\pi }_{A}^{+}\left(x\right)\right]=\left[\sqrt[q]{1-({u}_{A}^{+}\left(x\right){)}^{q}-({v}_{A}^{+}\left(x\right){)}^{q}}, \sqrt[q]{1-({u}_{A}^{-}\left(x\right){)}^{q}-({v}_{A}^{-}\left(x\right){)}^{q}}\right].$$

#### Definition 2

 [63]. Let $a=\left(\left[{u}_{a}^{-},{u}_{a}^{+}\right],\left[{v}_{a}^{-},{v}_{a}^{+}\right]\right)$, ${a}_{1}=\left(\left[{u}_{{a}_{1}}^{-},{u}_{{a}_{1}}^{+}\right],\left[{v}_{{a}_{1}}^{-},{v}_{{a}_{1}}^{+}\right]\right)$ and ${a}_{2}=\left(\left[{u}_{{a}_{2}}^{-},{u}_{{a}_{2}}^{+}\right],\left[{v}_{{a}_{2}}^{-},{v}_{{a}_{2}}^{+}\right]\right)$ be the three IVq-ROFNs with $q\ge 1$, and $\lambda >0$. Some operations between $a$, ${a}_{1}$ and ${a}_{2}$ can be defined as follows:

3 $$1. \quad  {a}_{1}\oplus {a}_{2}=\left(\left[\sqrt[q]{({u}_{{a}_{1}}^{-}{)}^{q}+({u}_{{a}_{2}}^{-}{)}^{q}-({u}_{{a}_{1}}^{-}{)}^{q}({u}_{{a}_{2}}^{-}{)}^{q}},\sqrt[q]{({u}_{{a}_{1}}^{+}{)}^{q}+({u}_{{a}_{2}}^{+}{)}^{q}-({u}_{{a}_{1}}^{+}{)}^{q}({u}_{{a}_{2}}^{+}{)}^{q}}\right],\left[{v}_{{a}_{1}}^{-}{v}_{{a}_{2}}^{-},{v}_{{a}_{1}}^{+}{v}_{{a}_{2}}^{+}\right]\right)$$

4 $$2. \quad  {a}_{1}\otimes {a}_{2}=\left(\left[{u}_{{a}_{1}}^{-}{u}_{{a}_{2}}^{-},{u}_{{a}_{1}}^{+}{u}_{{a}_{2}}^{+}\right],\left[\sqrt[q]{({v}_{{a}_{1}}^{-}{)}^{q}+({v}_{{a}_{2}}^{-}{)}^{q}-({v}_{{a}_{1}}^{-}{)}^{q}({v}_{{a}_{2}}^{-}{)}^{q}},\sqrt[q]{({v}_{{a}_{1}}^{+}{)}^{q}+({v}_{{a}_{2}}^{+}{)}^{q}-({v}_{{a}_{1}}^{+}{)}^{q}({v}_{{a}_{2}}^{+}{)}^{q}}\right]\right)$$

5 $$3. \quad {a}^{\lambda }=\left(\left[({u}_{a}^{-}{)}^{\lambda },({u}_{a}^{+}{)}^{\lambda }\right],\left[\sqrt[q]{1-(1-({v}_{a}^{-}{)}^{q}{)}^{\lambda }},\sqrt[q]{1-(1-({v}_{a}^{+}{)}^{q}{)}^{\lambda }}\right]\right).$$

### q-ROFWFA operator

The WFA operator can increase the density of the information that experts can obtain by evaluating the neutrality and fairness of the data during the decision-making process.

#### Definition 3

 [55] Given any two q-ROFNs ${a}_{1}$ and ${a}_{2}$, ${a}_{1}{\otimes }_{F}{a}_{2}$ and ${\lambda }_{F}{a}_{1}$ represent, respectively, the multiplication and scalar multiplication operation rules of the q-ROFWFA operator of two q-ROFNs, as shown in Eqs. (6) and (7):

6 $${a}_{1}{\otimes }_{F}{a}_{2}\,\left(\begin{array}{l}{\left(\left({\frac{{u}_{{a}_{1}}^{q}{u}_{{a}_{2}}^{q}}{{u}_{{a}_{1}}^{q}{u}_{{a}_{2}}^{q}+{v}_{{a}_{1}}^{q}{v}_{{a}_{2}}^{q}}}\right)\times (1-(1-{u}_{{a}_{1}}^{q}-{v}_{{a}_{1}}^{q})(1-{u}_{{a}_{2}}^{q}-{v}_{{a}_{2}}^{q}))\right)}^{\frac{1}{q}},\\ {\left(\left({\frac{{v}_{{a}_{1}}^{q}{v}_{{a}_{2}}^{q}}{{u}_{{a}_{1}}^{q}{u}_{{a}_{2}}^{q}+{v}_{{a}_{1}}^{q}{v}_{{a}_{2}}^{q}}}\right)\times (1-(1-{u}_{{a}_{1}}^{q}-{v}_{{a}_{1}}^{q})(1-{u}_{{a}_{2}}^{q}-{v}_{{a}_{2}}^{q}))\right)}^{\frac{1}{q}}\end{array}\right),$$

7 $${\lambda }_{F}{a}_{1}=\left({\left(\left({\frac{{u}_{{a}_{1}}^{q\lambda }}{{u}_{{a}_{1}}^{q\lambda }+{v}_{{a}_{1}}^{q\lambda }}}\right)\times (1-(1-{u}_{{a}_{1}}^{q}-{v}_{{a}_{1}}^{q}{)}^{\lambda })\right)}^{\frac{1}{q}},{\left(\left({\frac{{v}_{{a}_{1}}^{q\lambda }}{{u}_{{a}_{1}}^{q\lambda }+{v}_{{a}_{1}}^{q\lambda }}}\right)\times (1-(1-{u}_{{a}_{1}}^{q}-{v}_{{a}_{1}}^{q}{)}^{\lambda })\right)}^{\frac{1}{q}}\right).$$

The q-ROFWFA operator can, by evaluating the data, scientifically and comprehensively consider the preferences of different experts and by so doing obtain rich and diversified information. It is used for aggregating information during the process of MAGDM. The q-ROFWFA operator is stated in Definition 4.

#### Definition 4

[55] Let ${\alpha }_{i}=\left({u}_{{\alpha }_{i}}, {v}_{{\alpha }_{i}}\right),\left(i={1,2},\dots ,n\right)$ be a set of q-ROFNs. The q-ROFWFA operator is

8 $$\begin{array}{cc}q-\mathrm{ROFWFA}\left({\alpha }_{1},{\alpha }_{2},\dots ,{\alpha }_{n}\right)&= \left(\begin{array}{c}{\left(\frac{{\prod }_{i=1}^{n}{\left({u}_{{\alpha }_{i}}^{q}\right)}^{{w}_{i}}}{{\prod }_{i=1}^{n}{\left({u}_{{\alpha }_{i}}^{q}\right)}^{{w}_{i}}+{\prod }_{i=1}^{n}{\left({v}_{{\alpha }_{i}}^{q}\right)}^{{w}_{i}}}\times \left(1-{\prod }_{i=1}^{n}{\left(1-{u}_{{\alpha }_{i}}^{q}-{v}_{{\alpha }_{i}}^{q}\right)}^{{w}_{i}}\right)\right)}^{\frac{1}{q}},\\ {\left(\frac{{\prod }_{i=1}^{n}{\left({v}_{{\alpha }_{i}}^{q}\right)}^{{w}_{i}}}{{\prod }_{i=1}^{n}{\left({u}_{{\alpha }_{i}}^{q}\right)}^{{w}_{i}}+{\prod }_{i=1}^{n}{\left({v}_{{\alpha }_{i}}^{q}\right)}^{{w}_{i}}}\times \left(1-{\prod }_{i=1}^{n}{\left(1-{u}_{{\alpha }_{i}}^{q}-{v}_{{\alpha }_{i}}^{q}\right)}^{{w}_{i}}\right)\right)}^{\frac{1}{q}}\end{array}\right)\end{array}.$$

In Eq. (8), ${w}_{i}$ is the weight of ${\alpha }_{i}\left(i={12,3},\dots ,n\right)$, and must satisfy ${w}_{i}\ge 0$, ${\sum }_{i=1}^{n}{w}_{i}=1$.

### Interval-valued q-rung orthopair fuzzy similarity

The similarity is used to measure the degree of similarity between two fuzzy subsets. For any two fuzzy numbers in a fuzzy set, similarity can be used to reflect the difference and to distinguish their relationship. Inspired by the similarity suggested in a previous study [52], this paper proposes the similarity of IVq-ROFNs that is as shown in Definition 6.

#### Definition 6

Let ${a}_{1}=\left(\left[{u}_{{a}_{1}}^{-},{u}_{{a}_{1}}^{+}\right],\left[{v}_{{a}_{1}}^{-},{v}_{{a}_{1}}^{+}\right]\right)$ and ${a}_{2}=\left(\left[{u}_{{a}_{2}}^{-},{u}_{{a}_{2}}^{+}\right],\left[{v}_{{a}_{2}}^{-},{v}_{{a}_{2}}^{+}\right]\right)$ be two IVq-ROFNs, if ${u}_{{a}_{1}}^{-},{u}_{{a}_{1}}^{+},{v}_{{a}_{1}}^{-},{v}_{{a}_{1}}^{+}$, ${u}_{{a}_{2}}^{-},{u}_{{a}_{2}}^{+},{v}_{{a}_{2}}^{-},{v}_{{a}_{2}}^{+}$ are all 0, the similarity will be 1. When ${u}_{{a}_{1}}^{-},{u}_{{a}_{1}}^{+},{v}_{{a}_{1}}^{-},{v}_{{a}_{1}}^{+}$, ${u}_{{a}_{2}}^{-},{u}_{{a}_{2}}^{+},{v}_{{a}_{2}}^{-},{v}_{{a}_{2}}^{+}$ are not 0, the similarity measure between ${a}_{1}$ and ${a}_{2}$ is introduced in Eq. (9):

9 $$S\left({a}_{1},{a}_{2}\right)={\frac{\left({\left({u}_{{a}_{1}}^{-}\right)}^{q}\wedge {\left({u}_{{a}_{2}}^{-}\right)}^{q}\right)+\left({\left({v}_{{a}_{1}}^{-}\right)}^{q}\wedge {\left({v}_{{a}_{2}}^{-}\right)}^{q}\right)+\left({\left({u}_{{a}_{1}}^{+}\right)}^{q}\wedge {\left({u}_{{a}_{2}}^{+}\right)}^{q}\right)+\left({\left({v}_{{a}_{1}}^{+}\right)}^{q}\wedge {\left({v}_{{a}_{2}}^{+}\right)}^{q}\right)}{\left({\left({u}_{{a}_{1}}^{-}\right)}^{q}\vee {\left({u}_{{a}_{2}}^{-}\right)}^{q}\right)+\left({\left({v}_{{a}_{1}}^{-}\right)}^{q}\vee {\left({v}_{{a}_{2}}^{-}\right)}^{q}\right)+\left({\left({u}_{{a}_{1}}^{+}\right)}^{q}\vee {\left({u}_{{a}_{2}}^{+}\right)}^{q}\right)+\left({\left({v}_{{a}_{1}}^{+}\right)}^{q}\vee {\left({v}_{{a}_{2}}^{+}\right)}^{q}\right)}},$$

where the similarity is defined as: the sum of the minimum values of ${u}_{{a}_{1}}^{-}$ and ${u}_{{a}_{2}}^{-},{u}_{{a}_{1}}^{+}$ and ${u}_{{a}_{2}}^{+}$, ${v}_{{a}_{1}}^{-}$ and ${v}_{{a}_{2}}^{-}$, ${v}_{{a}_{1}}^{+}$ and ${v}_{{a}_{2}}^{+}$ divided by the sum of the maximum values between them. The similarity satisfies the four properties:

(S1) $$0\le S\left({a}_{1},{a}_{2}\right)\le 1$$;

(S2) $S\left({a}_{1},{a}_{2}\right)=1$ if and only if ${a}_{1}={a}_{2}$;

(S3) $$S\left({a}_{1},{a}_{2}\right)=S\left({a}_{2},{a}_{1}\right)$$;

(S4) if $\left[{u}_{1}^{-},{u}_{1}^{+}\right]\subseteq \left[{u}_{2}^{-},{u}_{2}^{+}\right]\subseteq \left[{u}_{3}^{-},{u}_{3}^{+}\right]$ and $\left[{v}_{1}^{-},{v}_{1}^{+}\right]\subseteq \left[{v}_{2}^{-},{v}_{2}^{+}\right]\subseteq \left[{v}_{3}^{-},{v}_{3}^{+}\right]$, then $S\left({a}_{1},{a}_{3}\right)\le S\left({a}_{1},{a}_{2}\right)$ and $S\left({a}_{1},{a}_{3}\right)\le S\left({a}_{2},{a}_{3}\right)$.

Equation (9) clearly shows that when the two IVq-ROFNs are farther apart, the similarity is smaller. Otherwise, the similarity is greater. When the two IVq-ROFNs are the same, the similarity is 1.

### Score function

When solving MADM and MAGDM problems under the IVIFSs’ and IVq-ROFSs’ environments, target alternatives often need to be sorted and selected. While the results of the aggregating operators and decision-making methods are IVIFNs or IVq-ROFNs, researchers often use score functions to transform the results into crisp numbers. Although the score function proposed by researchers can be used to compare IVIFNs and IVq-ROFNs, there are also deficiencies with these approaches. The following examples are given to illustrate.

#### Definition 7

 [67] Let $a=(\left[{u}^{-},{u}^{+}\right],\left[{v}^{-},{v}^{+}\right])$ be an IVIFNs, its score function ${S}_{X}$ is

10 $${S}_{X}\left(a\right)=\frac{{u}^{-}+{u}^{+}-{v}^{-}-{v}^{+}}{2},$$

where ${S}_{X}$ represents membership subtracting non-membership and can express the attitude of decision-makers. When ${u}^{-}+{u}^{+}={v}^{-}+{v}^{+}$, Xu [67] proposed the accuracy function ${{H}_{X}\left(\alpha \right)={\frac{1}{2}}(u}^{-}+{u}^{+}+{v}^{-}+{v}^{+})$, which has been widely used in MADM and MAGDM problems under IVIFS environments. If $q=1$, ${S}_{X}$ can be directly used to compare the IVq-ROFNs. If $q>1$, it also can be sometimes used to compare the IVq-ROFNs.

#### Example 1

 Given two IVIFNs, ${a}_{1}=\left([0.811, 0.865], [0.692, 0.789]\right)$ and ${a}_{2}=\left([0.676, 1.0], [0.655, 0.826]\right)$, we have ${S}_{X}\left({a}_{1}\right)={S}_{X}\left({a}_{2}\right)=0.0975$. That is to say that ${S}_{X}$ fails to compare ${a}_{1}$ and ${a}_{2}$. In addition, ${H}_{X}\left({a}_{1}\right)={H}_{X}\left({a}_{2}\right)=1.5785$ indicates that ${H}_{X}$ also fails to compare ${a}_{1}$ and ${a}_{2}$.

Based on Xu’s score function, Liu and Wang proposed a new score function for IVq-ROFN which has been also proved useful for some MADM and MAGDM problems, as shown in Definition 8.

#### Definition 8

[38] Let $a=(\left[{u}^{-},{u}^{+}\right],\left[{v}^{-},{v}^{+}\right])$ be an IVq-ROFN, $q\ge 1$, its score function ${S}_{L}$ is

11 $${S}_{L}\left(a\right)={\frac{{\left({u}^{-}\right)}^{q}+{\left({u}^{+}\right)}^{q}-{\left({v}^{-}\right)}^{q}-{\left({v}^{+}\right)}^{q}}{2}}$$

In Eq. (11), ${S}_{L}$ represents membership subtracting non-membership which can express the attitude of decision-makers. When $({u}^{-}{)}^{q}+({u}^{+}{)}^{q}=({v}^{-}{)}^{q}+({v}^{+}{)}^{q}$, Liu and Wang [38] suggest using the accuracy function ${H}_{L}\left(\alpha \right)={\frac{1}{2}}[({u}^{-}{)}^{q}+({u}^{+}{)}^{q}+({v}^{-}{)}^{q}+({v}^{+}{)}^{q}]$. This approach has been also used in MADM and MAGDM problems under IVq-ROFS environments in recent years.

#### Example 2

Given two IVq-ROFNs ${a}_{3}=\left([{0.134,0.183}],[{0.172,0.859}]\right)$ and ${a}_{4}=\left([{0.066,0.217}],[{0.584,0.653}]\right)$, when $q=2$, ${S}_{L}\left({a}_{3}\right)={S}_{L}\left({a}_{4}\right)=-0.35801.\, {\text{It means that }}{S}_{L}$ fails to compare ${a}_{3}$ and ${a}_{4}$. In addition, ${H}_{L}\left({a}_{3}\right)={H}_{L}\left({a}_{4}\right)=0.409455$, it indicates that the ${H}_{L}\left(\alpha \right)$ also fails to compare ${a}_{3}$ and ${a}_{4}$.

#### Definition 9 

[62] Let $a=\left(\left[{u}^{-},{u}^{+}\left],\right[{v}^{-},{v}^{+}\right]\right)$ be an IVIFN, its score function ${S}_{\text{NWC}}$ is

12 $${S}_{\text{NWC}}\left(a\right)={\frac{\left({u}^{-}+{u}^{+}\right)\left({u}^{-}+{v}^{-}\right)-\left({v}^{-}+{v}^{+}\right)\left({u}^{+}+{v}^{+}\right)}{2}}.$$

When ${u}^{-}+{u}^{+}={v}^{-}+{v}^{+}$, Wang and Chen [62] proposed the accuracy function ${H}_{\text{NWC}}\left(\alpha \right)={\frac{1}{2}}(\left(1- {u}^{-}+{u}^{+}\right)\left( 1-{u}^{-}-{v}^{-}\right)+(1-{v}^{-}+{v}^{+})(1-{v}^{+}-{u}^{+}))$, another approach that has been used in MADM and MAGDM problems under IVIFS environments.

#### Example 3

Given two IVIFNs ${a}_{5}=\left(\left[{0.0,0.0}\right],\left[{0.0,0.0}\right]\right)$ and ${a}_{6}=\left(\left[{0.0,0.1}\right],\left[{0.0,0.0}\right]\right),$ ${S}_{\text{NWC}}\left({a}_{5}\right)={S}_{\text{NWC}}\left({a}_{6}\right)=0$. The ${S}_{\text{NWC}}$ fails to compare ${a}_{5}$ and ${a}_{6}$. ${H}_{\text{NWC}}\left({a}_{5}\right)= {H}_{\text{NWC}}\left({a}_{6}\right)=1$ also indicates a failure of ${H}_{\text{NWC}}\left(\alpha \right)$ in comparing ${a}_{5}$ and ${a}_{6}$.

#### Definition 10 

[20] Let $a=\left(\left[{u}^{-},{u}^{+}\left],\right[{v}^{-},{v}^{+}\right]\right)$ be an IVIFN. Its score function ${S}_{\text{GM}}$ is

13 $${S}_{\text{GM}}\left(a\right)={\frac{{v}^{+}+{v}^{-}-{u}^{+}-{u}^{-}}{2}+\frac{{u}^{-}+{u}^{+}+2\left({u}^{-}{u}^{+}-{v}^{-}{v}^{+}\right)}{{u}^{-}+{u}^{+}+{v}^{-}+{v}^{+}}}.$$

Gong and Ma proposed the accuracy function ${H}_{\text{GM}}=\left({u}_{a}^{+}+{v}_{a}^{+}\right)-0.5({\frac{{{(u}^{+}-{u}^{-})}^{2}}{{u}^{+}}}+{\frac{{{(v}^{+}-{v}^{-})}^{2}}{{v}^{+}}})$. When ${u}^{-}={u}^{+}={v}^{-}={v}^{+}=0, {S}_{\text{GM and}}$ ${H}_{\text{GM}}$ are unreasonable.

#### Example 4

Given two IVIFNs, ${a}_{7}=\left(\left[0, 0.4\right],\left[0, 0.4\right]\right)$ and ${a}_{8}=\left(\left[{0.2,0.2}\right],\left[{0.2,0.2}\right]\right)$, ${S}_{\text{GM}}\left({a}_{7}\right)$= ${S}_{\text{GM}}\left({a}_{8}\right)=0.5$ means that the ${S}_{\text{GM}}$ fails to compare ${a}_{7}$ and ${a}_{8}$. The ${H}_{\text{GM}}$ also fails to compare ${a}_{7}$ and ${a}_{8}$ since ${H}_{\text{GM}}\left({a}_{7}\right)$= ${H}_{\text{GM}}\left({a}_{8}\right)=0.4$.

## Proposed score function and operators

In this section, we define a new score function to rank IVq-ROFNs and some operators to aggregate the information.

### New score function

From the examples of the score functions outlined in “Score function”, the deficiencies of score functions are described. In order to overcome these deficiencies, we developed a new score function, as shown in Definition 11.

#### Definition 11

Let $a=(\left[{{u}_{a}}^{-},{{u}_{a}}^{+}\right],\left[{{v}_{a}}^{-},{{v}_{a}}^{+}\right])$ be an IVq-ROFN, the score function developed as follows:

14 $$\begin{array}{ll}{S}_{c}\left(a\right)&= {\frac{1}{4}}\left(\begin{array}{l}ln\left({u}_{a}^{-}+{u}_{a}^{+}+{v}_{a}^{-}+{v}_{a}^{+}+1\right)+2\times \left(\left({u}_{a}^{-}+{u}_{a}^{+}\right)-\left({v}_{a}^{-}+{v}_{a}^{+}\right)\right)\\ +(\left({u}_{a}^{+}-{u}_{a}^{-}\right)+({v}_{a}^{+}-{v}_{a}^{-}))+\left(({u}_{a}^{+}-{u}_{a}^{-})-({v}_{a}^{+}-{v}_{a}^{-})\right)\\ -Sign\left({u}_{a}^{-}+{u}_{a}^{+}+{v}_{a}^{-}+{v}_{a}^{+}\right)\times ln3\end{array}\right)\end{array}$$

In Eq. (14), the term $$({u}_{a}^{-}+{u}_{a}^{+}+{v}_{a}^{-}+{v}_{a}^{+})$$ expresses the sum of membership and non-membership, which is a certain amount of certainty. $${(u}_{a}^{+}-{u}_{a}^{-})$$ represents the uncertainty of membership, and ($${v}_{a}^{+}-{v}_{a}^{-}$$) represents the uncertainty of non-membership. Similarly, (($${u}_{a}^{-}+{u}_{a}^{+})-{(v}_{a}^{-}+{v}_{a}^{+}))$$ is the difference between membership and non-membership, and (($${u}_{a}^{-}-{u}_{a}^{+})-({v}_{a}^{+}{-v}_{a}^{-}))$$ is the difference of uncertainty between membership and non-membership. The Sign function is a signal function. When $${u}_{a}^{-}+{u}_{a}^{+}+{v}_{a}^{-}+{v}_{a}^{+}=0$$, the result of Sign is 0 and otherwise 1. It is used to keep the result of $${S}_{c}$$ belonging to [− 1, 1].

#### Theorem 1

*Let* $a=\left(\left[{u}_{a}^{-},{u}_{a}^{+}\left],\right[{v}_{a}^{-},{v}_{a}^{+}\right]\right)$ *be an IVq-ROFN, the *${S}_{c}$ *has the following properties*:

1. $$-1\le {S}_{c}(a)\le 1$$;
2. $${S}_{c}\left({a}_{\text{min}}\right)=-1$$ if $${a}_{\text{min}}=([\text{0,0}],[{1,1}])$$;
3. $${S}_{c}({a}_{\text{max}})=1$$ if $${a}_{\text{max}}=([{1,1}],[{0,0}])$$;
4. $${S}_{c}({a}_{\text{mid}})=0$$ if $${a}_{\text{mid}}=([{0,0}],[{0,0}])$$.

#### ***Proof***

Substituting ${a}_{\text{min}}=([{0,0}],[{1,1}])$, ${a}_{\text{max}}=([{1,1}],[{0,0}])$ and ${a}_{\text{mid}}=([{0,0}],[{0,0}])$ into Eq. (14), we know that ${S}_{c}\left({a}_{\text{min}}\right)=-1$, ${S}_{c}({a}_{\text{max}})=1$ and ${S}_{c}({a}_{\text{mid}})=0$. Consequently, the properties (2), (3) and (4) hold.

The partial derivatives of ${u}_{a}^{-}$, ${u}_{a}^{+}$, ${v}_{a}^{-}$ and ${v}_{a}^{+}$ are

$$\begin{array}{ll}&{\frac{\partial
{S}_{c}}{\partial {u}_{a}^{-}}}={\frac{1}{4}}\times
\left({\frac{1}{{u}_{a}^{-}+{u}_{a}^{+}+{v}_{a}^{-}+{v}_{a}^{+}+1}}
\right)>0,\\
& {\frac{\partial {S}_{c}}{\partial
{u}_{a}^{+}}}={\frac{1}{4}}\times
\left({\frac{1}{{u}_{a}^{-}+{u}_{a}^{+}+{v}_{a}^{-}+{v}_{a}^{+}+1}}+4\right)>0,\\
&\frac{\partial {S}_{c}}{\partial {v}_{a}^{-}}=\frac{1}{4}\times
\left(\frac{1}{{u}_{a}^{-}+{u}_{a}^{+}+{v}_{a}^{-}+{v}_{a}^{+}+1}-2\right)<0,\\
&
\frac{\partial {S}_{c}}{\partial {v}_{a}^{+}}=\frac{1}{4}\times
\left(\frac{1}{{u}_{a}^{-}+{u}_{a}^{+}+{v}_{a}^{-}+{v}_{a}^{+}+1}-2\right)<0.\end{array}$$

It can be seen that ${u}_{a}^{-}$, ${u}_{a}^{+}$, ${v}_{a}^{-}$ and ${v}_{a}^{+}$ are monotonic. Specifically, ${u}_{a}^{-}$ and ${u}_{a}^{+}$ are monotonically increasing and ${v}_{a}^{-}$ and ${v}_{a}^{+}$ are monotonically decreasing. For any IVq-ROFN $a$, ${{S}_{c}({a}_{\text{min}})\le {S}}_{c}\left(a\right)\le {{S}}_{c}({a}_{\text{max}})$ and $-1\le {S}_{c}(a)\le 1$, Property (1) holds.

According to Theorem 1, for any two IVq-ROFNs, membership is monotonically increasing and non-membership is monotonically decreasing. Thus, IVq-ROFNs can be compared using Definition 12.

#### Definition 12

Given the two IVq-ROFNs ${a}_{1}=(\left[{u}_{{a}_{1}}^{-},{u}_{{a}_{1}}^{+}\right],\left[{v}_{{a}_{1}}^{-},{v}_{{a}_{1}}^{+}\right])$ and ${a}_{2}=(\left[{u}_{{a}_{2}}^{-},{u}_{{a}_{2}}^{+}\right],$ $\left[{v}_{{a}_{2}}^{-},{v}_{{a}_{2}}^{+}\right])$, their comparison laws are

1. If $${S}_{c}\left({a}_{1}\right)>{S}_{c}\left({a}_{2}\right)$$, then $${a}_{1}>{a}_{2}$$;
2. If $${S}_{c}\left({a}_{1}\right)<{S}_{c}\left({a}_{2}\right)$$, then $${a}_{1}<{a}_{2}$$;
3. If $${S}_{c}\left({a}_{1}\right)={S}_{c}\left({a}_{2}\right)$$, then $${a}_{1}={a}_{2}$$.

#### Example 5

${S}_{c}$ is used to calculate the four groups of data in Examples 1–4. The results are shown in Table 1.

**Table 1:** The comparison results derived by the score function of IVq-ROFNs

Score IVq-ROFNs	${S}_{X}$	${S}_{L}$	${S}_{\text{NWC}}$	${S}_{\text{GM}}$	${S}_{c}$
$${a}_{1}=\left(\begin{array}{l}\left[{0.811,0.865}\right], [0.692, 0.789]\end{array}\right)$$ $${a}_{2}=\left(\begin{array}{l}\left[{0.676,1.000}\right], [{0.655,0.826}]\end{array}\right)$$	${S}_{X}\left({a}_{1}\right)=0.0975$ ${S}_{X}\left({a}_{2}\right)=0.0975$	${S}_{L}\left({a}_{1}\right)=0.1523$ ${S}_{L}\left({a}_{2}\right)=0.1728$	${S}_{\text{NWC}}\left({a}_{1}\right)=0.0347$ ${S}_{\text{NWC}}\left({a}_{2}\right)=-0.2368$	${S}_{\text{GM}}\left({a}_{1}\right)=0.5319$ ${S}_{\text{GM}}\left({a}_{2}\right)=0.5189$	${S}_{c}\left({a}_{1}\right)=0.206$ ${S}_{c}\left({a}_{2}\right)=0.341$
	${S}_{X}\left({a}_{1}\right)={S}_{X}\left({a}_{2}\right)$ *Incommensurable*	${S}_{L}\left({a}_{1}\right)<{S}_{L}\left({a}_{2}\right)$	${S}_{\text{NWC}}\left({a}_{1}\right)>{S}_{\text{NWC}}\left({a}_{2}\right)$	${S}_{\text{GM}}\left({a}_{1}\right)>{S}_{\text{GM}}\left({a}_{2}\right)$	${S}_{c}\left({a}_{1}\right)<{S}_{c}\left({a}_{2}\right)$
$${a}_{3}=\left(\begin{array}{l}\left[{0.134,0.183}\right], [{0.172,0.859}]\end{array}\right)$$ $${a}_{4}=\left(\begin{array}{l}\left[{0.066,0.217}\right], [{0.584,0.653}]\end{array}\right)$$	${S}_{X}\left({a}_{3}\right)=-0.357$ ${S}_{X}\left({a}_{4}\right)=-0.477$	${S}_{L}\left({a}_{3}\right)=-0.358$ ${S}_{L}\left({a}_{4}\right)=-0.358$	${S}_{\text{NWC}}\left({a}_{3}\right)=-0.4886$ ${S}_{\text{NWC}}\left({a}_{4}\right)=-0.4461$	${S}_{\text{GM}}\left({a}_{3}\right)=0.4093$ ${S}_{\text{GM}}\left({a}_{4}\right)=0.1803$	${S}_{c}\left({a}_{3}\right)=-0.3938$ ${S}_{c}\left({a}_{4}\right)=-0.4451$
	${S}_{X}\left({a}_{1}\right)>{S}_{X}\left({a}_{2}\right)$	${S}_{L}\left({a}_{3}\right)={S}_{L}\left({a}_{4}\right)$ *Incommensurable*	${S}_{\text{NWC}}\left({a}_{1}\right)<{S}_{\text{NWC}}\left({a}_{2}\right)$	${S}_{\text{GM}}\left({a}_{3}\right)>{S}_{\text{GM}}\left({a}_{4}\right)$	${S}_{c}\left({a}_{3}\right)>{S}_{c}\left({a}_{4}\right)$
$${a}_{5}=\left(\begin{array}{l}\left[{0.000,0.000}\right], \left[{0.000,0.000}\right]\end{array}\right)$$ $${a}_{6}=\left(\begin{array}{l}\left[{0.000,0.100}\right], \left[{0.000,0.000}\right]\end{array}\right)$$	${S}_{X}\left({a}_{5}\right)=0$ ${S}_{X}\left({a}_{6}\right)=0.05$	${S}_{L}\left({a}_{5}\right)=0$ ${S}_{L}\left({a}_{6}\right)=0.005$	${S}_{\text{NWC}}\left({a}_{5}\right)=0$ ${S}_{\text{NWC}}\left({a}_{6}\right)=0$	${S}_{\text{GM}}\left({a}_{5}\right)=NULL$ ${S}_{\text{GM}}\left({a}_{6}\right)=0.95$	${S}_{c}\left({a}_{5}\right)=0$ ${S}_{c}\left({a}_{6}\right)=-0.1508$
	${S}_{X}\left({a}_{5}\right)<{S}_{X}\left({a}_{6}\right)$	${S}_{L}\left({a}_{5}\right)<{S}_{L}\left({a}_{6}\right)$	${S}_{\text{NWC}}\left({a}_{5}\right)={S}_{\text{NWC}}\left({a}_{6}\right)$ *Incommensurable*	*Incommensurable*	${S}_{c}\left({a}_{5}\right)>{S}_{c}\left({a}_{6}\right)$
$${a}_{7}=\left(\begin{array}{l}\left[0.000, 0.400\right], \left[{0.000,0.400}\right]\end{array}\right)$$ $${a}_{8}=\left(\begin{array}{l}\left[{0.200,0.200}\right], \left[{0.200,0.200}\right]\end{array}\right)$$	${S}_{X}\left({a}_{7}\right)=0$ ${S}_{X}\left({a}_{8}\right)=0$	${S}_{L}\left({a}_{7}\right)=0$ ${S}_{L}\left({a}_{8}\right)=0$	${S}_{\text{NWC}}\left({a}_{7}\right)=-0.16$ ${S}_{\text{NWC}}\left({a}_{8}\right)= 0$	${S}_{\text{GM}}\left({a}_{7}\right)=0.5$ ${S}_{\text{GM}}\left({a}_{8}\right)=0.5$	${S}_{c}\left({a}_{7}\right)= 0.0723$ ${S}_{c}\left({a}_{8}\right)=-0.1277$
	${S}_{X}\left({a}_{7}\right)={S}_{X}\left({a}_{8}\right)$ *Incommensurable*	${S}_{L}\left({a}_{7}\right)={S}_{L}\left({a}_{8}\right)$ *Incommensurable*	${S}_{\text{NWC}}\left({a}_{7}\right)<{S}_{\text{NWC}}\left({a}_{8}\right)$	${S}_{\text{GM}}\left({a}_{7}\right)={S}_{\text{GM}}\left({a}_{8}\right)$ *Incommensurable*	${S}_{c}\left({a}_{7}\right)>{S}_{c}\left({a}_{8}\right)$

As can be seen from Table 1, the proposed score function overcomes the deficiency and can better distinguish IVq-ROFNs. However, ${S}_{X}$ cannot compare ${a}_{1}$ and ${a}_{2}$, ${a}_{7}$ and ${a}_{8}$, ${S}_{L}$ cannot compare ${a}_{3}$ and ${a}_{4}$, ${a}_{7}$ and ${a}_{8}$, ${S}_{\text{NWC}}$ cannot compare ${a}_{5}$ and ${a}_{6}$, and ${S}_{\text{GM}}$ cannot compare ${a}_{5}$ and ${a}_{6}$, ${a}_{7}$ and ${a}_{8}$. In order to illustrate its advantages and show better adaptability to various environments, we designed four cases to test the ${S}_{c}$ function.

#### Example 6.

**Fig. 1:**
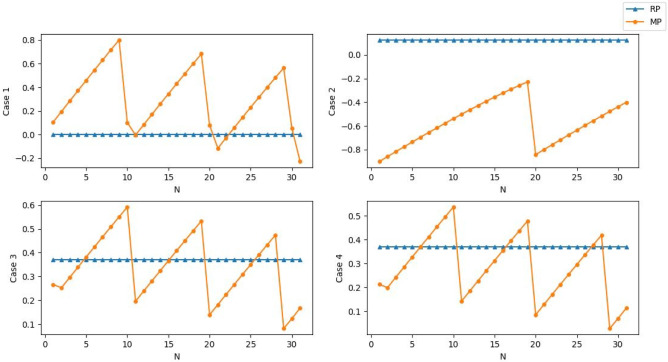
Score function value analysis

We design four cases to test ${{S}}_{c}$. Let ${a}_{1}$ and ${a}_{2}$ be two IVq-ROFNs and ${a}_{1}$ be a fixed point. ${a}_{2}$ changes from ([1,1], [0,0]) to ([0,0], [1,1]) by a 0.05 step (move point, MP). The scores of ${a}_{1}$ and ${a}_{2}$ are presented by RP and MP in Fig. 1: (1) Case 1. The interval length of ${a}_{1}$ and ${a}_{2}$ is 0, and ${a}_{1}=([{0.5,0.5}],[{0.5,0.5}])$. (2) Case 2. The interval length of ${a}_{1}$ and ${a}_{2}$ is 0, and ${a}_{1}$ is randomly generated. (3) Case 3. The interval length of ${a}_{1}$ and ${a}_{2}$ is the same but not equal to 0. (4) Case 4. The interval length of ${a}_{1}$ is larger than that of ${a}_{2}$ and neither of them equals 0.

As shown in Fig. 1, random IVq-ROFNs are generated to simulate the four cases. The scores of ${a}_{1}$ and ${a}_{2}$ have just one coincidence point where ${a}_{1}={a}_{2}$. Therefore, the proposed score function can be used to distinguish different IVq-ROFNs.

### Evaluation indices of the HFU system

The high cost of adopting ISO/IEC standards in the application of software quality assessment [28] means they cannot be used to meet the needs of small and medium-sized enterprises. Even large organizations like healthcare agencies are often not able to afford to adopt such standards, especially if they are only required by an individual unit. HFU systems have been developed primarily to help hospitals manage hypertension among diagnosed outpatients. The management systems that many hospitals currently use struggle to control and manage the manifestations of this condition within a mobile and scattered population. Hypertension software systems, however, can improve the detection of blood pressure changes and can help to control them. More importantly, they can do this for a scattered and mobile outpatient population. To justify the expense associated with purchasing such a system, community hospitals need to resolve two contradictions. First, patients have limited ability to prevent their symptoms. They often cannot effectively manage their blood pressure or timely get hospital treatment. Second, primary healthcare facilities do not have the resources to track and monitor all their outpatients and have no way to escalate the treatment of those patients in need. Any remote management system, therefore, needs to (1) allow outpatients to access follow-up medical services from any location at any time and (2) enable medical staff to provide hypertension management services to outpatients in any location at any time.

Any HFU system should, first, be able to meet the requirements of function, performance, safety, and reliability. It should be an acceptable cost and easy-to-use, both for outpatients and medical staff. Second, for the intercommunication of other software, the purchased system needs to meet the needs of community hospitals in terms of scalability [70], integration [43], reliability [6, 78] and compatibility [19], and reduces the hospital's future cost expenditures. Third, a management system should meet a number of post-purchase criteria: the stability of the supplier [6, 49], the supplier's follow-up service [6], and the likely extent of daily maintenance [4, 50]. Inspired by the existing literature on hypertension management systems [4, 6, 10, 11, 28, 70, 77], an evaluation of the effectiveness of a community HFU system evaluation should encompass 13 indices: (1) Cost (C1), (2) Performance (C2), (3) Reliability (C3), (4) Security (C4), (5) Function (C5), (6) Easy-to-use (C6), (7) Extensibility (C7), (8) Compatibility (C8), (9) Deployment time (C9), (10) Integration (C10), (11) Supplier stability (C11), (12) Follow-up service (C12), and (13) Maintainability (C13). A detailed explanation of each index is shown in Table 2.

**Table 2:** Indices for the evaluation of the HFU systems

Reference	Code	System index	System index definition
ISO/IEC 25001 [28], Büyüközkan and Göçer [6]	${C}_{1}$	Cost	The software cost is reasonable and does not exceed the hospital’s budget
Yuen and Lau [77], Bertoa et al. [4], Dayanandan and Kalimuthu [10]	${C}_{2}$	Performance	The system supports simultaneous access by multiple users in the community. Ordinary hardware servers can meet the requirements of community hospitals
Büyüközkan and Göçer [6], Zarzour and Rekab [78]	${C}_{3}$	Reliability	There will be no bugs. Even if there are bugs, they will not hinder the use of other functions
Deb and Roy [11], Al-Zahrani [1]	${C}_{4}$	Security	Security will be present across systems, data and networks
Yan et al. [70], Mahmudova and Jabrailova [41], Büyüközkan and Göçer [6], Bertoa et al. [4]	${C}_{5}$	Function	Functionality will include intelligent blood pressure measurement equipment, data sharing, monitoring, remote implementation of voice and video interaction, abnormal value reminders, periodic daily reminders, service promotion, system maintenance, and user management. The system will support PC and App access
Bertoa et al. [4], Büyüközkan and Göçer [6]	${C}_{6}$	Easy-to-use	Both desktop and app versions of the system are easy-to-use for both outpatients and medical staff
Yan et al. [70]	${C}_{7}$	Extensibility	There will be support for future device access and data extraction from other systems
Bertoa et al. [4], Büyüközkan and Göçer [6], Geng et al. [19]	${C}_{8}$	Compatibility	Is it compatible with existing operating systems, databases, and other systems?
Büyüközkan and Göçer [6]	${C}_{9}$	Deployment time	After the purchase, how long from deployment to implementation?
Yan et al. [70], Mehlawat et al. [43], Bertoa et al. [4]	${C}_{10}$	Integration	Integration with other systems in community hospitals
Büyüközkan and Göçer [6], Pan and Chai [49]	${C}_{11}$	Supplier stability	The longevity of software vendors in the market
Büyüközkan and Göçer [6]	${C}_{12}$	Follow-up service	Follow-up service: response time, service attitude, service quality
Bertoa et al. [4], Büyüközkan and Göçer [6], Peercy [50], Bertoa et al. [4]	${C}_{13}$	Maintainability	Ease of maintenance, data and log backup, data monitoring, and abnormal reminder

Table 2 outlines the full range of indices required for any viable and usable management system. Some of these indices reflect the possible cost to a hospital and others reflect the potential benefit that the adoption of a management system might involve.

### IVq-ROFWFA operations

Inspired by operations of q-ROFWFA [55], the multiplication and scalar multiplication of IVq-ROFNs are developed. Definition 13 proposes the properties of the IVq-ROFWFA operation.

#### Definition 13

Let ${a}_{1}=\left(\left[{u}_{{a}_{1}}^{-},{u}_{{a}_{1}}^{+}\left],\right[{v}_{{a}_{1}}^{-},{v}_{{a}_{1}}^{+}\right]\right)$ and ${a}_{2}=\left(\left[{u}_{{a}_{2}}^{-},{u}_{{a}_{2}}^{+}\left],\right[{v}_{{a}_{2}}^{-},{v}_{{a}_{2}}^{+}\right]\right)$ be two IVq-ROFNs, and $q\ge 1$, ${\lambda }_{F}>0$. The multiplication and scalar multiplication of IVq-ROFN are defined as follows:

15 $${a}_{1}{\otimes }_{F}{a}_{2}=\left(\begin{array}{l}\left[\begin{array}{l}{\left(\left(\frac{{\left({u}_{{a}_{1}}^{-}\right)}^{q}{\left({u}_{{a}_{2}}^{-}\right)}^{q}}{{\left({u}_{{a}_{1}}^{-}\right)}^{q}{\left({u}_{{a}_{2}}^{-}\right)}^{q}+{\left({v}_{{a}_{1}}^{-}\right)}^{q}{\left({v}_{{a}_{2}}^{-}\right)}^{q}}\right)\times (1-(1-{\left({u}_{{a}_{1}}^{-}\right)}^{q}-{\left({v}_{{a}_{1}}^{-}\right)}^{q})(1-{\left({u}_{{a}_{2}}^{-}\right)}^{q}-{\left({v}_{{a}_{2}}^{-}\right)}^{q}))\right)}^{\frac{1}{q}},\\ {\left(\left(\frac{{\left({u}_{{a}_{1}}^{+}\right)}^{q}{\left({u}_{{a}_{2}}^{+}\right)}^{q}}{{\left({u}_{{a}_{1}}^{+}\right)}^{q}{\left({u}_{{a}_{2}}^{+}\right)}^{q}+{\left({v}_{{a}_{1}}^{+}\right)}^{q}{\left({v}_{{a}_{2}}^{+}\right)}^{q}}\right)\times (1-(1-{\left({u}_{{a}_{1}}^{+}\right)}^{q}-{\left({v}_{{a}_{1}}^{+}\right)}^{q})(1-{\left({u}_{{a}_{2}}^{+}\right)}^{q}-{\left({v}_{{a}_{2}}^{+}\right)}^{q}))\right)}^{\frac{1}{q}}\end{array}\right],\\ \left[\begin{array}{l}{\left(\left(\frac{{\left({v}_{{a}_{1}}^{-}\right)}^{q}{\left({v}_{{a}_{2}}^{-}\right)}^{q}}{{\left({u}_{{a}_{1}}^{-}\right)}^{q}{\left({u}_{{a}_{2}}^{-}\right)}^{q}+{\left({v}_{{a}_{1}}^{-}\right)}^{q}{\left({v}_{{a}_{2}}^{-}\right)}^{q}}\right)\times (1-(1-{\left({u}_{{a}_{1}}^{-}\right)}^{q}-{\left({v}_{{a}_{1}}^{-}\right)}^{q})(1-{\left({u}_{{a}_{2}}^{-}\right)}^{q}-{\left({v}_{{a}_{2}}^{-}\right)}^{q}))\right)}^{\frac{1}{q}},\\ {\left(\left(\frac{{\left({v}_{{a}_{1}}^{+}\right)}^{q}{\left({v}_{{a}_{2}}^{+}\right)}^{q}}{{\left({u}_{{a}_{1}}^{+}\right)}^{q}{\left({u}_{{a}_{2}}^{+}\right)}^{q}+{\left({v}_{{a}_{1}}^{+}\right)}^{q}{\left({v}_{{a}_{2}}^{+}\right)}^{q}}\right)\times (1-(1-{\left({u}_{{a}_{1}}^{+}\right)}^{q}-{\left({v}_{{a}_{1}}^{+}\right)}^{q})(1-{\left({u}_{{a}_{2}}^{+}\right)}^{q}-{\left({v}_{{a}_{2}}^{+}\right)}^{q}))\right)}^{\frac{1}{q}}\end{array}\right]\end{array}\right)$$

16 $${\lambda }_{F}{a}_{1}=\left(\begin{array}{l}\left[{\left(\left(\frac{{\left({u}_{{a}_{1}}^{-}\right)}^{q\lambda }}{{\left({u}_{{a}_{1}}^{-}\right)}^{q\lambda }+{\left({v}_{{a}_{1}}^{-}\right)}^{q\lambda }}\right)\times (1-(1-{\left({u}_{{a}_{1}}^{-}\right)}^{q}-{\left({v}_{{a}_{1}}^{-}\right)}^{q}{)}^{\lambda })\right)}^{\frac{1}{q}},{\left(\left(\frac{{\left({u}_{{a}_{1}}^{+}\right)}^{q\lambda }}{{\left({u}_{{a}_{1}}^{+}\right)}^{q\lambda }+{\left({v}_{{a}_{1}}^{+}\right)}^{q\lambda }}\right)\times (1-(1-{\left({u}_{{a}_{1}}^{+}\right)}^{q}-{\left({v}_{{a}_{1}}^{+}\right)}^{q}{)}^{\lambda })\right)}^{\frac{1}{q}}\right],\\ \left[{\left(\left(\frac{{\left({v}_{{a}_{1}}^{-}\right)}^{q\lambda }}{{\left({u}_{{a}_{1}}^{-}\right)}^{q\lambda }+{\left({v}_{{a}_{1}}^{-}\right)}^{q\lambda }}\right)\times (1-(1-{\left({u}_{{a}_{1}}^{-}\right)}^{q}-{\left({v}_{{a}_{1}}^{-}\right)}^{q}{)}^{\lambda })\right)}^{\frac{1}{q}},{\left(\left(\frac{{\left({v}_{{a}_{1}}^{+}\right)}^{q\lambda }}{{\left({u}_{{a}_{1}}^{+}\right)}^{q\lambda }+{\left({v}_{{a}_{1}}^{+}\right)}^{q\lambda }}\right)\times (1-(1-{\left({u}_{{a}_{1}}^{+}\right)}^{q}-{\left({v}_{{a}_{1}}^{+}\right)}^{q}{)}^{\lambda })\right)}^{\frac{1}{q}}\right]\end{array}\right).$$

According to Eqs. (15) and (16), the result obtained by ${a}_{1}{\otimes }_{F}{a}_{2}$ and ${\lambda }_{F}{a}_{1}$ is still an IVq-ROFN.

#### Proposition 1

*Let* ${a}_{1}=\left(\left[{u}_{{a}_{1}}^{-},{u}_{{a}_{1}}^{+}\right],\left[{v}_{{a}_{1}}^{-},{v}_{{a}_{1}}^{+}\right]\right)$ *and* $a_{2} = \left( {\left[ {u_{{a_{2} }}^{ - } ,u_{{a_{2} }}^{ + } } \right],\left[ {v_{{a_{2} }}^{ - } ,v_{{a_{2} }}^{ + } } \right]} \right)$ *be two IVq-ROFNs. If* ${u}_{{a}_{1}}={v}_{{a}_{1}}$ *and* ${u}_{{a}_{2}}={v}_{{a}_{2}}$ *, then*

1. $${u}_{{a}_{1}{\otimes }_{F}{a}_{2}}=  {v}_{{a}_{1}{\otimes }_{F}{a}_{2}},$$
2. $${u}_{{\lambda }_{F}{a}_{1}}= {v}_{{\lambda }_{F}{a}_{1}}.$$

The above proposition shows that when the membership and non-membership are initially equal, operations ${a}_{1}{\otimes }_{F}{a}_{2}$ and ${\lambda }_{F}{a}_{1}$ reflect a neutral or fair situation for experts. We are, therefore, calling the ${a}_{1}{\otimes }_{F}{a}_{2}$ and ${\lambda }_{F}{a}_{1}$ neutral operations. Equations (15) and (16) make it easy to deduce that the multiplication and scalar multiplication of IVq-ROFWFA satisfy the commutative law.

### The IVq-ROFWFAWA operator

In this subsection, the definition of the IVq-ROFWFAWA operator is introduced. In addition, its properties are described.

#### Definition 14

Let ${a}_{i}=\left(\left[{u}_{{a}_{i}}^{-},{u}_{{a}_{i}}^{+}\right],\left[{v}_{{a}_{i}}^{-},{v}_{{a}_{i}}^{+}\right]\right)(i={1,2},\dots ,n)$ be a group of IVq-ROFNs and $\omega =({\omega }_{1},{\omega }_{2},\ldots,{\omega }_{n}{)}^{T}$ be a weight vector with $\sum_{i=1}^{n}{w}_{i}=1,{w}_{i}\ge 0,(i={1,2},\dots ,n)$. The definition of the IVq-ROFWFAWA operator is

17 $$ {\text{IVq - ROFWFAWA}}\left( {\alpha _{1} ,\alpha _{2} , \ldots ,\alpha _{n} } \right) = \mathop { \otimes _{F} }\limits_{{i = 1}}^{n} w_{i} \alpha _{i} . $$

#### Theorem 3

*Let *$${a}_{i}=\left(\left[{u}_{{a}_{i}}^{-},{u}_{{a}_{i}}^{+}\right],
\left[{v}_{{a}_{i}}^{-},{v}_{{a}_{i}}^{+}\right]\right)\left(i={1,2},\right.\break \left.\dots
,n\right)$$* be a group of IVq-ROFNs. The result of *$${\text{IVq-ROFWFAWA}}\left({\alpha
}_{1},{\alpha }_{2},\dots ,{\alpha
}_{n}\right)$$* is still an IVq-ROFN, which is shown in *Eq. (18):

18 $$\begin{array}{ll}{\text{IVq-ROFWFAWA}}\left({\alpha
}_{1},{\alpha }_{2},\dots ,{\alpha }_{n}\right)&=
\left(\begin{array}{l}\left[\begin{array}{l}{\left(\frac{{\prod
}_{i=1}^{n}{\left({\left({u}_{{\alpha
}_{i}}^{-}\right)}^{q}\right)}^{{w}_{i}}}{{\prod
}_{i=1}^{n}{\left({\left({u}_{{\alpha
}_{i}}^{-}\right)}^{q}\right)}^{{w}_{i}}+{\prod
}_{i=1}^{n}{\left({\left({v}_{{\alpha
}_{i}}^{-}\right)}^{q}\right)}^{{w}_{i}}}\times \left(1-{\prod
}_{i=1}^{n}{\left(1-{\left({u}_{{\alpha
}_{i}}^{-}\right)}^{q}-{\left({v}_{{\alpha
}_{i}}^{-}\right)}^{q}\right)}^{{w}_{i}}\right)\right)}^{\frac{1}{q}},\\
{\left(\frac{{\prod
}_{i=1}^{n}{\left({\left({u}_{{\alpha
}_{i}}^{+}\right)}^{q}\right)}^{{w}_{i}}}{{\prod
}_{i=1}^{n}{\left({\left({u}_{{\alpha
}_{i}}^{+}\right)}^{q}\right)}^{{w}_{i}}+{\prod
}_{i=1}^{n}{\left({\left({v}_{{\alpha
}_{i}}^{+}\right)}^{q}\right)}^{{w}_{i}}}\times \left(1-{\prod
}_{i=1}^{n}{\left(1-{\left({u}_{{\alpha
}_{i}}^{+}\right)}^{q}-{\left({v}_{{\alpha
}_{i}}^{+}\right)}^{q}\right)}^{{w}_{i}}\right)\right)}^{\frac{1}{q}}\end{array}\right],\\
\left[\begin{array}{l}{\left(\frac{{\prod
}_{i=1}^{n}{\left({\left({v}_{{\alpha
}_{i}}^{-}\right)}^{q}\right)}^{{w}_{i}}}{{\prod
}_{i=1}^{n}{\left({\left({u}_{{\alpha
}_{i}}^{-}\right)}^{q}\right)}^{{w}_{i}}+{\prod
}_{i=1}^{n}{\left({\left({v}_{{\alpha
}_{i}}^{-}\right)}^{q}\right)}^{{w}_{i}}}\times \left(1-{\prod
}_{i=1}^{n}{\left(1-{\left({u}_{{\alpha
}_{i}}^{-}\right)}^{q}-{\left({v}_{{\alpha
}_{i}}^{-}\right)}^{q}\right)}^{{w}_{i}}\right)\right)}^{\frac{1}{q}},\\
{\left(\frac{{\prod
}_{i=1}^{n}{\left({\left({v}_{{\alpha
}_{i}}^{+}\right)}^{q}\right)}^{{w}_{i}}}{{\prod
}_{i=1}^{n}{\left({\left({u}_{{\alpha
}_{i}}^{+}\right)}^{q}\right)}^{{w}_{i}}+{\prod
}_{i=1}^{n}{\left({\left({v}_{{\alpha
}_{i}}^{+}\right)}^{q}\right)}^{{w}_{i}}}\times \left(1-{\prod
}_{i=1}^{n}{\left(1-{\left({u}_{{\alpha
}_{i}}^{+}\right)}^{q}-{\left({v}_{{\alpha
}_{i}}^{+}\right)}^{q}\right)}^{{w}_{i}}\right)\right)}^{\frac{1}{q}}\end{array}\right]\end{array}\right)\end{array}.$$

Using Eqs. (15) and (16), Theorem 3 can be easily deduced and the proof omitted. The IVq-ROFWFAWA operator satisfies idempotency, boundedness, monotonicity and commutativity which are described by Theorems 4, 5, 6 and 7. Using Eqs. (15), (16) and (18), the proof processes can be easily deduced and, therefore, omitted.

#### Theorem 4

(Idempotency) *Let* ${\alpha }_{0}=\left(\left[{u}_{{\alpha }_{0}}^{-},{u}_{{\alpha }_{0}}^{+}\right],\left[{v}_{{\alpha }_{0}}^{-},{v}_{{\alpha }_{0}}^{+}\right]\right)$ *be an IVq-ROFN and* ${\alpha }_{i}=\left(\left[{u}_{{\alpha }_{i}}^{-},{u}_{{\alpha }_{i}}^{+}\right],\left[{v}_{{\alpha }_{i}}^{-},{v}_{{\alpha }_{i}}^{+}\right]\right)\left(i={1,2},\dots ,n\right)$ *be a group of IVq-ROFNs. When* ${\alpha }_{i}={\alpha }_{0}$, Eq. (19) holds:

19 $${\text{IVq-ROFWFAWA}}\left({a}_{1},{a}_{2},\ldots,{a}_{n}\right)={a}_{0}.$$

#### Theorem 5 

(Boundedness) *Let* $A=\left\{{a}_{1},{a}_{2},\ldots ,{a}_{n}\right\}$ *be a group of IVq-ROFNs. If* ${a}_{\text{max}}={\text{max}}_{i=1}^{n}\left\{{a}_{i}\right\}$ *and* ${a}_{\text{min}}={\text{min}}_{i=1}^{n}\left\{{a}_{i}\right\}$, *it is easy to obtain*:

20 $${a}_{\text{min}}\le {\text{IVq-ROFWFAWA}}\left({a}_{1},{a}_{2},\ldots,{a}_{n}\right)\le {a}_{\text{max}}.$$

#### Theorem 6

(Monotonicity) *Let* $${\alpha
}_{i}=\left(\left[{u}_{{\alpha }_{i}}^{-},{u}_{{\alpha
}_{i}}^{+}\right],\right.\break \left.\left[{v}_{{\alpha }_{i}}^{-},{v}_{{\alpha
}_{i}}^{+}\right]\right)$$ *and* $${{\alpha
}_{i}}^{{^{\prime}}}=\left(\left[{u}_{{{\alpha
}_{i}}^{{^{\prime}}}}^{-},{u}_{{{\alpha
}_{i}}^{{^{\prime}}}}^{+}\right],\left[{v}_{{{\alpha
}_{i}}^{{^{\prime}}}}^{-},{v}_{{{\alpha
}_{i}}^{{^{\prime}}}}^{+}\right]\right)\left(i={1,2},\right.\break\left.\ldots,n\right)$$ *be two groups of IVq-ROFNs. For any* $$i:{\alpha }_{i}\le {{\alpha
}_{i}}^{{^{\prime}}}$$, Eq. (21) *holds*:

21 $${\text{IVq-ROFWFAWA}}\left({a}_{1},{a}_{2},\ldots,{a}_{n}\right)<{\text{IVq-ROFWFAWA}}\left({{a}_{1}}^{\prime},{{a}_{2}}^{\prime},\ldots,{{a}_{n}}^{\prime}\right).$$

#### Theorem 7

(Commutativity) *Let *${\alpha }_{i}=\left(\left[{u}_{{\alpha }_{i}}^{-},{u}_{{\alpha }_{i}}^{+}\right],\left[{v}_{{\alpha }_{i}}^{-},{v}_{{\alpha }_{i}}^{+}\right]\right)$* be a group of IVq-ROFNs and *${{\alpha }_{i}}^{\prime}=\left(\left[{u}_{{{\alpha }_{i}}^{\prime}}^{-},{u}_{{{\alpha }_{i}}^{\prime}}^{+}\right],\left[{v}_{{{\alpha }_{i}}^{\prime}}^{-},{v}_{{{\alpha }_{i}}^{\prime}}^{+}\right]\right)$* is then the permutation of *${\alpha }_{i}$, Eq. (22) *holds:*

22 $${\text{IVq-ROFWFAWA}}\left({a}_{1},{a}_{2},\ldots,{a}_{n}\right)=I{Vq}-{ROFWFAWA}\left({{a}_{1}}^{\prime},{{a}_{2}}^{\prime},\ldots,{{a}_{n}}^{\prime}\right).$$

The purpose of the IVq-ROFWFAWA operator is used to aggregate the information of multiple experts and is used to aggregate the alternative information of multiple attributes.

## Integrated group decision method

To make the decision-making process more scientific and reduce the influence of human subjectivity on the results, an integrated group decision-making method is presented. “Group decision environment description” describes the GDM environment. The attribute weights are derived in “Deriving attribute weights”. “Deriving expert weights” describes a strategy for determining the expert weights. “MAGDM method based on ARAS” clarifies the MAGDM method based on the ARAS.

### Group decision environment description

To make selecting the best HFU system more reliable for the hospital, experts who have rich experiences and knowledge are invited to evaluate the supplier's products and have a making-decision. In order to reduce the influence of subjective factors and improve the efficiency of MAGDM, expert or attribute weights are unknown in advance. For this reason, the MAGDM environment should satisfy (1) there are *k* experts and *m* alternatives, (2) each alternative has the same *n* attributes, (3) the expert and attribute weights are incomplete, and (4) the elements of the decision matrix are IVq-ROFNs. The mathematical description of the MAGDM environment is as follows.

Suppose there are $k$ experts, and the expert set is $D=\left\{{D}^{\left(1\right)},{D}^{\left(2\right)},\dots ,{D}^{\left(k\right)}\right\}$. The expert weights are unknown and satisfy ${\lambda }^{\left(t\right)}\ge 0,{\sum }_{t=1}^{k}{\lambda }^{\left(t\right)}=1$. The $m$ alternatives are $X=\left\{{x}_{1},{x}_{2},\dots ,{x}_{m}\right\}$, each of which contains the same $n$ attributes: $C=\left\{{C}_{1},{\text{C}}_{2}, \ldots,{{\text{C}}}_{n}\right\}$. The attribute weights are unknown and satisfy ${\sum }_{j=1}^{n}{w}_{j}=1,{w}_{j}\ge 0$. The decision matrix is ${A}^{{{\prime}}\left(t\right)}=({a}_{ij}^{{{\prime}}\left(t\right)}{)}_{m\times n},\left(i={1,2},\dots ,m; j=1,2,\dots ,n; t={1,2},\ldots,k\right)$. ${a}_{ij}^{{{\prime}}\left(t\right)}=\left([{u}_{{a}_{ij}^{{{\prime}}\left(t\right)}}^{-},{u}_{{a}_{ij}^{{{\prime}}\left(t\right)}}^{+}],[{v}_{{a}_{ij}^{{{\prime}}\left(t\right)}}^{-},{v}_{{a}_{ij}^{{{\prime}}\left(t\right)}}^{+}]\right)$ is an IVq-ROFN. There exists an integer $q(q\ge 1)$ satisfying ${\left({u}_{{a}_{ij}^{{{\prime}}\left(t\right)}}^{+}\right)}^{q}+{\left({v}_{{a}_{ij}^{{{\prime}}\left(t\right)}}^{+}\right)}^{q}\le 1$. ${a}_{ij}^{{{\prime}}\left(t\right)}$ that represents the judgment value of the t-th expert on the *j*-th attribute in alternative *i*-th. ${A}^{{{\prime}}\left(t\right)}$ is defined in Eq. (23):

23 $${A}^{{{\prime}}\left(t\right)}=\left(\begin{array}{ccc}\left(\left[{u}_{{a}_{11}^{{{\prime}}\left(t\right)}}^{-},{u}_{{a}_{11}^{{{\prime}}\left(t\right)}}^{+}\right],\left[{v}_{{a}_{11}^{{{\prime}}\left(t\right)}}^{-},{v}_{{a}_{11}^{{{\prime}}\left(t\right)}}^{+}\right]\right)& \cdots & \left(\left[{u}_{{a}_{1n}^{{{\prime}}\left(t\right)}}^{-},{u}_{{a}_{1n}^{{{\prime}}\left(t\right)}}^{+}\right],\left[{v}_{{a}_{1n}^{{{\prime}}\left(t\right)}}^{-},{v}_{{a}_{1n}^{{{\prime}}\left(t\right)}}^{+}\right]\right)\\ \vdots & \ddots & \vdots \\ \left(\left[{u}_{{a}_{m1}^{{{\prime}}\left(t\right)}}^{-},{u}_{{a}_{m1}^{{{\prime}}\left(t\right)}}^{+}\right],\left[{v}_{{a}_{m1}^{{{\prime}}\left(t\right)}}^{-},{v}_{{a}_{m1}^{{{\prime}}\left(t\right)}}^{+}\right]\right)& \cdots & \left(\left[{u}_{{a}_{mn}^{{{\prime}}\left(t\right)}}^{-},{u}_{{a}_{mn}^{{{\prime}}\left(t\right)}}^{+}\right],\left[{v}_{{a}_{mn}^{{{\prime}}\left(t\right)}}^{-},{v}_{{a}_{mn}^{{{\prime}}\left(t\right)}}^{+}\right]\right)\end{array}\right).$$

Before decision-making, experts are allowed to carry out a pre-judgment on the priority of the alternatives. If experts give preference $\left(g,l\right)$ for any pair (${x}_{g}$, ${x}_{l}$) of alternatives, which means that the expert prefers the alternative ${x}_{g}$. The set of all preference pairs, $P=\left\{\left(g,l\right)\right\}$, $1\le g\le m$, $1\le l\le m,g\ne l$, is given by experts in advance.

The indices of different performances need to be standardized. The ${\Omega }_{1}$ presents a set of benefit indices, and the ${\Omega }_{2}$ presents a set of cost indices. Equations (24) and (25) normalize the decision matrix ${A}^{{{\prime}}\left(t\right)}$ to the standard matrix ${A}^{\left(t\right)}$, where Eq. (25) is the complement operation of IVq-ROFN:

24 $${a}_{ij}^{(t)}=\left\{\begin{array}{l} {a}_{ij}^{{{\prime}}(t)} ,{a}_{ij}^{{{\prime}}(t)}\in {\Omega }_{1}\\ ({a}_{ij}^{{{\prime}}\left(t\right)}{)}^{c} ,{a}_{ij}^{{{\prime}}(t)}\in {\Omega }_{2}\end{array}\right. i={1,2},\cdots ,m, j={1,2},\cdots ,n$$

25 $$({a}_{ij}^{{{\prime}}(t)}{)}^{c}=\left([{v}_{{a}_{ij}^{{{\prime}}(t)}}^{-},{v}_{{a}_{ij}^{{{\prime}}(t)}}^{+}],[{u}_{{a}_{ij}^{{{\prime}}(t)}}^{-},{u}_{{a}_{ij}^{{{\prime}}(t)}}^{+}]\right)$$

### Deriving attribute weights

The LINMAP model has advantages in obtaining attribute weight. First, the LINMAP is simple, clear, and easy to implement. Second, LINMAP does not need attribute weights which can be solved by the linear programming model, and LINMAP can reflect the preferences and experience of the experts. Third, the linear programming model reflects the overall characteristics of the results. Therefore, we select LINMAP to derive attribute weights.

The classical LINMAP is based on pairwise comparisons of alternatives that are given by the DMs [59, 80]. The linear programming model is constructed to get attribute weights by minimizing the deviation of the total inconsistency index and the total consistency index. The LINMAP model includes primarily the following steps: (1) the alternative preference pairs are given by experts in advance, (2) the linear programming model is constructed according to the minimization of deviation of the total inconsistency index and the total consistency index, and (3) the attribute weights are obtained by solving linear programming model. Inspired by Zhang [80], we designed a method to derive attribute weights using the LINMAP model based on the similarity of IVq-ROFNs. First, the similarity between the alternatives and the positive ideal point is calculated, and the weighted similarity of alternatives is constructed according to the preference pair of alternatives given by experts in advance. Second, the linear programming model is constructed by minimizing the deviation of the inconsistent and consistent weighted similarity according to the preference pair of alternatives. The attribute weights of different experts are obtained by solving the linear programming model. Finally, the attribute weight matrix of all decision matrices is obtained. The LINMAP model solves the attribute weights as in the following steps.

1. Determining the preference set of alternatives $${\varvec{P}}=\left\{\left({\varvec{g}},{\varvec{l}}\right)\right\}$$.
2. Calculating the positive ideal point.
  Get the positive ideal point $${{a}_{j}^{*}}^{(t)}$$ of *j*-th column in the matrix $${A}^{\left(t\right)}$$ as shown Eq. (26):
  26 $${a}_{j}^{*(t)}=\left(\left[\underset{1\le i\le m}{\text{max}}\left({u}_{{a}_{ij}^{(t)}}^{-}\right), \underset{1\le i\le m}{\text{max}}\left({u}_{{a}_{ij}^{(t)}}^{+}\right)\right], \qquad \left[\underset{1\le i\le m}{\text{min}}\left({v}_{{a}_{ij}^{(t)}}^{-}\right),\underset{1\le i\le m}{\text{min}}\left({v}_{{a}_{ij}^{(t)}}^{+}\right)\right]\right)$$
3. Calculate the similarity of the alternatives to the ideal point.
  Use Eq. (9) to calculate the similarity $${S}_{g}({a}_{gj}^{(t)},{{a}_{j}^{*}}^{(t)})$$, $${S}_{l}({a}_{lj}^{(t)},{{a}_{j}^{*}}^{(t)})$$, for $$j=1, 2, \dots , n$$:
  27 $${S}_{g}({a}_{gj}^{(t)},{{a}_{j}^{*}}^{(t)})=\frac{\left({\left({u}_{{a}_{gj}^{(t)}}^{-}\right)}^{q}\wedge {\left({u}_{{{a}_{j}^{*}}^{(t)}}^{-}\right)}^{q}\right)+\left({\left({v}_{{a}_{gj}^{(t)}}^{-}\right)}^{q}\wedge {\left({v}_{{{a}_{j}^{*}}^{(t)}}^{-}\right)}^{q}\right)+\left({\left({u}_{{a}_{gj}^{(t)}}^{+}\right)}^{q}\wedge {\left({u}_{{{a}_{j}^{*}}^{(t)}}^{+}\right)}^{q}\right)+\left({\left({v}_{{a}_{gj}^{(t)}}^{+}\right)}^{q}\wedge {\left({v}_{{{a}_{j}^{*}}^{(t)}}^{+}\right)}^{q}\right)}{\left({\left({u}_{{a}_{gj}^{(t)}}^{-}\right)}^{q}\vee {\left({u}_{{{a}_{j}^{*}}^{(t)}}^{-}\right)}^{q}\right)+\left({\left({v}_{{a}_{gj}^{(t)}}^{-}\right)}^{q}\vee {\left({v}_{{{a}_{j}^{*}}^{(t)}}^{-}\right)}^{q}\right)+\left({\left({u}_{{a}_{gj}^{(t)}}^{+}\right)}^{q}\vee {\left({u}_{{{a}_{j}^{*}}^{(t)}}^{+}\right)}^{q}\right)+\left({\left({v}_{{a}_{gj}^{(t)}}^{+}\right)}^{q}\vee {\left({v}_{{{a}_{j}^{*}}^{(t)}}^{+}\right)}^{q}\right)}$$
  28 $${S}_{l}\left({a}_{lj}^{\left(t\right)},{{a}_{j}^{*}}^{\left(t\right)}\right)=\frac{\left({\left({u}_{{a}_{lj}^{\left(t\right)}}^{-}\right)}^{q}\wedge {\left({u}_{{{a}_{j}^{*}}^{\left(t\right)}}^{-}\right)}^{q}\right)+\left({\left({v}_{{a}_{lj}^{\left(t\right)}}^{-}\right)}^{q}\wedge {\left({v}_{{{a}_{j}^{*}}^{\left(t\right)}}^{-}\right)}^{q}\right)+\left({\left({u}_{{a}_{lj}^{\left(t\right)}}^{+}\right)}^{q}\wedge {\left({u}_{{{a}_{j}^{*}}^{\left(t\right)}}^{+}\right)}^{q}\right)+\left({\left({v}_{{a}_{lj}^{\left(t\right)}}^{+}\right)}^{q}\wedge {\left({v}_{{{a}_{j}^{*}}^{\left(t\right)}}^{+}\right)}^{q}\right)}{\left({\left({u}_{{a}_{lj}^{\left(t\right)}}^{-}\right)}^{q}\vee {\left({u}_{{{a}_{j}^{*}}^{\left(t\right)}}^{-}\right)}^{q}\right)+\left({\left({v}_{{a}_{lj}^{\left(t\right)}}^{-}\right)}^{q}\vee {\left({v}_{{{a}_{j}^{*}}^{\left(t\right)}}^{-}\right)}^{q}\right)+\left({\left({u}_{{a}_{lj}^{\left(t\right)}}^{+}\right)}^{q}\vee {\left({u}_{{{a}_{j}^{*}}^{\left(t\right)}}^{+}\right)}^{q}\right)+\left({\left({v}_{{a}_{lj}^{\left(t\right)}}^{+}\right)}^{q}\vee {\left({v}_{{{a}_{j}^{*}}^{\left(t\right)}}^{+}\right)}^{q}\right)}.$$
4. Calculate the weighted similarity of alternatives.
  Suppose the attribute weights are $${w}^{(t)}=({w}_{1}^{(t)},{w}_{2}^{(t)},\dots ,{w}_{n}^{(t)})$$. According to the preference pair set $$P=\left\{\left(g,l\right)\right\}$$, the weighted average $$Eq_{g}^{(t)}$$ and $$Eq_{l}^{(t)}$$ of $${w}_{j}^{(t)}$$ are calculated, which are as shown in Eqs. (29) and (30):
  29 $$ Eq_{g}^{(t)} =  \sum \limits_{j = 1}^{n} w_{j}^{(t)} \times S_{g} (a_{gj}^{(t)} ,a_{j}^{* (t)} ),\;j = 1,2, \ldots ,n $$
  30 $$ Eq_{l}^{(t)} =  \sum \limits_{j = 1}^{n} w_{j}^{(t)} \times S_{l} (a_{lj}^{(t)} ,a_{j}^{* (t)} ),\;j = 1,2, \ldots ,n $$
5. Construct a linear programming model.
  For a pair of preference $$(g,l)$$ in the set $$P$$, if $$Eq_{g}^{(t)}\le Eq_{l}^{(t)}$$, it means that the alternative $${x}_{g}$$ is closer to the ideal point than $${x}_{l}$$, and the weighted similarity is consistent with the preference of the expert. On the contrary, if $$Eq_{g}^{(t)}>Eq_{l}^{(t)}$$, it means that the weighted similarity is inconsistent with the preference of the experts. The actual alternative goal is to require the weighted similarity to be consistent with the preference of the experts. For this reason, the goal can be transformed into a linear programming problem as shown in Eq. (31):
  31 $$ \begin{gathered} {\text{min}} \sum \limits_{{\left( {g,l} \right) \in P}} \theta_{gl}^{(t)} \hfill \\ {\text{S.T.}}\left\{ {\begin{array}{*{20}l} {Eq_{l}^{(t)} - Eq_{g}^{(t)} + \theta_{gl}^{(t)} \ge 0} \hfill \\ { \sum \limits_{{\left( {g,l} \right) \in P}} \left( {Eq_{l}^{(t)} - Eq_{g}^{(t)} } \right) = B^{(t)} } \hfill \\ {\sum\limits_{j = 1}^{n} {w_{j}^{(t)} = 1} } \hfill \\ {w_{j}^{(t)} \ge 0} \hfill \\ {\theta_{gl}^{(t)} \ge 0} \hfill \\ \end{array} } \right. \hfill \\ \end{gathered} $$
  In Eq. (31), $${\theta }_{gl}^{(t)}$$ represents the deviation between alternatives and the weighted similarity of $$\left(g,l\right)$$ and $${\theta }_{gl}^{(t)}\ge 0$$. The $${\theta }_{gl}^{(t)}$$ of different alternative pairs does not affect each other. The sum of $${\theta }_{gl}^{(t)}$$ is $${B}^{(t)}$$ corresponding to all pairs of the alternatives in the order set $$P$$ of the preferred alternative.
6. Solve the attribute weights of the decision matrix.
  From Eq. (31), the attribute weight $${w}^{(t)}$$ of the decision matrix of the *t*-th expert can be obtained as
  32 $${w}^{\left(t\right)}=\left({w}_{1}^{\left(t\right)},{w}_{2}^{\left(t\right)},\ldots,{w}_{n}^{\left(t\right)}\right).$$
7. Get the attribute weight matrix of all decision matrices.
  According to Eq. (32), the attribute weights of all decision matrices are expressed as a matrix with $$k$$ rows and $$n$$ columns in Eq. (33):
  33 $$W=\left[\begin{array}{l}{w}^{(1)}\\
{w}^{(2)}\\ \vdots \\
{w}^{\left(k\right)}\end{array}\right]=\left[\begin{array}{c@{\quad}c@{\quad}c@{\quad}c}{w}_{1}^{\left(1\right)}&
{w}_{2}^{\left(1\right)}& \cdots & {w}_{n}^{\left(1\right)}\\
{w}_{1}^{\left(2\right)}& {w}_{2}^{\left(2\right)}& \cdots &
{w}_{n}^{\left(2\right)}\\ \vdots & \vdots & \ddots & \vdots \\
{w}_{1}^{\left(k\right)}& {w}_{2}^{\left(k\right)}& \cdots &
{w}_{n}^{\left(k\right)}\end{array}\right].$$
  Using the LINMAP model, the attribute weight $${w}^{\left(t\right)}$$ of a single expert decision matrix $${A}^{\left(t\right)}$$ can be calculated separately. Elements at corresponding positions in different matrices can be aggregated by expert weights, which better reflect the way that the sum is calculated.

### Deriving expert weights

The decision matrices ${A}^{\left(1\right)}$ and ${A}^{\left(2\right)}$ produced by the two experts are shown in Tables 3 and 4. It can be seen from Tables 3 and 4 that alternatives ${x}_{1}$ and ${x}_{2}$ given by the experts ${D}^{\left(1\right)}$ and ${D}^{\left(2\right)}$ have obvious differences. The weights of ${D}^{\left(1\right)}$ and ${D}^{\left(2\right)}$ have been set at 0.5 as suggested by Yue [75, 76] which cannot be distinguished. It is obviously inconsistent with the difference of the alternative judgment value given by experts ${D}^{\left(1\right)}$ and ${D}^{\left(2\right)}$, so it cannot reflect the objective actual situation. In real life, experts’ judgments on different system options will be affected by the external environment, such as their psychological state, alternative expression, and surrounding environment. Thus, the experts are given different weights.

**Table 3:** Decision matrix ${A}^{\left(1\right)}$

	$C1$	$C2$	$C3$
${x}_{1}$	$([{0.29,0.38}],[{0.12,0.63}])$	$([{0.41,0.74}],[{0.11,0.57}])$	$([{0.36,0.47}],[{0.56,0.79}])$
${x}_{2}$	$([{0.16,0.31}],[{0.01,0.51}])$	$([{0.41,0.64}],[{0.46,0.73}])$	$([{0.11,0.72}],[{0.12,0.46}])$
${x}_{3}$	$([{0.12,0.23}],[{0.03,0.41}])$	$([{0.30,0.33}],[{0.23,0.59}])$	$([{0.14,0.52}],[{0.27,0.53}])$
${x}_{4}$	$([{0.25,0.54}],[{0.51,0.62}])$	$([{0.05,0.49}],[{0.26,0.73}])$	$([{0.72,0.90}],[{0.22,0.42}])$

**Table 4:** Decision matrix ${A}^{\left(2\right)}$

	$C1$	$C2$	$C3$
${x}_{1}$	$([{0.12,0.23}],[{0.03,0.41}])$	$([{0.3,0.33}],[{0.23,0.59}])$	$([{0.14,0.52}],[{0.27,0.53}])$
${x}_{2}$	$([{0.25,0.54}],[{0.51,0.62}])$	$([{0.05,0.49}],[{0.26,0.73}])$	$([{0.72,0.90}],[{0.22,0.42}])$
${x}_{3}$	$([{0.29,0.38}],[{0.12,0.63}])$	$([{0.41,0.74}],[{0.11,0.57}])$	$([{0.36,0.47}],[{0.56,0.79}])$
${x}_{4}$	$([{0.16,0.31}],[{0.01,0.51}])$	$([{0.41,0.64}],[{0.46,0.73}])$	$([{0.11,0.72}],[{0.12,0.46}])$

Inspired by [75], the LINMAP model is used to derive expert weights that each alternative has itself expert weights. First, the IVq-ROFWFAWA operator is adopted to aggregate expert decision matrices according to different alternatives, and the fusion matrix is obtained. Second, the similarity, which is calculated each element of the fusion matrix between ideal point, is used to derive expert weights for each alternative. In this way, the expert weight matrix of different alternatives is obtained. For $k$ experts and $m$ alternatives, an $m\times k$ expert weight matrix can be obtained by the following steps.

1. Aggregate different expert decision matrices according to different alternatives.
  According to the attribute weights of the decision matrix, the IVq-ROFWFAWA operator is used to aggregate the rows of each decision matrix as shown Eq. (34). For *t*-th expert, the aggregation result of ${{\overline{D}}}^{(t)}$ has *m* IVq-ROFNs which can be obtained as Eq. (35). For all experts, the aggregation result is a fusion matrix ${\overline{D}}$ with *k* rows and *m* columns defined as Eq. (36):
  34 $$ d_{i}^{{\left( t \right)}}  = {\text{IVq - ROFWFAWA}}\left( {a_{{ij}}^{{\left( t \right)}} ,a_{{ij}}^{{\left( t \right)}} , \ldots ,a_{{ij}}^{{\left( t \right)}} } \right) = \mathop { \otimes _{F} }\limits_{{j = 1}}^{n} w_{j}^{{\left( t \right)}} a_{{ij}}^{{\left( t \right)}} , $$
  35 $${{\overline{D}}}^{\left(t\right)}=\left[{d}_{1}^{\left(t\right)},{d}_{2}^{\left(t\right)},\ldots,{d}_{m}^{\left(t\right)}\right],$$
  36 $${\overline{D}}=\left(\begin{array}{l}{{\overline{D}}}^{\left(1\right)}\\ {{\overline{D}}}^{\left(2\right)}\\ \vdots \\ {{\overline{D}}}^{\left(k\right)}\end{array}\right)=\left(\begin{array}{c@{\quad}c@{\quad}c@{\quad}c}{d}_{1}^{\left(1\right)}& {d}_{2}^{\left(1\right)}& \cdots & {d}_{m}^{\left(1\right)}\\ {d}_{1}^{\left(2\right)}& {d}_{2}^{\left(2\right)}& \cdots & {d}_{m}^{\left(2\right)}\\ \vdots & \vdots & \ddots & \vdots \\ {d}_{1}^{\left(k\right)}& {d}_{2}^{\left(k\right)}& \cdots & {d}_{m}^{\left(k\right)}\end{array}\right).$$
2. Obtain the ideal point of the fusion matrix ${\overline{D}}$.
  The positive ideal point ${d}_{j}^{+*}$ and the negative ideal point ${d}_{j}^{-*}$ of *j*-th column in the matrix ${\overline{D}}$ are calculated in Eqs. (37) and (38):
  37 $${d}_{j}^{+*}=\left(\left[\underset{1\le t\le k}{\text{max}}\left({u}_{{d}_{j}^{\left(t\right)}}^{-}\right),\underset{1\le t\le k}{\text{max}}\left({u}_{{d}_{j}^{\left(t\right)}}^{+}\right)\right],\left[\underset{1\le t\le k}{\text{min}}\left({v}_{{d}_{j}^{\left(t\right)}}^{-}\right),\underset{1\le t\le k}{\text{min}}\left({v}_{{d}_{j}^{\left(t\right)}}^{+}\right)\right]\right),$$
  38 $${d}_{j}^{-*}=\left(\left[\underset{1\le t\le k}{\text{min}}\left({u}_{{d}_{j}^{\left(t\right)}}^{-}\right),\underset{1\le t\le k}{\text{min}}\left({u}_{{d}_{j}^{\left(t\right)}}^{+}\right)\right],\left[\underset{1\le t\le k}{\text{max}}\left({v}_{{d}_{j}^{\left(t\right)}}^{-}\right),\underset{1\le t\le k}{\text{max}}\left({v}_{{d}_{j}^{\left(t\right)}}^{+}\right)\right]\right).$$
3. Calculate the similarity of each element of ${\overline{D}}$ to the ideal point.
  In matrix ${\overline{D}}$, the positive ideal similarity ${s}_{j}^{(+t)}$ and the negative ideal similarity ${s}_{j}^{(-t)}$ between each element ${d}_{j}^{\left(t\right)}$ and ${d}_{j}^{*}$ are calculated in Eqs. (39) and (40). The similarity matrices ${\text{SIM}}^{+}$ and ${{\text{SIM}}}^{-}$ of all ideal points are given in Eqs. (41) and (42):
  39 $${{s}_{j}}^{\left(+t\right)}=S\left({d}_{j}^{\left(t\right)},{d}_{j}^{+*}\right)=\frac{\left({\left({u}_{{d}_{j}^{\left(t\right)}}^{-}\right)}^{q}\wedge {\left({u}_{{d}_{j}^{+*}}^{-}\right)}^{q}\right)+\left({\left({v}_{{d}_{j}^{\left(t\right)}}^{-}\right)}^{q}\wedge {\left({v}_{{d}_{j}^{+*}}^{-}\right)}^{q}\right)+\left({\left({u}_{{d}_{j}^{\left(t\right)}}^{+}\right)}^{q}\wedge {\left({u}_{{d}_{j}^{+*}}^{+}\right)}^{q}\right)+\left({\left({v}_{{d}_{j}^{\left(t\right)}}^{+}\right)}^{q}\wedge {\left({v}_{{d}_{j}^{+*}}^{+}\right)}^{q}\right)}{\left({\left({u}_{{d}_{j}^{\left(t\right)}}^{-}\right)}^{q}\vee {\left({u}_{{d}_{j}^{+*}}^{-}\right)}^{q}\right)+\left({\left({v}_{{d}_{j}^{\left(t\right)}}^{-}\right)}^{q}\vee {\left({v}_{{d}_{j}^{+*}}^{-}\right)}^{q}\right)+\left({\left({u}_{{d}_{j}^{\left(t\right)}}^{+}\right)}^{q}\vee {\left({u}_{{d}_{j}^{+*}}^{+}\right)}^{q}\right)+\left({\left({v}_{{d}_{j}^{\left(t\right)}}^{+}\right)}^{q}\vee {\left({v}_{{d}_{j}^{+*}}^{+}\right)}^{q}\right)},$$
  40 $${{s}_{j}}^{\left(-t\right)}=S\left({d}_{j}^{\left(t\right)},{d}_{j}^{-*}\right)=\frac{\left({\left({u}_{{d}_{j}^{\left(t\right)}}^{-}\right)}^{q}\wedge {\left({u}_{{d}_{j}^{-*}}^{-}\right)}^{q}\right)+\left({\left({v}_{{d}_{j}^{\left(t\right)}}^{-}\right)}^{q}\wedge {\left({v}_{{d}_{j}^{-*}}^{-}\right)}^{q}\right)+\left({\left({u}_{{d}_{j}^{\left(t\right)}}^{+}\right)}^{q}\wedge {\left({u}_{{d}_{j}^{-*}}^{+}\right)}^{q}\right)+\left({\left({v}_{{d}_{j}^{\left(t\right)}}^{+}\right)}^{q}\wedge {\left({v}_{{d}_{j}^{-*}}^{+}\right)}^{q}\right)}{\left({\left({u}_{{d}_{j}^{\left(t\right)}}^{-}\right)}^{q}\vee {\left({u}_{{d}_{j}^{-*}}^{-}\right)}^{q}\right)+\left({\left({v}_{{d}_{j}^{\left(t\right)}}^{-}\right)}^{q}\vee {\left({v}_{{d}_{j}^{-*}}^{-}\right)}^{q}\right)+\left({\left({u}_{{d}_{j}^{\left(t\right)}}^{+}\right)}^{q}\vee {\left({u}_{{d}_{j}^{-*}}^{+}\right)}^{q}\right)+\left({\left({v}_{{d}_{j}^{\left(t\right)}}^{+}\right)}^{q}\vee {\left({v}_{{d}_{j}^{-*}}^{+}\right)}^{q}\right)},$$
  41 $${\text{SIM}}^{+}=\left[\begin{array}{l}{{\text{SIM}}}^{(+1)}\\ {{\text{SIM}}}^{(+2)}\\ \vdots \\ {{\text{SIM}}}^{(+{k})}\end{array}\right]=\left[\begin{array}{c@{\quad}c@{\quad}c@{\quad}c}{s}_{1}^{\left(+1\right)}& {s}_{2}^{\left(+1\right)}& \cdots & {s}_{m}^{\left(+1\right)}\\ {s}_{1}^{\left(+2\right)}& {s}_{2}^{\left(+2\right)}& \cdots & {s}_{m}^{\left(+2\right)}\\ \vdots & \vdots & \ddots & \vdots \\ {s}_{1}^{\left(+k\right)}& {s}_{2}^{\left(+k\right)}& \cdots & {s}_{m}^{\left(+k\right)}\end{array}\right],$$
  42 $${{\text{SIM}}}^{-}=\left[\begin{array}{l}{{\text{SIM}}}^{(-1)}\\ {{\text{SIM}}}^{(-2)}\\ \vdots \\ {{\text{SIM}}}^{(-k)}\end{array}\right]=\left[\begin{array}{c@{\quad}c@{\quad}c@{\quad}c}{s}_{1}^{\left(-1\right)}& {s}_{2}^{\left(-1\right)}& \cdots & {s}_{m}^{\left(-1\right)}\\ {s}_{1}^{\left(-2\right)}& {s}_{2}^{\left(-2\right)}& \cdots & {s}_{m}^{\left(-2\right)}\\ \vdots & \vdots & \ddots & \vdots \\ {s}_{1}^{\left(-k\right)}& {s}_{2}^{\left(-k\right)}& \cdots & {s}_{m}^{\left(-k\right)}\end{array}\right].$$
4. Get expert weight matrix.
  For the *i*-th alternative of the *t*-th expert, the expert weight of ${\lambda }_{i}^{\left(t\right)}$ is calculated in Eq. (43). The expert weight matrix can be determined by Eq. (44):
  43 $$ \lambda_{i}^{\left( t \right)} = \frac{{\frac{{s_{i}^{{^{{\left( { - t} \right)}} }} }}{{s_{i}^{{^{{\left( { - t} \right)}} }} + s_{i}^{{^{{\left( { + t} \right)}} }} }}}}{{ \sum \nolimits_{t = 1}^{k} \frac{{s_{i}^{{^{{\left( { - t} \right)}} }} }}{{s_{i}^{{^{{\left( { - t} \right)}} }} + s_{i}^{{^{{\left( { + t} \right)}} }} }}}}, $$
  44 $$\lambda =\left[\begin{array}{l}{\lambda }^{(1)}\\ {\lambda }^{(2)}\\ \vdots \\ {\lambda }^{(k)}\end{array}\right]=\left[\begin{array}{c@{\quad}c@{\quad}c@{\quad}c}{\lambda }_{1}^{\left(1\right)}& {\lambda }_{2}^{\left(1\right)}& \cdots & {\lambda }_{m}^{\left(1\right)}\\ {\lambda }_{1}^{\left(2\right)}& {\lambda }_{2}^{\left(2\right)}& \cdots & {\lambda }_{m}^{\left(2\right)}\\ \vdots & \vdots & \ddots & \vdots \\ {\lambda }_{1}^{\left(k\right)}& {\lambda }_{2}^{\left(k\right)}& \cdots & {\lambda }_{m}^{\left(k\right)}\end{array}\right].$$
  According to the decision matrices ${A}^{\left(1\right)}$ and ${A}^{\left(2\right)}$ given by the experts ${D}^{\left(1\right)}$ and ${D}^{\left(2\right)}$, the expert weights which are presented by a matrix are derived by our proposed method, the results are shown in Table 5.

**Table 5:** Expert weights results of the proposed method

	${D}_{1}$	${D}_{2}$
${w}_{1}^{(t)}$	0.5299	0.4701
${w}_{2}^{(t)}$	0.4562	0.5438
${w}_{3}^{(t)}$	0.4294	0.5706
${w}_{4}^{(t)}$	0.5757	0.4243

It can be seen from Table 5 that expert weights are different, compared with Yue [75, 76], the proposed method is more adaptable. Our method, thus, reflects the real situation of the experts more objectively.

### MAGDM method based on ARAS

**Fig. 2:**
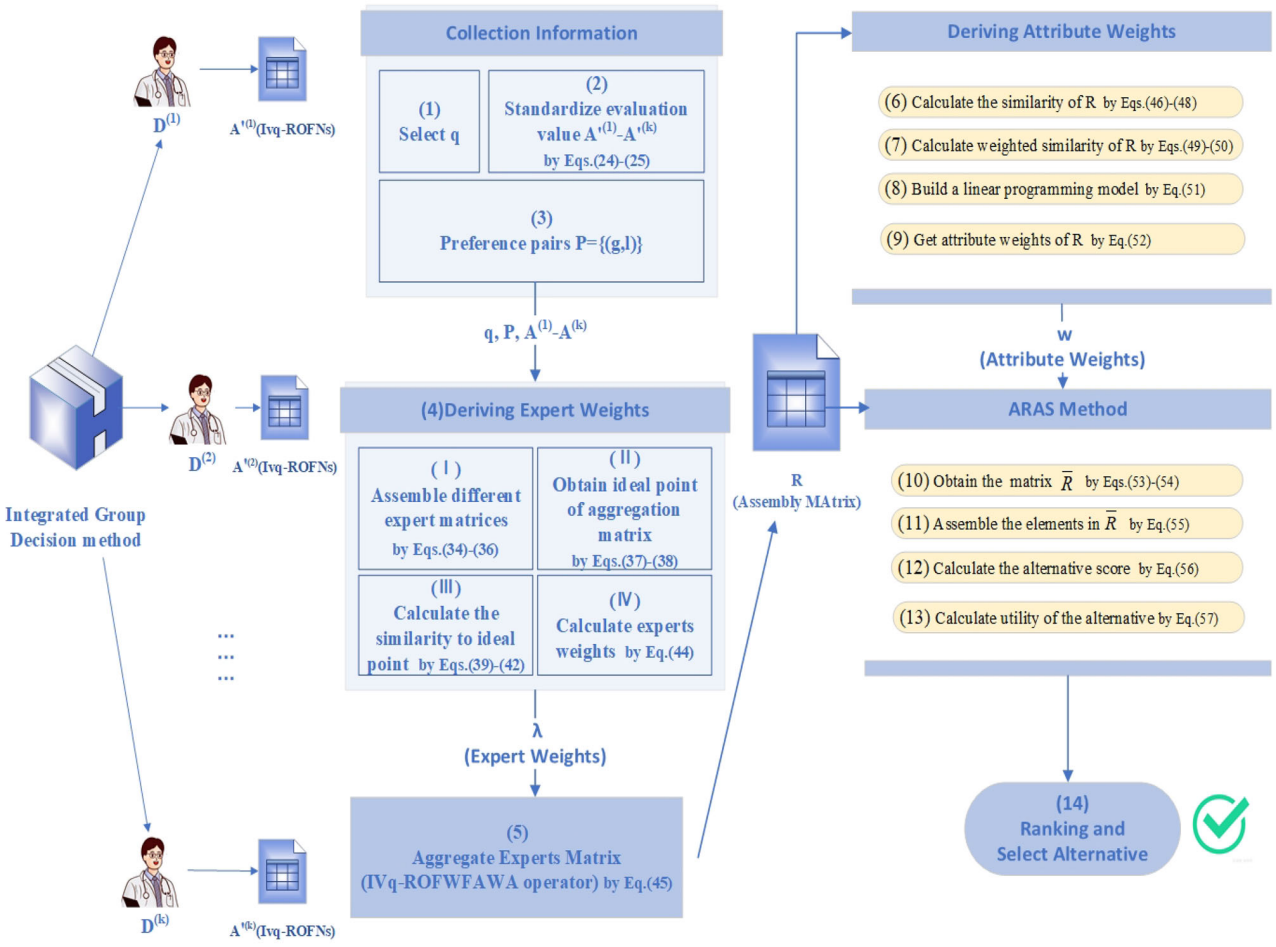
MAGDM flow based on the ARAS method

In this subsection, the proposed MAGDM method includes two information aggregating processes. First, the proposed IVq-ROFWFAFWA operator is used to aggregate the decision matrix of each expert that obtains the aggregation matrix *R*. Second, the ARAS method is used to select the optimal alternative from matrix *R*. Figure 2 shows the process for developing the MAGDM method. In addition, its steps of implementation are following.

1. Determine the appropriate *q* value.
  According to the decision matrix provided by the experts, the traversal method is used to compute the smallest positive integer *q* which makes all elements satisfy $${\left({u}_{{a}_{ij}^{\left(t\right)}}^{+}\right)}^{q}+{\left({v}_{{a}_{ij}^{\left(t\right)}}^{+}\right)}^{q}\le 1, q\ge 1$$.
2. Standardize.
  In the application scenario, if both cost-type and benefit-type attributes are included, cost-type attributes will be uniformly transformed into benefit-type attributes. The standardizing process is given by Eqs. (24) and (25).
3. Determine the preference pairs set $$P=\left\{\left(g,l\right)\right\}$$.
  The pre-evaluation, which the experts have carried out in advance, determines the alternative preference pairs set *P*.
4. Derive expert weight matrix $$\lambda $$.
  First, the LINMAP model is used to solve the attribute weight vector $${w}^{(t)}$$ of each decision matrix and to obtain the attribute weight matrix *W*. The solving steps are shown in Eqs. (26)–(33). The *W* and the IVq-ROFWFAWA operator are used to aggregate the different alternatives and obtain the aggregation vector $${{\overline{D}}}^{(t)}$$ of each expert. $${{\overline{D}}}^{(t)}$$ is fused to a matrix $${\overline{D}}$$. The solving processes are given in Eqs. (34)–(36). The similarity is then used to compute the weights of the different alternatives. For each alternative, the weights of each expert are obtained. After combination, an expert weight matrix $$\lambda $$ is obtained. The solving processes are conducted in Eqs. (37)–(44).
5. Get the aggregation matrix $$R$$.
  According to the expert weight matrix λ solved in step (4), the IVq-ROFWFAWA operator is used to aggregate elements at the same position of $${A}^{\left(t\right)}$$ (*t* = 1, 2, …, *k*). The aggregation matrix $$R={\left({r}_{ij}\right)}_{m\times n}$$ is obtained by Eq. (45) for $$i={1,2},\dots ,m;j={1,2},\dots ,n$$:
  45 $$ r_{{ij}}  = {\text{IVq-ROFWFAWA}}\left( {a_{{ij}}^{{\left( 1 \right)}} ,a_{{ij}}^{{\left( 2 \right)}} , \ldots ,a_{{ij}}^{{\left( k \right)}} } \right) = \mathop { \otimes _{F} }\limits_{{t = 1}}^{k} \lambda _{j}^{{\left( t \right)}} a_{{ij}}^{{\left( t \right)}} . $$
6. Calculate the similarity of the matrix *R* to the ideal point.
  The positive ideal point $${r}_{j}^{*}$$ of each column of the matrix *R* can be found by Eq. (46). The similarity $${SR}_{g}({r}_{gj},{r}_{j}^{*})$$ and $${SR}_{l}({r}_{lj},{r}_{j}^{*})$$ are then calculated by Eqs. (47) and Eq. (48):
  46 $$\begin{aligned}{r}_{j}^{*}&=\left(\left[\underset{1\le i\le
m}{\text{max}}\left({u}_{{r}_{ij}}^{-}\right),\underset{1\le i\le
m}{\text{max}}\left({u}_{{r}_{ij}}^{+}\right)\right],\right.\\
&\left.\left[\underset{1\le
i\le m}{\text{min}}\left({v}_{{r}_{ij}}^{-}\right),\underset{1\le
i\le
m}{\text{min}}\left({v}_{{r}_{ij}}^{+}\right)\right]\right),\end{aligned}$$
  47 $$S{R}_{g}\left({r}_{gj},{r}_{j}^{*}\right)=\frac{\left({\left({u}_{{r}_{gj}}^{-}\right)}^{q}\wedge {\left({u}_{{r}_{j}^{*}}^{-}\right)}^{q}\right)+\left({\left({v}_{{r}_{gj}}^{-}\right)}^{q}\wedge {\left({v}_{{r}_{j}^{*}}^{-}\right)}^{q}\right)+\left({\left({u}_{{r}_{gj}}^{+}\right)}^{q}\wedge {\left({u}_{{r}_{j}^{*}}^{+}\right)}^{q}\right)+\left({\left({v}_{{r}_{gj}}^{+}\right)}^{q}\wedge {\left({v}_{{r}_{j}^{*}}^{+}\right)}^{q}\right)}{\left({\left({u}_{{r}_{gj}}^{-}\right)}^{q}\vee {\left({u}_{{r}_{j}^{*}}^{-}\right)}^{q}\right)+\left({\left({v}_{{r}_{gj}}^{-}\right)}^{q}\vee {\left({v}_{{r}_{j}^{*}}^{-}\right)}^{q}\right)+\left({\left({u}_{{r}_{gj}}^{+}\right)}^{q}\vee {\left({u}_{{r}_{j}^{*}}^{+}\right)}^{q}\right)+\left({\left({v}_{{r}_{gj}}^{+}\right)}^{q}\vee {\left({v}_{{r}_{j}^{*}}^{+}\right)}^{q}\right)},$$
  48 $$S{R}_{l}\left({r}_{lj},{r}_{j}^{*}\right)=\frac{\left({\left({u}_{{r}_{lj}}^{-}\right)}^{q}\wedge {\left({u}_{{r}_{j}^{*}}^{-}\right)}^{q}\right)+\left({\left({v}_{{r}_{lj}}^{-}\right)}^{q}\wedge {\left({v}_{{r}_{j}^{*}}^{-}\right)}^{q}\right)+\left({\left({u}_{{r}_{lj}}^{+}\right)}^{q}\wedge {\left({u}_{{r}_{j}^{*}}^{+}\right)}^{q}\right)+\left({\left({v}_{{r}_{lj}}^{+}\right)}^{q}\wedge {\left({v}_{{r}_{j}^{*}}^{+}\right)}^{q}\right)}{\left({\left({u}_{{r}_{lj}}^{-}\right)}^{q}\vee {\left({u}_{{r}_{j}^{*}}^{-}\right)}^{q}\right)+\left({\left({v}_{{r}_{lj}}^{-}\right)}^{q}\vee {\left({v}_{{r}_{j}^{*}}^{-}\right)}^{q}\right)+\left({\left({u}_{{r}_{lj}}^{+}\right)}^{q}\vee {\left({u}_{{r}_{j}^{*}}^{+}\right)}^{q}\right)+\left({\left({v}_{{r}_{lj}}^{+}\right)}^{q}\vee {\left({v}_{{r}_{j}^{*}}^{+}\right)}^{q}\right)}.$$
7. Calculate the weighted similarity of the matrix $$R$$.
  Let attribute weights of $$R$$ be $$w=({w}_{1},{w}_{2},\dots ,{w}_{n})$$. The weighted average values $${Eqr}_{g}\,{ {and}\, Eqr}_{l}$$ of $${SR}_{g}({r}_{gj},{r}_{j}^{*})$$ and $${SR}_{l}({r}_{lj},{r}_{j}^{*})$$ are calculated by Eqs. (49) and (50).
  49 $$ Eqr_{g} =  \sum \limits_{j = 1}^{n} w_{j} \times SR_{g} (r_{gj} ,r_{j}^{*} ),\;j = 1,2, \ldots ,n $$
  50 $$ Eqr_{l} =  \sum \limits_{j = 1}^{n} w_{j} \times SR_{l} (r_{lj} ,r_{j}^{*} ),\;j = 1,2, \ldots ,n $$
8. Construct a linear programming model.
  For a pair of alternatives $$(g,l)$$ in the set of preference pair $$P$$, if $$E{qr}_{g}\le E{qr}_{l}$$, it means that the alternative $${x}_{g}$$ is closer to the ideal point than $${x}_{l}$$, and the weighted similarity is consistent with the expert’s preference. On the contrary, if $$E{qr}_{g}>E{qr}_{l}$$, the weighted similarity will be inconsistent with the expert preference. The goal of the actual alternative should be that the weighted similarity and the preference of the expert are usually consistent. According to the idea of the LINMAP model, this goal can be transformed into the linear programming model:
  51 $$ \begin{gathered} {\text{min}} \sum \limits_{{\left( {g,l} \right) \in P}} \theta r_{gl} , \hfill \\ {\text{S.T.}}\left\{ {\begin{array}{*{20}l} {Eqr_{l} - Eqr_{g} + \theta r_{gl} \ge 0} \hfill \\ { \sum \limits_{{\left( {g,l} \right) \in P}} \left( {Eqr_{l} - Eqr_{g} } \right) = B} \hfill \\ {\sum\limits_{j = 1}^{n} {w_{j} = 1} } \hfill \\ {w_{j} \ge 0} \hfill \\ {\theta r_{gl} \ge 0} \hfill \\ \end{array} } \right.. \hfill \\ \end{gathered} $$
9. Get the attribute weights of *R*.
  The linear programming model of Eq. (51) will be solved. The attribute weight *w* of *R* can be obtained:
  52 $$w=\left({w}_{1},{w}_{2},\ldots,{w}_{n}\right).$$
  The ARAS method is used to select the best alternative. The main idea of the ARAS method is to select the best alternative based on multiple attributes and determine the final ranking of the alternatives by determining the utility of each one. The following steps use the ARAS idea to obtain the optimal alternative.
10. Obtain the optimal alternative $${\overline{R}}$$.
  Using the score function in Eq. (14), the element with the largest matrix *R* score for each column can be identified. The element with the largest score in the *j*-th column can be solved by Eq. (53), where $$S({r}_{ij})$$ represents the score of the element $${r}_{ij}$$. The elements with the highest scores in all columns then form a new alternative $${x}_{0}$$. This is then added to the 0th row of *R*, so that a new decision matrix $$\overline{R }={({\overline{r} }_{ij})}_{m\times n}$$ can be obtained by Eq. (54) for $$i={0,1},2,\dots ,m;j={1,2},\dots ,n$$:
  53 $${{\overline{r}}}_{0j}=\left(\left[{u}_{{r}_{ij}}^{-},{u}_{{r}_{ij}}^{+}\right],\left[{v}_{{r}_{ij}}^{-},{v}_{{r}_{ij}}^{+}\right]\right)\underset{i}{={\text{max}}}\left\{S\left({r}_{ij}\right)\right\},$$
  54 $${\overline{R}}=({\overline{r}}{)}_{m\times n}=\left(\begin{array}{ccc}\left(\left[{u}_{{{\overline{r}}}_{01}}^{-},{u}_{{{\overline{r}}}_{01}}^{+}\right],\left[{v}_{{{\overline{r}}}_{01}}^{-},{v}_{{{\overline{r}}}_{01}}^{+}\right]\right)& \cdots & \left(\left[{u}_{{{\overline{r}}}_{0n}}^{-},{u}_{{{\overline{r}}}_{0n}}^{+}\right],\left[{v}_{{{\overline{r}}}_{0n}}^{-},{v}_{{{\overline{r}}}_{0n}}^{+}\right]\right)\\ \vdots & \ddots & \vdots \\ \left(\left[{u}_{{{\overline{r}}}_{m1}}^{-},{u}_{{{\overline{r}}}_{m1}}^{+}\right],\left[{v}_{{{\overline{r}}}_{m1}}^{-},{v}_{{{\overline{r}}}_{m1}}^{+}\right]\right)& \cdots & \left(\left[{u}_{{{\overline{r}}}_{mn}}^{-},{u}_{{{\overline{r}}}_{mn}}^{+}\right],\left[{v}_{{{\overline{r}}}_{mn}}^{-},{v}_{{{\overline{r}}}_{mn}}^{+}\right]\right)\end{array}\right).$$
11. Aggregate the elements in $$\overline{R }$$.
  The IVq-ROFWFAWA operator is used to aggregate the elements $${\overline{r} }_{ij}$$ of each row of $$\overline{R }$$ by Eq. (55), and the aggregation value $$b{{\overline{r}}}_{i}$$ can be obtained:
  55 $$  b{\overline{r}}_{i}  = {\text{IVq-ROFWFAWA}}\left( {{\overline{r}}_{{i1}} ,{\overline{r}}_{{i2}} ,\ldots,{\overline{r}}_{{in}} } \right) = \mathop { \otimes _{F} }\limits_{{j = 1}}^{n} w_{j} {\overline{r}}_{{ij}} .  $$
12. Calculate alternative score.
  With the $$b{{\overline{r}}}_{i}$$ of each alternative solved in step (11), each alternative score $$s{{\overline{r}}}_{i}$$ is calculated by Eq. (56):
  56 $$s{{\overline{r}}}_{i}=\frac{1}{4}\left(\begin{array}{l}ln\left({u}_{b{{\overline{r}}}_{i}}^{-}+{u}_{b{{\overline{r}}}_{i}}^{+}+{v}_{b{{\overline{r}}}_{i}}^{-}+{v}_{b{{\overline{r}}}_{i}}^{+}+1\right)+2\times \left(\left({u}_{b{{\overline{r}}}_{i}}^{-}+{u}_{b{{\overline{r}}}_{i}}^{+}\right)-\left({v}_{b{{\overline{r}}}_{i}}^{-}+{v}_{b{{\overline{r}}}_{i}}^{+}\right)\right)\\ +\left(({u}_{b{{\overline{r}}}_{i}}^{+}-{u}_{b{{\overline{r}}}_{i}}^{-})+({v}_{b{{\overline{r}}}_{i}}^{+}-{v}_{b{{\overline{r}}}_{i}}^{-})\right)+\left(({u}_{b{{\overline{r}}}_{i}}^{+}-{u}_{b{{\overline{r}}}_{i}}^{-})-({v}_{b{{\overline{r}}}_{i}}^{+}-{v}_{b{{\overline{r}}}_{i}}^{-})\right)\\ -{Sign}\left({u}_{b{{\overline{r}}}_{i}}^{-}+{u}_{b{{\overline{r}}}_{i}}^{+}+{v}_{b{{\overline{r}}}_{i}}^{-}+{v}_{b{{\overline{r}}}_{i}}^{+}\right)\times ln3\end{array}\right).$$
13. Calculate the utility value of the alternative.
  Because the 0-th alternative is best, the utility of the alternative is equal to the score of the alternative divided by the score of the 0-th alternative:
  57 $${e}_{i}=\frac{s{{\overline{r}}}_{i}}{s{{\overline{r}}}_{0}}, i=1,\dots ,m.$$
14. Rank alternatives and select the optimal alternative.
  According to the utility $${e}_{i}$$ for each alternative obtained in step (13), the greater the effect is, the better the alternative is. The alternative with the largest utility, therefore, is the optimal alternative.

## Evaluation and analysis of the HFU system

The process for evaluating an HFU system is described in “Evaluating an HFU system”. A sensitivity analysis of the evaluation methods is conducted in “Sensitivity analysis of evaluation parameters”, and a comparison and more general analysis are carried out in “Comparative analysis”.

### Evaluating an HFU system

#### Expert evaluation

After a public bidding procedure, the optimal HFU system will be selected from the five HFU systems. Each HFU system was subjected to expert review, and was preliminarily evaluated by the hospital. The preference pairs set is obtained: $P=\left\{(\text{5,4}),(\text{5,1}),(\text{3,2}),(\text{1,2})\right\}$. Five experts were then invited to evaluate each system. In order to facilitate expert evaluation, a linguistic-graded evaluation scale was adopted. Inspired by Ilbahar et al. [25], the linguistic-graded scale included ten grades, with each of the linguistic terms corresponding to the ten IVq-ROFNs listed in Table 6. Five experts evaluated the five HFU systems according to their expertise and the indices given in Table 1. The evaluation matrices ${L}^{\left(1\right)},{L}^{\left(2\right)},{L}^{\left(3\right)},{L}^{\left(4\right)},{L}^{\left(5\right)}$ are listed in Tables 7, 8, 9, 10, and 11.

**Table 6:** Linguistic terms corresponding to IVq-ROFNs

Linguistic terms	IVq-ROFNs			
	${\mu }_{L}$	${\mu }_{U}$	${v}_{L}$	${v}_{U}$
Certainly low important (CLI)	0.05	0.05	0.90	0.95
Very low important (VLI)	0.10	0.20	0.80	0.90
Low important (LI)	0.20	0.35	0.65	0.80
Below average important (BAI)	0.35	0.45	0.55	0.65
Average important (AI)	0.45	0.55	0.45	0.55
Above average important (AAI)	0.55	0.65	0.35	0.45
High important (HI)	0.65	0.80	0.20	0.35
Very high important (VHI)	0.80	0.90	0.10	0.20
Certainly high important (CHI)	0.90	0.95	0.05	0.05
Exactly equal (EE)	0.1965	0.1965	0.1965	0.1965

**Table 7:** ${D}^{\left(1\right)}$ evaluation value ${L}^{\left(1\right)}$

Index	${x}_{1}$	${x}_{2}$	${x}_{3}$	${x}_{4}$	${x}_{5}$
${C}_{1}$	${\text{VHI}}$	${\text{HI}}$	${\text{BAI}}$	${\text{BAI}}$	${\text{AI}}$
${C}_{2}$	${\text{VHI}}$	${\text{HI}}$	${\text{LI}}$	${\text{LI}}$	${\text{AAI}}$
${C}_{3}$	${\text{VHI}}$	${\text{BAI}}$	${\text{LI}}$	${\text{LI}}$	${\text{VHI}}$
${C}_{4}$	${\text{VHI}}$	${\text{VHI}}$	${\text{BAI}}$	${\text{BAI}}$	${\text{AAI}}$
${C}_{5}$	${\text{VHI}}$	${\text{AI}}$	${\text{AI}}$	${\text{AI}}$	${\text{AI}}$
${C}_{6}$	${\text{VHI}}$	${\text{AI}}$	${\text{BAI}}$	${\text{BAI}}$	${\text{VHI}}$
${C}_{7}$	${\text{LI}}$	${\text{VLI}}$	${\text{HI}}$	${\text{HI}}$	${\text{VHI}}$
${C}_{8}$	${\text{VLI}}$	${\text{VLI}}$	${\text{VHI}}$	${\text{VHI}}$	${\text{AI}}$
${C}_{9}$	${\text{LI}}$	${\text{BAI}}$	${\text{HI}}$	${\text{HI}}$	${\text{BAI}}$
${C}_{10}$	${\text{VLI}}$	${\text{LI}}$	${\text{HI}}$	${\text{HI}}$	${\text{VHI}}$
${C}_{11}$	${\text{VHI}}$	${\text{HI}}$	${\text{AI}}$	${\text{BAI}}$	${\text{AAI}}$
${C}_{12}$	${\text{VHI}}$	${\text{VHI}}$	${\text{VHI}}$	${\text{BAI}}$	${\text{VHI}}$
${C}_{13}$	${\text{VLI}}$	${\text{LI}}$	${\text{AI}}$	${\text{AAI}}$	${\text{VHI}}$

**Table 8:** ${D}^{\left(2\right)}$ evaluation value ${L}^{\left(2\right)}$

Index	${x}_{1}$	${x}_{2}$	${x}_{3}$	${x}_{4}$	${x}_{5}$
${C}_{1}$	${\text{VHI}}$	${\text{HI}}$	${\text{BAI}}$	${\text{BAI}}$	${\text{AI}}$
${C}_{2}$	${\text{VHI}}$	${\text{AAI}}$	${\text{VLI}}$	${\text{VLI}}$	${\text{AI}}$
${C}_{3}$	${\text{HI}}$	${\text{AI}}$	${\text{LI}}$	${\text{LI}}$	${\text{HI}}$
${C}_{4}$	${\text{VHI}}$	${\text{VHI}}$	${\text{AI}}$	${\text{AI}}$	${\text{HI}}$
${C}_{5}$	${\text{VHI}}$	${\text{HI}}$	${\text{HI}}$	${\text{HI}}$	${\text{HI}}$
${C}_{6}$	${\text{VHI}}$	${\text{AAI}}$	${\text{AI}}$	${\text{AI}}$	${\text{VHI}}$
${C}_{7}$	${\text{VLI}}$	${\text{VLI}}$	${\text{AI}}$	${\text{AI}}$	${\text{VHI}}$
${C}_{8}$	${CLI}$	${\text{LI}}$	${\text{VHI}}$	${\text{VHI}}$	${\text{AI}}$
${C}_{9}$	${\text{AI}}$	${\text{AI}}$	${CHI}$	${CHI}$	${\text{AI}}$
${C}_{10}$	${\text{VLI}}$	${\text{LI}}$	${\text{AI}}$	${\text{AI}}$	${\text{VHI}}$
${C}_{11}$	${\text{VHI}}$	${\text{AAI}}$	${\text{AAI}}$	${\text{BAI}}$	${\text{HI}}$
${C}_{12}$	${\text{VHI}}$	${\text{VHI}}$	${\text{HI}}$	${\text{BAI}}$	${\text{VHI}}$
${C}_{13}$	${\text{BAI}}$	${\text{AI}}$	${\text{HI}}$	${\text{VHI}}$	${\text{VHI}}$

**Table 9:** ${D}^{\left(3\right)}$ evaluation value ${L}^{\left(3\right)}$

Index	${x}_{1}$	${x}_{2}$	${x}_{3}$	${x}_{4}$	${x}_{5}$
${C}_{1}$	${\text{VHI}}$	${\text{HI}}$	${\text{BAI}}$	${\text{BAI}}$	${\text{AI}}$
${C}_{2}$	${\text{VHI}}$	${\text{HI}}$	${\text{LI}}$	${\text{LI}}$	${\text{AAI}}$
${C}_{3}$	${\text{VHI}}$	${\text{AI}}$	${\text{LI}}$	${\text{LI}}$	${\text{VHI}}$
${C}_{4}$	${\text{VHI}}$	${\text{VHI}}$	${\text{AI}}$	${\text{AI}}$	${\text{HI}}$
${C}_{5}$	${\text{VHI}}$	${\text{AAI}}$	${\text{AAI}}$	${\text{AAI}}$	${\text{AAI}}$
${C}_{6}$	${\text{HI}}$	${\text{AAI}}$	${\text{AI}}$	${\text{AI}}$	${\text{HI}}$
${C}_{7}$	${\text{VLI}}$	${\text{LI}}$	${\text{AAI}}$	${\text{AAI}}$	${\text{VHI}}$
${C}_{8}$	${\text{LI}}$	${\text{LI}}$	${\text{VHI}}$	${\text{HI}}$	${\text{AI}}$
${C}_{9}$	${\text{AI}}$	${\text{AI}}$	${\text{VHI}}$	${\text{VHI}}$	${\text{AI}}$
${C}_{10}$	${\text{VLI}}$	${\text{VLI}}$	${\text{AAI}}$	${\text{AAI}}$	${\text{VHI}}$
${C}_{11}$	${\text{HI}}$	${\text{AAI}}$	${\text{BAI}}$	${\text{BAI}}$	${\text{AI}}$
${C}_{12}$	${\text{VHI}}$	${\text{VHI}}$	${\text{HI}}$	${\text{BAI}}$	${\text{VHI}}$
${C}_{13}$	${\text{LI}}$	${\text{AI}}$	${\text{HI}}$	${\text{HI}}$	${\text{VHI}}$

**Table 10:** ${D}^{\left(4\right)}$ evaluation value ${L}^{\left(4\right)}$

Index	${x}_{1}$	${x}_{2}$	${x}_{3}$	${x}_{4}$	${x}_{5}$
${C}_{1}$	${\text{VHI}}$	${\text{HI}}$	${\text{BAI}}$	${\text{BAI}}$	${\text{AI}}$
${C}_{2}$	${\text{VHI}}$	${\text{AAI}}$	${\text{VLI}}$	${\text{VLI}}$	${\text{AI}}$
${C}_{3}$	${\text{VHI}}$	${\text{AAI}}$	${\text{LI}}$	${\text{LI}}$	${\text{VHI}}$
${C}_{4}$	${\text{VHI}}$	${\text{VHI}}$	${\text{AI}}$	${\text{AI}}$	${\text{HI}}$
${C}_{5}$	${\text{VHI}}$	${\text{AI}}$	${\text{AI}}$	${\text{AI}}$	${\text{AI}}$
${C}_{6}$	${\text{VHI}}$	${\text{HI}}$	${\text{AI}}$	${\text{AI}}$	${\text{VHI}}$
${C}_{7}$	${\text{LI}}$	${\text{VLI}}$	${\text{HI}}$	${\text{HI}}$	${\text{VHI}}$
${C}_{8}$	${CLI}$	${\text{VLI}}$	${\text{VHI}}$	${\text{VHI}}$	${\text{AAI}}$
${C}_{9}$	${\text{BAI}}$	${\text{AI}}$	${\text{VHI}}$	${\text{VHI}}$	${\text{AI}}$
${C}_{10}$	${\text{VLI}}$	${\text{LI}}$	${\text{HI}}$	${\text{HI}}$	${\text{VHI}}$
${C}_{11}$	${\text{VHI}}$	${\text{HI}}$	${\text{AI}}$	${\text{BAI}}$	${\text{AI}}$
${C}_{12}$	${\text{VHI}}$	${\text{VHI}}$	${\text{HI}}$	${\text{BAI}}$	${\text{VHI}}$
${C}_{13}$	${\text{BAI}}$	${\text{AI}}$	${\text{AI}}$	${\text{AAI}}$	${\text{HI}}$

**Table 11:** ${D}^{\left(5\right)}$ evaluation value ${L}^{\left(5\right)}$

Index	${x}_{1}$	${x}_{2}$	${x}_{3}$	${x}_{4}$	${x}_{5}$
${C}_{1}$	${\text{VHI}}$	${\text{HI}}$	${\text{BAI}}$	${\text{BAI}}$	${\text{AI}}$
${C}_{2}$	${\text{VHI}}$	${\text{HI}}$	${\text{AI}}$	${\text{AI}}$	${\text{AAI}}$
${C}_{3}$	${\text{VHI}}$	${\text{HI}}$	${\text{AI}}$	${\text{AI}}$	${\text{AAI}}$
${C}_{4}$	${\text{VHI}}$	${\text{VHI}}$	${\text{AAI}}$	${\text{AAI}}$	${\text{HI}}$
${C}_{5}$	${\text{VHI}}$	${\text{HI}}$	${\text{HI}}$	${\text{HI}}$	${\text{HI}}$
${C}_{6}$	${\text{VHI}}$	${\text{HI}}$	${\text{AAI}}$	${\text{AAI}}$	${\text{VHI}}$
${C}_{7}$	${\text{VLI}}$	${\text{VLI}}$	${\text{VHI}}$	${\text{VHI}}$	${\text{AAI}}$
${C}_{8}$	${\text{VLI}}$	${\text{LI}}$	${\text{VHI}}$	${\text{VHI}}$	${\text{HI}}$
${C}_{9}$	${\text{AAI}}$	${\text{AAI}}$	${\text{VHI}}$	${\text{VHI}}$	${\text{AAI}}$
${C}_{10}$	${\text{VLI}}$	${\text{VLI}}$	${\text{AAI}}$	${\text{AAI}}$	${\text{HI}}$
${C}_{11}$	${\text{VHI}}$	${\text{HI}}$	${\text{AAI}}$	${\text{BAI}}$	${\text{HI}}$
${C}_{12}$	${\text{VHI}}$	${\text{VHI}}$	${\text{HI}}$	${\text{BAI}}$	${\text{VHI}}$
${C}_{13}$	${\text{LI}}$	${\text{LI}}$	${\text{HI}}$	${\text{AAI}}$	${\text{HI}}$

#### Alternatives selection

As can be seen from Tables 7, 8, 9, 10, and 11, it is difficult to determine the best HFU system based on the decision matrix provided by the five experts. For some attributes, there seems to be little difference among the respective experts. However, determining expert weights and attribute weights are not straightforward. That is why, as described in “Integrated Group Decision method”, the proposed MAGDM method should be used to select the best alternative. The HFU system evaluation process includes the following 15 steps.

1. Transform $${L}^{\left(t\right)}$$ into $${A}^{{{\prime}}\left(t\right)}$$.
  The evaluation matrices ${L}^{\left(1\right)},{L}^{\left(2\right)},{L}^{\left(3\right)},{L}^{\left(4\right)},{L}^{\left(5\right)}$ are transformed into IVq-ROFN decision matrices ${A}^{{{\prime}}\left(1\right)},{A^{\prime}}^{\left(2\right)},{A}^{{{\prime}}\left(3\right)},{A}^{{{\prime}}\left(4\right)},{A}^{{{\prime}}\left(5\right)}$ using Table 1.
2. Determine the *q* value.
  It is found that when $q$ is greater than or equal to 2, all elements of ${A}^{{{\prime}}\left(1\right)},{A^{\prime}}^{\left(2\right)},{A}^{{{\prime}}\left(3\right)},{A}^{{{\prime}}\left(4\right)},{A}^{{{\prime}}\left(5\right)}$ satisfy the definition of IVq-ROFS. $q$ is set to 3 in this case.
3. Standardize $${A}^{{{\prime}}\left(t\right)}$$.
  The cost-type attributes (C1, C9) are then converted into benefit-type attributes by Eqs. (24) and (25). ${A}^{{{\prime}}\left(1\right)},{A^{\prime}}^{\left(2\right)},{A}^{{{\prime}}\left(3\right)},{A}^{{{\prime}}\left(4\right)},{A}^{{{\prime}}\left(5\right)}$ are transformed into ${A}^{\left(1\right)},{A}^{\left(2\right)},{A}^{\left(3\right)},{A}^{\left(4\right)},{A}^{\left(5\right)}$ as shown in Tables 12, 13, 14, 15, and 16.
4. Determine the preference pairs set $P$.

**Table 12:** Evaluation value ${A}^{\left(1\right)}$

Index	${x}_{1}$	${x}_{2}$	${x}_{3}$	${x}_{4}$	${x}_{5}$
${C}_{1}$	$(\left[{0.10,0.20}\right],[{0.80,0.90}])$	$(\left[{0.20,0.35}\right],[{0.65,0.80}])$	$(\left[{0.55,0.65}\right],[{0.35,0.45}])$	$(\left[{0.55,0.65}\right],[{0.35,0.45}])$	$(\left[{0.45,0.55}\right],[{0.45,0.55}])$
${C}_{2}$	$([{0.80,0.90}],[{0.10,0.20}])$	$([{0.65,0.80}],[{0.20,0.35}])$	$([{0.20,0.35}],[{0.65,0.80}])$	$([{0.20,0.35}],[{0.65,0.80}])$	$([{0.55,0.65}],[{0.35,0.45}])$
${C}_{3}$	$([{0.80,0.90}],[{0.10,0.20}])$	$([{0.35,0.45}],[{0.55,0.65}])$	$([{0.20,0.35}],[{0.65,0.80}])$	$([{0.20,0.35}],[{0.65,0.80}])$	$([{0.80,0.90}],[{0.10,0.20}])$
${C}_{4}$	$([{0.80,0.90}],[{0.10,0.20}])$	$([{0.80,0.90}],[{0.10,0.20}])$	$([{0.35,0.45}],[{0.55,0.65}])$	$([{0.35,0.45}],[{0.55,0.65}])$	$([{0.55,0.65}],[{0.35,0.45}])$
${C}_{5}$	$([{0.80,0.90}],[{0.10,0.20}])$	$([{0.45,0.55}],[{0.45,0.55}])$	$([{0.45,0.55}],[{0.45,0.55}])$	$([{0.45,0.55}],[{0.45,0.55}])$	$([{0.45,0.55}],[{0.45,0.55}])$
${C}_{6}$	$([{0.80,0.90}],[{0.10,0.20}])$	$([{0.45,0.55}],[{0.45,0.55}])$	$([{0.35,0.45}],[{0.55,0.65}])$	$([{0.35,0.45}],[{0.55,0.65}])$	$([{0.80,0.90}],[{0.10,0.20}])$
${C}_{7}$	$([{0.20,0.35}],[{0.65,0.80}])$	$([{0.10,0.20}],[{0.80,0.90}])$	$([{0.65,0.80}],[{0.20,0.35}])$	$([{0.65,0.80}],[{0.20,0.35}])$	$([{0.80,0.90}],[{0.10,0.20}])$
${C}_{8}$	$([{0.10,0.20}],[{0.80,0.90}])$	$([{0.10,0.20}],[{0.80,0.90}])$	$([{0.80,0.90}],[{0.10,0.20}])$	$([{0.80,0.90}],[{0.10,0.20}])$	$([{0.45,0.55}],[{0.45,0.55}])$
${C}_{9}$	$(\left[{0.65,0.80}\right],[{0.20,0.35}])$	$(\left[{0.55,0.65}\right],[{0.35,0.45}])$	$(\left[{0.20,0.35}\right],[{0.65,0.80}])$	$(\left[{0.20,0.35}\right],[{0.65,0.80}])$	$(\left[{0.55,0.65}\right],[{0.35,0.45}])$
${C}_{10}$	$([{0.10,0.20}],[{0.80,0.90}])$	$([{0.20,0.35}],[{0.65,0.80}])$	$([{0.65,0.80}],[{0.20,0.35}])$	$([{0.65,0.80}],[{0.20,0.35}])$	$([{0.80,0.90}],[{0.10,0.20}])$
${C}_{11}$	$([{0.80,0.90}],[{0.10,0.20}])$	$([{0.65,0.80}],[{0.20,0.35}])$	$([{0.45,0.55}],[{0.45,0.55}])$	$([{0.35,0.45}],[{0.55,0.65}])$	$([{0.55,0.65}],[{0.35,0.45}])$
${C}_{12}$	$([{0.80,0.90}],[{0.10,0.20}])$	$([{0.80,0.90}],[{0.10,0.20}])$	$([{0.80,0.90}],[{0.10,0.20}])$	$([{0.35,0.45}],[{0.55,0.65}])$	$([{0.80,0.90}],[{0.10,0.20}])$
${C}_{13}$	$([{0.10,0.20}],[{0.80,0.90}])$	$([{0.20,0.35}],[{0.65,0.80}])$	$([{0.45,0.55}],[{0.45,0.55}])$	$([{0.55,0.65}],[{0.35,0.45}])$	$([{0.80,0.90}],[{0.10,0.20}])$

**Table 13:** Evaluation value ${A}^{\left(2\right)}$

Index	${x}_{1}$	${x}_{2}$	${x}_{3}$	${x}_{4}$	${x}_{5}$
${C}_{1}$	$(\left[{0.10,0.20}\right],[{0.80,0.90}])$	$(\left[{0.20,0.35}\right],[{0.65,0.80}])$	$(\left[{0.55,0.65}\right],[{0.35,0.45}])$	$(\left[{0.55,0.65}\right],[{0.35,0.45}])$	$(\left[{0.45,0.55}\right],[{0.45,0.55}])$
${C}_{2}$	$([{0.80,0.90}],[{0.10,0.20}])$	$([{0.55,0.65}],[{0.35,0.45}])$	$([{0.10,0.20}],[{0.80,0.90}])$	$([{0.10,0.20}],[{0.80,0.90}])$	$([{0.45,0.55}],[{0.45,0.55}])$
${C}_{3}$	$([{0.65,0.80}],[{0.20,0.35}])$	$([{0.45,0.55}],[{0.45,0.55}])$	$([{0.20,0.35}],[{0.65,0.80}])$	$([{0.20,0.35}],[{0.65,0.80}])$	$([{0.65,0.80}],[{0.20,0.35}])$
${C}_{4}$	$([{0.80,0.90}],[{0.10,0.20}])$	$([{0.80,0.90}],[{0.10,0.20}])$	$([{0.45,0.55}],[{0.45,0.55}])$	$([{0.45,0.55}],[{0.45,0.55}])$	$([{0.65,0.80}],[{0.20,0.35}])$
${C}_{5}$	$([{0.80,0.90}],[{0.10,0.20}])$	$([{0.65,0.80}],[{0.20,0.35}])$	$([{0.65,0.80}],[{0.20,0.35}])$	$([{0.65,0.80}],[{0.20,0.35}])$	$([{0.65,0.80}],[{0.20,0.35}])$
${C}_{6}$	$([{0.80,0.90}],[{0.10,0.20}])$	$([{0.55,0.65}],[{0.35,0.45}])$	$([{0.45,0.55}],[{0.45,0.55}])$	$([{0.45,0.55}],[{0.45,0.55}])$	$([{0.80,0.90}],[{0.10,0.20}])$
${C}_{7}$	$([{0.10,0.20}],[{0.80,0.90}])$	$([{0.10,0.20}],[{0.80,0.90}])$	$([{0.45,0.55}],[{0.45,0.55}])$	$([{0.45,0.55}],[{0.45,0.55}])$	$([{0.80,0.90}],[{0.10,0.20}])$
${C}_{8}$	$([{0.05,0.05}],[{0.90,0.95}])$	$([{0.20,0.35}],[{0.65,0.80}])$	$([{0.80,0.90}],[{0.10,0.20}])$	$([{0.80,0.90}],[{0.10,0.20}])$	$([{0.45,0.55}],[{0.45,0.55}])$
${C}_{9}$	$(\left[{0.45,0.55}\right],[{0.45,0.55}])$	$(\left[{0.45,0.55}\right],[{0.45,0.55}])$	$(\left[{0.05,0.05}\right],[{0.90,0.95}])$	$(\left[{0.05,0.05}\right],[{0.90,0.95}])$	$(\left[{0.45,0.55}\right],[{0.45,0.55}])$
${C}_{10}$	$([{0.10,0.20}],[{0.80,0.90}])$	$([{0.20,0.35}],[{0.65,0.80}])$	$([{0.45,0.55}],[{0.45,0.55}])$	$([{0.45,0.55}],[{0.45,0.55}])$	$([{0.80,0.90}],[{0.10,0.20}])$
${C}_{11}$	$([{0.80,0.90}],[{0.10,0.20}])$	$([{0.55,0.65}],[{0.35,0.45}])$	$([{0.55,0.65}],[{0.35,0.45}])$	$([{0.35,0.45}],[{0.55,0.65}])$	$([{0.65,0.80}],[{0.20,0.35}])$
${C}_{12}$	$([{0.80,0.90}],[{0.10,0.20}])$	$([{0.80,0.90}],[{0.10,0.20}])$	$([{0.65,0.80}],[{0.20,0.35}])$	$([{0.35,0.45}],[{0.55,0.65}])$	$([{0.80,0.90}],[{0.10,0.20}])$
${C}_{13}$	$([{0.35,0.45}],[{0.55,0.65}])$	$([{0.45,0.55}],[{0.45,0.55}])$	$([{0.65,0.80}],[{0.20,0.35}])$	$([{0.80,0.90}],[{0.10,0.20}])$	$([{0.80,0.90}],[{0.10,0.20}])$

**Table 14:** Evaluation value *A*^(3)^

Index	${x}_{1}$	${x}_{2}$	${x}_{3}$	${x}_{4}$	${x}_{5}$
${C}_{1}$	$(\left[{0.10,0.20}\right],[{0.80,0.90}])$	$(\left[{0.20,0.35}\right],[{0.65,0.80}])$	$(\left[{0.55,0.65}\right],[{0.35,0.45}])$	$(\left[{0.55,0.65}\right],[{0.35,0.45}])$	$(\left[{0.45,0.55}\right],[{0.45,0.55}])$
${C}_{2}$	$([{0.80,0.90}],[{0.10,0.20}])$	$([{0.65,0.80}],[{0.20,0.35}])$	$([{0.20,0.35}],[{0.65,0.80}])$	$([{0.20,0.35}],[{0.65,0.80}])$	$([{0.55,0.65}],[{0.35,0.45}])$
${C}_{3}$	$([{0.80,0.90}],[{0.10,0.20}])$	$([{0.45,0.55}],[{0.45,0.55}])$	$([{0.20,0.35}],[{0.65,0.80}])$	$([{0.20,0.35}],[{0.65,0.80}])$	$([{0.80,0.90}],[{0.10,0.20}])$
${C}_{4}$	$([{0.80,0.90}],[{0.10,0.20}])$	$([{0.80,0.90}],[{0.10,0.20}])$	$([{0.45,0.55}],[{0.45,0.55}])$	$([{0.45,0.55}],[{0.45,0.55}])$	$([{0.65,0.80}],[{0.20,0.35}])$
${C}_{5}$	$([{0.80,0.90}],[{0.10,0.20}])$	$([{0.55,0.65}],[{0.35,0.45}])$	$([{0.55,0.65}],[{0.35,0.45}])$	$([{0.55,0.65}],[{0.35,0.45}])$	$([{0.55,0.65}],[{0.35,0.45}])$
${C}_{6}$	$([{0.65,0.80}],[{0.20,0.35}])$	$([{0.55,0.65}],[{0.35,0.45}])$	$([{0.45,0.55}],[{0.45,0.55}])$	$([{0.45,0.55}],[{0.45,0.55}])$	$([{0.65,0.80}],[0.2{0,0.35}])$
${C}_{7}$	$([{0.10,0.20}],[{0.80,0.90}])$	$([{0.20,0.35}],[{0.65,0.80}])$	$([{0.55,0.65}],[{0.35,0.45}])$	$([{0.55,0.65}],[{0.35,0.45}])$	$([{0.80,0.90}],[{0.10,0.20}])$
${C}_{8}$	$([{0.20,0.35}],[{0.65,0.80}])$	$([{0.20,0.35}],[{0.65,0.80}])$	$([{0.80,0.90}],[{0.10,0.20}])$	$([{0.65,0.80}],[{0.20,0.35}])$	$([{0.45,0.55}],[{0.45,0.55}])$
${C}_{9}$	$(\left[{0.45,0.55}\right],[{0.45,0.55}])$	$(\left[{0.45,0.55}\right],[{0.45,0.55}])$	$(\left[{0.10,0.20}\right],[{0.80,0.90}])$	$(\left[{0.10,0.20}\right],[{0.80,0.90}])$	$(\left[{0.45,0.55}\right],[{0.45,0.55}])$
${C}_{10}$	$([{0.10,0.20}],[{0.80,0.90}])$	$([{0.10,0.20}],[{0.80,0.90}])$	$([{0.55,0.65}],[{0.35,0.45}])$	$([{0.55,0.65}],[{0.35,0.45}])$	$([{0.80,0.90}],[{0.10,0.20}])$
${C}_{11}$	$([{0.65,0.80}],[{0.20,0.35}])$	$([{0.55,0.65}],[{0.35,0.45}])$	$([{0.35,0.45}],[{0.55,0.65}])$	$([{0.35,0.45}],[{0.55,0.65}])$	$([{0.45,0.55}],[{0.45,0.55}])$
${C}_{12}$	$([{0.80,0.90}],[{0.10,0.20}])$	$([{0.80,0.90}],[{0.10,0.20}])$	$([{0.65,0.8}0],[{0.20,0.35}])$	$([{0.35,0.45}],[{0.55,0.65}])$	$([{0.80,0.90}],[{0.10,0.20}])$
${C}_{13}$	$([{0.20,0.35}],[{0.65,0.80}])$	$([{0.45,0.55}],[{0.45,0.55}])$	$([{0.65,0.80}],[{0.20,0.35}])$	$([{0.65,0.80}],[{0.20,0.35}])$	$([{0.80,0.90}],[{0.10,0.20}])$

**Table 15:** Evaluation value *A*^(4)^

Index	${x}_{1}$	${x}_{2}$	${x}_{3}$	${x}_{4}$	${x}_{5}$
${C}_{1}$	$(\left[{0.10,0.20}\right],[{0.80,0.90}])$	$(\left[{0.20,0.35}\right],[{0.65,0.80}])$	$(\left[{0.55,0.65}\right],[{0.35,0.45}])$	$(\left[{0.55,0.65}\right],[{0.35,0.45}])$	$(\left[{0.45,0.55}\right],[{0.45,0.55}])$
${C}_{2}$	$([{0.80,0.90}],[{0.10,0.20}])$	$([{0.55,0.65}],[{0.35,0.45}])$	$([{0.10,0.20}],[{0.80,0.90}])$	$([{0.10,0.20}],[{0.80,0.90}])$	$([{0.45,0.55}],[{0.45,0.55}])$
${C}_{3}$	$([{0.80,0.90}],[{0.10,0.20}])$	$([{0.55,0.65}],[{0.35,0.45}])$	$([{0.20,0.35}],[{0.65,0.80}])$	$([{0.20,0.35}],[{0.65,0.80}])$	$([{0.80,0.90}],[{0.10,0.20}])$
${C}_{4}$	$([{0.80,0.90}],[{0.10,0.20}])$	$([{0.80,0.90}],[{0.10,0.20}])$	$([{0.45,0.55}],[{0.45,0.55}])$	$([{0.45,0.55}],[{0.45,0.55}])$	$([{0.65,0.80}],[{0.20,0.35}])$
${C}_{5}$	$([{0.80,0.90}],[{0.10,0.20}])$	$([{0.45,0.55}],[{0.45,0.55}])$	$([{0.45,0.55}],[{0.45,0.55}])$	$([{0.45,0.55}],[{0.45,0.55}])$	$([{0.45,0.55}],[{0.45,0.55}])$
${C}_{6}$	$([{0.80,0.90}],[{0.10,0.20}])$	$([{0.65,0.80}],[{0.20,0.35}])$	$([{0.45,0.55}],[{0.45,0.55}])$	$([{0.45,0.55}],[{0.45,0.55}])$	$([{0.80,0.90}],[{0.10,0.20}])$
${C}_{7}$	$([{0.20,0.35}],[{0.65,0.80}])$	$([{0.10,0.20}],[{0.80,0.90}])$	$([{0.65,0.80}],[{0.20,0.35}])$	$([{0.65,0.80}],[{0.20,0.35}])$	$([{0.80,0.90}],[{0.10,0.20}])$
${C}_{8}$	$([{0.05,0.05}],[{0.90,0.95}])$	$([{0.10,0.20}],[{0.80,0.90}])$	$([{0.80,0.90}],[{0.10,0.20}])$	$([{0.80,0.90}],[{0.10,0.20}])$	$([{0.55,0.65}],[{0.35,0.45}])$
${C}_{9}$	$(\left[{0.55,0.65}\right],[{0.35,0.45}])$	$(\left[{0.45,0.55}\right],[{0.45,0.55}])$	$(\left[{0.10,0.20}\right],[{0.80,0.90}])$	$(\left[{0.10,0.20}\right],[{0.80,0.90}])$	$(\left[{0.45,0.55}\right],[{0.45,0.55}])$
${C}_{10}$	$([{0.10,0.20}],[{0.80,0.90}])$	$([{0.20,0.35}],[{0.65,0.80}])$	$([{0.65,0.80}],[{0.20,0.35}])$	$([{0.65,0.80}],[{0.20,0.35}])$	$([{0.80,0.90}],[{0.10,0.20}])$
${C}_{11}$	$([{0.80,0.90}],[{0.10,0.20}])$	$([{0.65,0.80}],[{0.20,0.35}])$	$([{0.45,0.55}],[{0.45,0.55}])$	$([{0.35,0.45}],[{0.55,0.65}])$	$([{0.45,0.55}],[{0.45,0.55}])$
${C}_{12}$	$([{0.80,0.90}],[{0.10,0.20}])$	$([{0.80,0.90}],[{0.10,0.20}])$	$([{0.65,0.80}],[{0.20,0.35}])$	$([{0.35,0.45}],[{0.55,0.65}])$	$([{0.80,0.90}],[{0.10,0.20}])$
${C}_{13}$	$([{0.35,0.45}],[{0.55,0.65}])$	$([{0.45,0.55}],[{0.45,0.55}])$	$([{0.45,0.55}],[{0.45,0.55}])$	$([{0.55,0.65}],[{0.35,0.45}])$	$([{0.65,0.80}],[{0.20,0.35}])$

**Table 16:** Evaluation value *A*^(5)^

Index	$${x}_{1}$$	$${x}_{2}$$	$${x}_{3}$$	$${x}_{4}$$	$${x}_{5}$$
$${C}_{1}$$	$$(\left[{0.10,0.20}\right],[{0.80,0.90}])$$	$$(\left[{0.20,0.35}\right],[{0.65,0.80}])$$	$$(\left[{0.55,0.65}\right],[{0.35,0.45}])$$	$$(\left[{0.55,0.65}\right],[{0.35,0.45}])$$	$$(\left[{0.45,0.55}\right],[{0.45,0.55}])$$
$${C}_{2}$$	$$([{0.80,0.90}],[{0.10,0.20}])$$	$$([{0.65,0.80}],[{0.20,0.35}])$$	$$([{0.45,0.55}],[{0.45,0.55}])$$	$$([{0.45,0.55}],[{0.45,0.55}])$$	$$([{0.55,0.65}],[{0.35,0.45}])$$
$${C}_{3}$$	$$([{0.80,0.90}],[{0.10,0.20}])$$	$$([{0.65,0.80}],[{0.20,0.35}])$$	$$([{0.45,0.55}],[{0.45,0.55}])$$	$$([{0.45,0.55}],[{0.45,0.55}])$$	$$([{0.55,0.65}],[{0.35,0.45}])$$
$${C}_{4}$$	$$([{0.80,0.90}],[{0.10,0.20}])$$	$$([{0.80,0.90}],[{0.10,0.20}])$$	$$([{0.55,0.65}],[{0.35,0.45}])$$	$$([{0.55,0.65}],[{0.35,0.45}])$$	$$([{0.65,0.80}],[{0.20,0.35}])$$
$${C}_{5}$$	$$([{0.80,0.90}],[{0.10,0.20}])$$	$$([{0.65,0.80}],[{0.20,0.35}])$$	$$([{0.65,0.80}],[{0.20,0.35}])$$	$$([{0.65,0.80}],[{0.20,0.35}])$$	$$([{0.65,0.80}],[{0.20,0.35}])$$
$${C}_{6}$$	$$([{0.80,0.90}],[{0.10,0.20}])$$	$$([{0.65,0.80}],[{0.20,0.35}])$$	$$([{0.55,0.65}],[{0.35,0.45}])$$	$$([{0.55,0.65}],[{0.35,0.45}])$$	$$([{0.80,0.90}],[{0.10,0.20}])$$
$${C}_{7}$$	$$([{0.10,0.20}],[{0.80,0.90}])$$	$$([{0.10,0.20}],[{0.80,0.90}])$$	$$([{0.80,0.90}],[{0.10,0.20}])$$	$$([{0.80,0.90}],[{0.10,0.20}])$$	$$([{0.55,0.65}],[{0.35,0.45}])$$
$${C}_{8}$$	$$([{0.10,0.20}],[{0.80,0.90}])$$	$$([{0.20,0.35}],[{0.65,0.80}])$$	$$([{0.80,0.90}],[{0.10,0.20}])$$	$$([{0.80,0.90}],[{0.10,0.20}])$$	$$([{0.65,0.80}],[{0.20,0.35}])$$
$${C}_{9}$$	$$(\left[{0.35,0.45}\right],[{0.55,0.65}])$$	$$(\left[{0.35,0.45}\right],[{0.55,0.65}])$$	$$(\left[{0.10,0.20}\right],[{0.80,0.90}])$$	$$(\left[{0.10,0.20}\right],[{0.80,0.90}])$$	$$(\left[{0.35,0.45}\right],[{0.55,0.65}])$$
$${C}_{10}$$	$$([{0.10,0.20}],[{0.80,0.90}])$$	$$([{0.10,0.20}],[{0.80,0.90}])$$	$$([{0.55,0.65}],[{0.35,0.45}])$$	$$([{0.55,0.65}],[{0.35,0.45}])$$	$$([{0.65,0.80}],[{0.20,0.35}])$$
$${C}_{11}$$	$$([{0.80,0.90}],[{0.10,0.20}])$$	$$([{0.65,0.80}],[{0.20,0.35}])$$	$$([{0.55,0.65}],[{0.35,0.45}])$$	$$([{0.35,0.45}],[{0.55,0.65}])$$	$$([{0.65,0.80}],[{0.20,0.35}])$$
$${C}_{12}$$	$$([{0.80,0.90}],[{0.10,0.20}])$$	$$([{0.80,0.90}],[{0.10,0.20}])$$	$$([{0.65,0.80}],[{0.20,0.35}])$$	$$([{0.35,0.45}],[{0.55,0.65}])$$	$$([{0.80,0.90}],[{0.10,0.20}])$$
$${C}_{13}$$	$$([{0.20,0.35}],[{0.65,0.80}])$$	$$([{0.20,0.35}],[{0.65,0.80}])$$	$$([{0.65,0.80}],[{0.20,0.35}])$$	$$([{0.55,0.65}],[{0.35,0.45}])$$	$$([{0.65,0.80}],[{0.20,0.35}])$$
  The preference pairs’ set $$P=\left\{\left({5,4}\right),\left({5,1}\right),\left({3,2}\right),\left({1,2}\right)\right\}$$ was determined in advance.
5. Derive expert weight matrix $$\lambda $$.
  According to the decision matrix $${A}^{\left(t\right)}$$, the attribute weight $${w}^{(t)}$$ is solved using the LINMAP model. The steps of the solution are given in Eqs. (26)–(33). The IVq-ROFWFAWA operator is used to aggregate row elements of the matrices in Tables 12, 13, 14, 15, and 16. The matrix $${\overline{D}}$$ is obtained using Eqs. (34)–(36). The expert weight matrix can be obtained using Eqs. (37)–(44). The result of the expert weight matrix $$\lambda $$ (keeping four decimal places) is as follows:
  $$\begin{aligned}
\lambda  = &\left( {\begin{array}{*{20}l}    {\lambda ^{{\left( 1
\right)}} }  \\    {\lambda ^{{\left( 2 \right)}} }  \\    {\lambda
^{{\left( 3 \right)}} }  \\    {\lambda ^{{\left( 4 \right)}} }  \\
{\lambda ^{{\left( 5 \right)}} }  \\   \end{array} } \right)\\
 &=
\left( {\begin{array}{c@{\quad}c@{\quad}c@{\quad}c@{\quad}c}    {{{0.1714}}} & {{{0.2360}}} &
{{{0.2012}}} & {{{0.2069}}} & {{{0.1828}}}  \\    {{{0.2231}}} &
{{{0.1798}}} & {{{0.2303}}} & {{{0.2153}}} & {{{0.1942}}}  \\
{{{0.2192}}} & {{{0.1946}}} & {{{0.2086}}} & {{{0.2197}}} &
{{{0.1953}}}  \\    {{{0.2039}}} & {{{0.2119}}} & {{{0.2164}}} &
{{{0.2017}}} & {{{0.1973}}}  \\    {{{0.1824}}} & {{{0.1777}}} &
{{{0.1435}}} & {{{0.1564}}} & {{{0.2304}}}  \\   \end{array} }
\right).\end{aligned} $$
6. Aggregating expert decision matrix.
  Using Eq. (45) to aggregate five experts' matrices of $${A}^{(t)}$$(*t* = 1, 2, 3, 4, 5), the collective matrix $$R={({r}_{ij})}_{5\times 13}$$ is calculated as shown in Table 17.
7. Calculate each point’s similarity of *R* to the ideal point.

**Table 17:** Aggregate matrix $R$

Index	$${x}_{1}$$	$${x}_{2}$$	$${x}_{3}$$	$${x}_{4}$$	$${x}_{5}$$
$${C}_{1}$$	$$([{0.10,0.20}],[{0.80,0.90}])$$	$$([{0.20,0.35}],[{0.65,0.80}])$$	$$([{0.55,0.65}],[{0.35,0.45}])$$	$$([{0.55,0.65}],[{0.35,0.45}])$$	$$([{0.45,0.55}],[{0.45,0.55}])$$
$${C}_{2}$$	$$([{0.80,0.90}],[{0.10,0.20}])$$	$$([{0.62,0.75}],[{0.25,0.39}])$$	$$([{0.18,0.31}],[{0.72,0.84}])$$	$$([{0.18,0.31}],[{0.72,0.84}])$$	$$([{0.52,0.62}],[{0.39,0.49}])$$
$${C}_{3}$$	$$([{0.78,0.88}],[{0.12,0.23}])$$	$$([{0.52,0.62}],[{0.42,0.54}])$$	$$([{0.23,0.38}],[{0.64,0.78}])$$	$$([{0.23,0.39}],[{0.63,0.78}])$$	$$([{0.74,0.85}],[{0.16,0.28}])$$
$${C}_{4}$$	$$([{0.80,0.90}],[{0.10,0.20}])$$	$$([{0.80,0.90}],[{0.10,0.20}])$$	$$([{0.45,0.55}],[{0.46,0.56}])$$	$$([{0.45,0.55}],[{0.46,0.56}])$$	$$([{0.64,0.78}],[{0.22,0.37}])$$
$${C}_{5}$$	$$([{0.80,0.90}],[{0.10,0.20}])$$	$$([{0.57,0.69}],[{0.34,0.47}])$$	$$([{0.57,0.69}],[{0.34,0.47}])$$	$$([{0.57,0.69}],[{0.34,0.47}])$$	$$([{0.58,0.70}],[{0.32,0.46}])$$
$${C}_{6}$$	$$([{0.78,0.88}],[{0.12,0.23}])$$	$$([{0.59,0.71}],[{0.31,0.45}])$$	$$([{0.45,0.55}],[{0.46,0.56}])$$	$$([{0.45,0.55}],[{0.46,0.56}])$$	$$([{0.78,0.89}],[0.1{2,0.22}])$$
$${C}_{7}$$	$$([{0.13,0.25}],[{0.76,0.87}])$$	$$([{0.12,0.22}],[{0.78,0.89}])$$	$$([{0.64,0.76}],[{0.27,0.4}])$$	$$([{0.65,0.77}],[{0.26,0.40}])$$	$$([{0.77,0.87}],[{0.14,0.25}])$$
$${C}_{8}$$	$$([{0.09,0.13}],[{0.84,0.92}])$$	$$([{0.15,0.28}],[{0.73,0.86}])$$	$$([{0.80,0.90}],[{0.10,0.20}])$$	$$([{0.78,0.88}],[{0.12,0.23}])$$	$$([{0.54,0.65}],[{0.38,0.50}])$$
$${C}_{9}$$	$$([{0.52,0.62}],[{0.42,0.53}])$$	$$([{0.47,0.56}],[{0.45,0.55}])$$	$$([{0.10,0.17}],[{0.81,0.91}])$$	$$([{0.10,0.17}],[{0.81,0.90}])$$	$$([{0.45,0.55}],[{0.46,0.56}])$$
$${C}_{10}$$	$$([{0.10,0.20}],[{0.80,0.90}])$$	$$([{0.16,0.29}],[{0.72,0.85}])$$	$$([{0.59,0.71}],[{0.31,0.44}])$$	$$([{0.59,0.71}],[{0.31,0.44}])$$	$$([{0.78,0.88}],[{0.12,0.23}])$$
$${C}_{11}$$	$$([{0.78,0.88}],[{0.12,0.23}])$$	$$([{0.62,0.76}],[{0.25,0.39}])$$	$$([{0.48,0.58}],[{0.44,0.54}])$$	$$([{0.35,0.45}],[{0.55,0.65}])$$	$$([{0.58,0.70}],[{0.32,0.46}])$$
$${C}_{12}$$	$$([{0.80,0.90}],[{0.10,0.20}])$$	$$([{0.80,0.90}],[{0.10,0.20}])$$	$$([{0.69,0.8}3],[{0.18,0.32}])$$	$$([{0.35,0.45}],[{0.55,0.65}])$$	$$([{0.80,0.90}],[{0.10,0.20}])$$
$${C}_{13}$$	$$([{0.24,0.37}],[{0.66,0.78}])$$	$$([{0.35,0.49}],[{0.57,0.68}])$$	$$([{0.60,0.73}],[{0.30,0.45}])$$	$$([{0.66,0.77}],[{0.25,0.38}])$$	$$([{0.75,0.87}],[{0.14,0.26}])$$
  After obtaining the ideal points of each attribute of the matrix $$R$$ by Eq. (46), as shown as follows:
  $$\begin{aligned}{r}_{1}^{*}&=\left(\left[{0.55,0.65}\right],\left[{0.35,0.45}\right]\right),\\
{r}_{2}^{*}&=\left(\left[{0.80,0.90}\right],\left[{0.10,0.20}\right]\right),\\
{r}_{3}^{*}&=\left(\left[{0.78,0.88}\right],\left[{0.12,0.23}\right]\right),\\
{r}_{4}^{*}&=\left(\left[{0.80,0.90}\right],\left[{0.10,0.20}\right]\right),\\
{r}_{5}^{*}&=\left(\left[{0.80,0.90}\right],\left[{0.10,0.20}\right]\right),\\
{r}_{6}^{*}&=\left(\left[{0.78,0.89}\right],\left[{0.12,0.22}\right]\right),\\
{r}_{7}^{*}&=\left(\left[{0.77,0.87}\right],\left[{0.14,0.25}\right]\right),\\
{r}_{8}^{*}&=\left(\left[{0.80,0.90}\right],\left[{0.10,0.20}\right]\right),\\
{r}_{9}^{*}&=\left(\left[{0.52,0.62}\right],\left[{0.42,0.53}\right]\right),\\
{r}_{10}^{*}&=\left(\left[{0.78,0.88}\right],\left[{0.12,0.23}\right]\right),\\
{r}_{11}^{*}&=\left(\left[{0.78,0.88}\right],\left[{0.12,0.23}\right]\right),\\
{r}_{12}^{*}&=\left(\left[{0.80,0.90}\right],\left[{0.10,0.20}\right]\right),\\
{r}_{13}^{*}&=\left(\left[{0.75,0.87}\right],\left[{0.14,0.26}\right]\right).
 \end{aligned}$$
  We calculate the similarity for each point of $$R$$ by Eqs. (47) and (48), which are shown in Table 18.
  8. Calculate the weighted similarity of *R*.

**Table 18:** The similarity of aggregate matrix *R*

Index	$${x}_{1}$$	$${x}_{2}$$	$${x}_{3}$$	$${x}_{4}$$	$${x}_{5}$$
$${C}_{1}$$	0.085	0.151	1.000	1.000	0.560
$${C}_{2}$$	1.000	0.512	0.020	0.021	0.268
$${C}_{3}$$	1.000	0.278	0.044	0.045	0.880
$${C}_{4}$$	1.000	1.000	0.176	0.178	0.569
$${C}_{5}$$	1.000	0.371	0.381	0.382	0.403
$${C}_{6}$$	0.992	0.440	0.188	0.189	1.000
$${C}_{7}$$	0.017	0.014	0.611	0.622	1.000
$${C}_{8}$$	0.005	0.015	1.000	0.933	0.311
$${C}_{9}$$	1.000	0.793	0.139	0.140	0.746
$${C}_{10}$$	0.010	0.020	0.455	0.455	1.000
$${C}_{11}$$	1.000	0.554	0.224	0.092	0.429
$${C}_{12}$$	1.000	1.000	0.710	0.085	1.000
$${C}_{13}$$	0.046	0.112	0.517	0.669	1.000
  Let the attribute weight of $$R$$ be $$w=({w}_{1},{w}_{2},\dots ,{w}_{n})$$, the weighted average $${Eqr}_{g}$$ and $${Eqr}_{l}$$ are shown in Eqs. (49) and (50), and the results are
  $$\begin{aligned}Eq{r}_{1}&={0.085}{w}_{1}+{w}_{2}+{w}_{3}+{w}_{4}+{w}_{5}+0.992{w}_{6}+{0.017}{w}_{7}+0.005{w}_{8}+{w}_{9}+{0.01}{w}_{10}+{w}_{11}+{w}_{12}+{0.046}{w}_{13},\\
Eq{r}_{2}&={0.151}{w}_{1}+{0.512}{w}_{2}+{0.278}{w}_{3}+{w}_{4}+{0.371}{w}_{5}+{0.44}{w}_{6}+{0.014}{w}_{7}+{0.015}{w}_{8}+{0.793}{w}_{9}+{0.02}{w}_{10}\\
&+{0.554}{w}_{11}+{w}_{12}+{0.112}{w}_{13},\\
Eq{r}_{3}&={w}_{1}+0.02{w}_{2}+0.044{w}_{3}+{0.176}{w}_{4}+{0.381}{w}_{5}+{0.188}{w}_{6}+0.611{w}_{7}+{w}_{8}+0.139{w}_{9}+{0.455}{w}_{10}
\\ &+{0.224}{w}_{11}+{0.71}{w}_{12}+{0.517}{w}_{13},\\
Eq{r}_{4}&={w}_{1}+{0.021}{w}_{2}+{0.045}{w}_{3}+{0.178}{w}_{4}+{0.382}{w}_{5}+{0.189}{w}_{6}+{0.622}{w}_{7}+0.933{w}_{8}+{0.14}{w}_{9}+{0.455}{w}_{10}
\\& +{0.092}{w}_{11}+{0.085}{w}_{12}+{0.669}{w}_{13},\\
Eq{r}_{5}&={0.56}{w}_{1}+0.268{w}_{2}+0.88{w}_{3}+0.569{w}_{4}+0.403{w}_{5}+{w}_{6}+{w}_{7}+{0.311}{w}_{8}+0.746{w}_{9}+{w}_{10}
\\
&+0.429{w}_{11}+{w}_{12}+{w}_{13}.\end{aligned}$$
8. Construct a linear programming model.
  According to Eq. (51) and the results of step (8), the linear programming model is constructed as Eq. (58):
  58 $$\begin{array}{l}\begin{array}{l}{\text{min}}\theta ={\theta }_{54}+{\theta }_{51}+{\theta }_{32}+{\theta }_{12} \\ \end{array}\\ {\text{S.T.}}\left\{\begin{array}{l}\begin{array}{l}{0.44}{w}_{1}-{0.247}{w}_{2}-{0.835}{w}_{3}-{0.391}{w}_{4}-{0.021}{w}_{5}-{0.811}{w}_{6}-{0.378}{w}_{7}+{0.622}{w}_{8}\\ -{0.606}{w}_{9}-{0.545}{w}_{10}-{0.337}{w}_{11}-{0.915}{w}_{12}-{0.331}{w}_{13}\ge -{\theta }_{54}\end{array}\\ \begin{array}{l}-{0.475}{w}_{1}+{0.732}{w}_{2}+{0.12}{w}_{3}+{0.431}{w}_{4}+{0.597}{w}_{5}-{0.008}{w}_{6}-{0.983}{w}_{7}-{0.306}{w}_{8}\\ \text{ +0.254}{w}_{9}-{0.99}{w}_{10}+{0.571}{w}_{11}+ 0 {w}_{12}-{0.954}{w}_{13}\ge -{\theta }_{51}\end{array}\\ \begin{array}{l}\begin{array}{l}-{0.849}{w}_{1}+{0.492}{w}_{2}+{0.235}{w}_{3}+{0.824}{w}_{4}-{0.01}{w}_{5}+{0.252}{w}_{6}-{0.597}{w}_{7}-{0.985}{w}_{8}\\ \text{ +0.653}{w}_{9}-{0.436}{w}_{10}+{0.329}{w}_{11}+{0.2}{9}_{12}-{0.405}{w}_{13}\ge -{\theta }_{32}\end{array}\\ \begin{array}{l}{0.066}{w}_{1}-{0.488}{w}_{2}-{0.722}{w}_{3}+ 0 {w}_{4}-{0.629}{w}_{5}-{0.552}{w}_{6}-{0.003}{w}_{7}+{0.01}{w}_{8}-{0.207}{w}_{9}\\ \text{ +0.01}{w}_{10}-{0.446}{w}_{11}+ 0 {w}_{12}+{0.067}{w}_{13}\ge -{\theta }_{12}\end{array}\\ \begin{array}{l}{w}_{1}+{w}_{2}+{w}_{3}+{w}_{4}+{w}_{5}+{w}_{6}+{w}_{7}+{w}_{8}+{w}_{9}+{w}_{10}+{w}_{11}+{w}_{12}+{w}_{13}=1\\ {w}_{1},{w}_{2},{w}_{3},{w}_{4},{w}_{5},{w}_{6},{w}_{7},{w}_{8},{w}_{9},{w}_{10},{w}_{11},{w}_{12},{w}_{13}\ge 0\\ {\theta }_{54},{\theta }_{51},{\theta }_{32},{\theta }_{12}\ge 0\end{array}\end{array}\end{array}\right.\end{array}.$$
9. Derive the attribute weights of *R*.
  By solving Eq. (58), the attribute weight $$w$$ of *R* is shown as follows:
  $$ w = \left( {\begin{array}{*{20}l} {0.0876,0.0411,{ }0.0964,{ }0.0008,{ }0.0721,{ }0.0937,0.1259,} \hfill \\ {0.0862,{ }0.0429,{ }0.1205,{ }0.0557,{ }0.0689,{ }0.1082} \hfill \\ \end{array} } \right). $$
10. Obtain the optimal alternative $${\overline{R}}$$.
  The element with the highest score in each column of the decision matrix *R* can be found using Eq. (53). All elements with the highest score are
  $$\begin{array}{ccc}{{\overline{r}}}_{01}=\left(\left[{0.55,0.65}\right],\left[{0.35,0.45}\right]\right),& {{\overline{r}}}_{02}=\left(\left[{0.80,0.90}\right],\left[{0.10,0.20}\right]\right),& {{\overline{r}}}_{03}=\left(\left[{0.78,0.88}\right],\left[{0.12,0.23}\right]\right),\\ {{\overline{r}}}_{04}=\left(\left[{0.80,0.90}\right],\left[{0.10,0.20}\right]\right),& {{\overline{r}}}_{05}=\left(\left[{0.80,0.90}\right],\left[{0.10,0.20}\right]\right), & {{\overline{r}}}_{06}=\left(\left[{0.78,0.89}\right],\left[{0.12,0.22}\right]\right),\\ {{\overline{r}}}_{07}=\left(\left[{0.77,0.87}\right],\left[{0.14,0.25}\right]\right),& {{\overline{r}}}_{08}=\left(\left[{0.80,0.90}\right],\left[{0.10,0.20}\right]\right),& {{\overline{r}}}_{09}=\left(\left[{0.52,0.62}\right],\left[{0.42,0.53}\right]\right),\\ {{\overline{r}}}_{010}=\left(\left[{0.78,0.88}\right],\left[{0.12,0.23}\right]\right),& {{\overline{r}}}_{011}=\left(\left[{0.78,0.88}\right],\left[{0.12,0.23}\right]\right),& {{\overline{r}}}_{012}=\left(\left[{0.80,0.90}\right],\left[{0.10,0.20}\right]\right),\\ {{\overline{r}}}_{013}=\left(\left[{0.75,0.87}\right],\left[{0.14,0.26}\right]\right).& & \end{array}$$
  By adding these elements to row 0 of the matrix *R*, we can get an optimal matrix $$\overline{R }={({\overline{r} }_{ij})}_{6\times 13}$$ as shown in Table 19.
11. Aggregate the attribute of $$\overline{R }$$.

**Table 19:** Decision matrix ${\overline{R}}$ of the optimal alternative

Index	$${x}_{0}$$	$${x}_{1}$$	$${x}_{2}$$	$${x}_{3}$$	$${x}_{4}$$	$${x}_{5}$$
$${C}_{1}$$	$$([{0.55,0.65}],[{0.35,0.45}])$$	$$([{0.10,0.20}],[{0.80,0.90}])$$	$$([{0.20,0.35}],[{0.65,0.8}])$$	$$([{0.55,0.65}],[{0.35,0.45}])$$	$$([{0.55,0.65}],[{0.35,0.45}])$$	$$([{0.45,0.55}],[{0.45,0.55}])$$
$${C}_{2}$$	$$([{0.80,0.90}],[{0.10,0.20}])$$	$$([{0.80,0.90}],[{0.10,0.20}])$$	$$([{0.62,0.75}],[{0.25,0.39}])$$	$$([{0.18,0.31}],[{0.72,0.84}])$$	$$([{0.18,0.31}],[{0.72,0.84}])$$	$$([{0.52,0.62}],[{0.39,0.49}])$$
$${C}_{3}$$	$$([{0.78,0.88}],[{0.12,0.23}])$$	$$([{0.78,0.88}],[{0.12,0.23}])$$	$$([{0.52,0.62}],[{0.42,0.54}])$$	$$([{0.23,0.38}],[{0.64,0.78}])$$	$$([{0.23,0.39}],[{0.63,0.78}])$$	$$([{0.74,0.85}],[{0.16,0.28}])$$
$${C}_{4}$$	$$([{0.80,0.90}],[{0.10,0.20}])$$	$$([{0.80,0.90}],[{0.10,0.20}])$$	$$([{0.80,0.90}],[{0.10,0.20}])$$	$$([{0.45,0.55}],[{0.46,0.56}])$$	$$([{0.45,0.55}],[{0.46,0.56}])$$	$$([{0.64,0.78}],[{0.22,0.37}])$$
$${C}_{5}$$	$$([{0.80,0.90}],[{0.10,0.20}])$$	$$([{0.80,0.90}],[{0.10,0.20}])$$	$$([{0.57,0.69}],[{0.34,0.47}])$$	$$([{0.57,0.69}],[{0.34,0.47}])$$	$$([{0.57,0.69}],[{0.34,0.47}])$$	$$([{0.58,0.70}],[{0.32,0.46}])$$
$${C}_{6}$$	$$([{0.78,0.89}],[{0.12,0.22}])$$	$$([{0.78,0.88}],[{0.12,0.23}])$$	$$([{0.59,0.71}],[{0.31,0.45}])$$	$$([{0.45,0.55}],[{0.46,0.56}])$$	$$([{0.45,0.55}],[{0.46,0.56}])$$	$$([{0.78,0.89}],[{0.12,0.22}])$$
$${C}_{7}$$	$$([{0.77,0.87}],[{0.14,0.25}])$$	$$([{0.13,0.25}],[{0.76,0.87}])$$	$$([{0.12,0.22}],[{0.78,0.89}])$$	$$([{0.64,0.76}],[{0.27,0.4}])$$	$$([{0.65,0.77}],[{0.26,0.40}])$$	$$([{0.77,0.87}],[{0.14,0.25}])$$
$${C}_{8}$$	$$([{0.80,0.90}],[{0.10,0.20}])$$	$$([{0.09,0.13}],[{0.84,0.92}])$$	$$([{0.15,0.28}],[{0.73,0.86}])$$	$$([{0.80,0.90}],[{0.10,0.20}])$$	$$([{0.78,0.88}],[{0.12,0.23}])$$	$$([{0.54,0.65}],[{0.38,0.50}])$$
$${C}_{9}$$	$$([{0.52,0.62}],[{0.42,0.53}])$$	$$([{0.52,0.62}],[{0.42,0.53}])$$	$$([{0.47,0.56}],[{0.45,0.55}])$$	$$([{0.10,0.17}],[{0.81,0.91}])$$	$$([{0.10,0.17}],[{0.81,0.90}])$$	$$([{0.45,0.55}],[0.4{6,0.56}])$$
$${C}_{10}$$	$$([{0.78,0.88}],[{0.12,0.23}])$$	$$([{0.10,0.20}],[{0.80,0.90}])$$	$$([{0.16,0.29}],[{0.72,0.85}])$$	$$([{0.59,0.71}],[{0.31,0.44}])$$	$$([{0.59,0.71}],[{0.31,0.44}])$$	$$([{0.78,0.88}],[{0.12,0.23}])$$
$${C}_{11}$$	$$([{0.78,0.88}],[{0.12,0.23}])$$	$$([{0.78,0.88}],[{0.12,0.23}])$$	$$([{0.62,0.76}],[{0.25,0.39}])$$	$$([{0.48,0.58}],[{0.44,0.54}])$$	$$([{0.35,0.45}],[{0.55,0.65}])$$	$$([{0.58,0.70}],[{0.32,0.46}])$$
$${C}_{12}$$	$$([{0.80,0.90}],[{0.10,0.20}])$$	$$([{0.80,0.90}],[{0.10,0.20}])$$	$$([{0.80,0.90}],[{0.10,0.20}])$$	$$([{0.69,0.83}],[{0.18,0.32}])$$	$$([{0.35,0.45}],[{0.55,0.65}])$$	$$([{0.80,0.90}],[{0.10,0.20}])$$
$${C}_{13}$$	$$([{0.75,0.87}],[{0.14,0.26}])$$	$$([{0.24,0.37}],[{0.66,0.78}])$$	$$([{0.35,0.49}],[{0.57,0.68}])$$	$$([{0.60,0.73}],[{0.30,0.45}])$$	$$([{0.66,0.77}],[{0.25,0.38}])$$	$$([{0.75,0.87}],[{0.14,0.26}])$$
  Equation (55) is used to aggregate the elements $${\overline{r} }_{ij}$$ of each row of $$\overline{R }$$. The results of each alternative of $$b{{\overline{r}}}_{i}$$ are
  $$ \begin{array}{*{20}l} {b\overline{r}_{0} = \left( {\left[ {0.76,0.87} \right],\left[ {0.14,0.26} \right]} \right)} & {b\overline{r}_{1} = \left( {\left[ {0.58,0.66} \right],\left[ {0.65,0.74} \right]} \right)} & {b\overline{r}_{2} = \left( {\left[ {0.43,0.56} \right],\left[ {0.62,0.73} \right]} \right)} \\ {b\overline{r}_{3} = \left( {\left[ {0.61,0.71} \right],\left[ {0.42,0.54} \right]} \right)} & {b\overline{r}_{4} = \left( {\left[ {0.58,0.68} \right],\left[ {0.46,0.57} \right]} \right)} & {b\overline{r}_{5} = \left( {\left[ {0.71,0.82} \right],\left[ {0.21,0.34} \right]} \right)} \\ \end{array} . $$
12. Obtain the score of alternatives.
  The score $$s{{\overline{r}}}_{i}$$ of $$b{{\overline{r}}}_{i}$$ is calculated by Eq. (56). They are
  $$\begin{aligned}
s{\overline{r}}_{0}  &= 0.6758, \quad s{\overline{r}}_{1}  = 0.0194, \quad
s{\overline{r}}_{2}  =  - 0.0872, \\ s{\overline{r}}_{3}  &= 0.2494 \quad s{\overline{r}}_{4}
= 0.1857,~s{\overline{r}}_{5}  = 0.5520  .\end{aligned}
$$
13. Calculate the utility degrees of the alternatives.
  The utility degrees of the alternatives are calculated by Eq. (57):
  $$  \begin{aligned}   e_{1}  &=
0.0288, \quad e_{2}  =  - 0.1290, \\ e_{3}  &= 0.3690, \quad e_{4}
= 0.2748, \quad e_{5}  = 0.8168   .\end{aligned}
$$
  15. Rank alternatives and select the best alternative.

The utility degree ranking of the alternatives is ${e}_{5}>{e}_{3}>{e}_{4}>{e}_{1}>{e}_{2}$. Thus, the ranking of alternatives is ${x}_{5}>{x}_{3}>{x}_{4}>{x}_{1}>{x}_{2}$, and the ${x}_{5}$ represents the best alternative, it means that the 5th software product is an ideal HFU system for purchasing. In addition, the result is consistent with the preference pairs preset by experts. It can be concluded that the proposed MAGDM is effective and objective.

### Sensitivity analysis of evaluation parameters

**Fig. 3:**
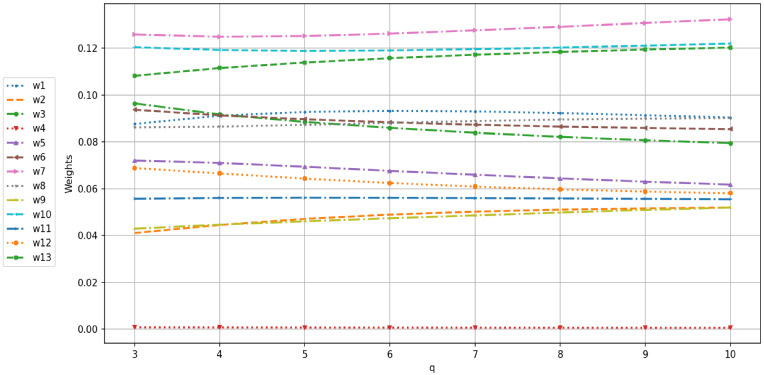
The attribute weight influence of *q* value

**Fig. 4:**
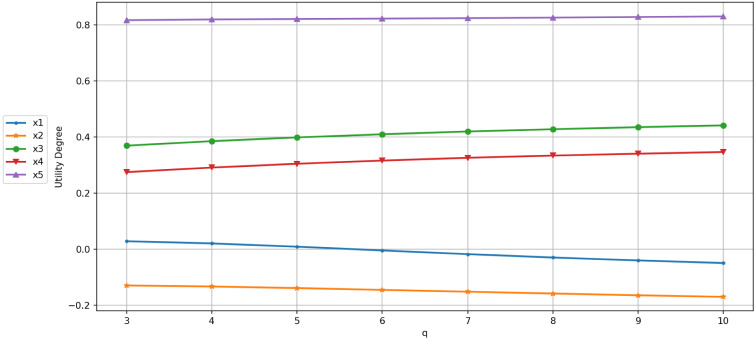
The influence of *q* value changes on decision-making methods

In order to further verify the influence of the value of *q*, we varied *q* without changing the expert decision matrix. The analysis is taken from the perspective of alternative attribute weights and utility degrees. When *q* value changes from 3 to 10, the changes of alternative attribute weights and utility degrees are shown in Figs. 3 and 4.

**Fig. 5:**
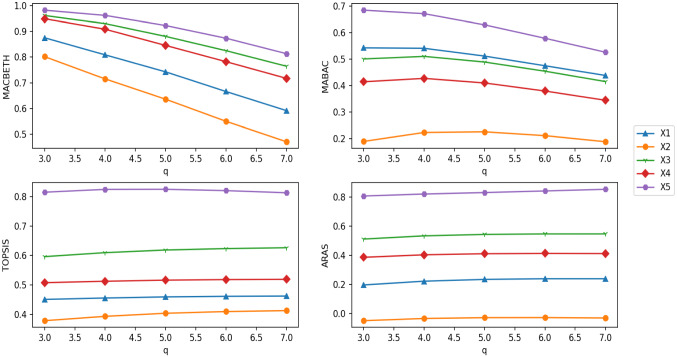
Comparative analysis of decision-making methods

**Fig. 6:**
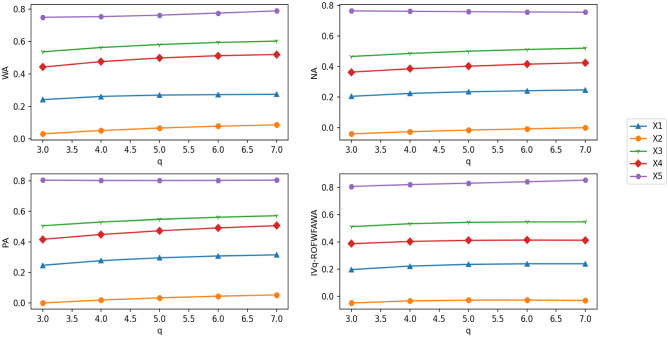
Comparison chart of different aggregation matrix operators

Figure 3 shows that with the increase of *q*, the changing trend of the attribute weights remains in the same direction. If attribute weights increase with the increase of *q*, the changing trend of the attribute weights also increases.

Figure 4 shows that when *q* changes from 3 to 10, each alternative fluctuates within a certain range and that the ranking of each alternative is basically unchanged.

### Comparative analysis

#### Comparative analysis of decision-making methods

To analyze the influence of different decision-making methods, the ARAS method is compared to MACBETH [2], MABAC [48] and TOPSIS [24]. The results are shown in Table 20.

**Table 20:** Comparative analysis of decision-making methods

	MACBETH	MABAC	TOPOSIS	ARAS (our)	Ranking
*q* = 3	${sf}_{1}=0.8755$ ${sf}_{2}=0.8019$ ${sf}_{3}=0.962$ 0 ${sf}_{4}=0.9495$ ${sf}_{5}=0.9827$	${S}_{1}=0.5425$ ${S}_{2}=0.1902$ ${{S}}_{3}=0.5006$ ${{S}}_{4}=0.4146$ ${{S}}_{5}=0.6840$	${C}_{1}=0.4509$ ${C}_{2}=0.3784$ ${C}_{3}=0.5964$ ${C}_{4}=0.5077$ ${C}_{5}=0.8150$	${U}_{1}=0.1980$ ${U}_{2}=-0.0476$ ${U}_{3}=0.5136$ ${U}_{4}=0.3880$ ${U}_{5}=0.8082$	MACBETH ${x}_{5}>{x}_{3}>{x}_{4}>{x}_{1}>{x}_{2}$
					MABAC ${x}_{5}>{x}_{1}>{x}_{3}>{x}_{4}>{x}_{2}$
					TOPSIS ${x}_{5}>{x}_{3}>{x}_{4}>{x}_{1}>{x}_{2}$
					ARAS ${x}_{5}>{x}_{3}>{x}_{4}>{x}_{1}>{x}_{2}$
*q* = 4	${sf}_{1}=0.8089$ ${sf}_{2}=0.7151$ ${sf}_{3}=0.9306$ ${sf}_{4}=0.9087$ ${sf}_{5}=0.9624$	${S}_{1}=0.5408$ ${S}_{2}=0.2239$ ${S}_{3}=0.5102$ ${S}_{4}=0.4273$ ${S}_{5}=0.6708$	${C}_{1}=0.4556$ ${C}_{2}=0.3932$ ${C}_{3}=0.6099$ ${C}_{4}=0.5126$ ${C}_{5}=0.8246$	${U}_{1}=0.2239$ ${U}_{2}=-0.0323$ ${U}_{3}=0.53$ 54 ${U}_{4}=0.4052$ ${U}_{5}=0.8218$	MACBETH ${x}_{5}>{x}_{3}>{x}_{4}>{x}_{1}>{x}_{2}$
					MABAC ${x}_{5}>{x}_{1}>{x}_{3}>{x}_{4}>{x}_{2}$
					TOPSIS ${x}_{5}>{x}_{3}>{x}_{4}>{x}_{1}>{x}_{2}$
					ARAS ${x}_{5}>{x}_{3}>{x}_{4}>{x}_{1}>{x}_{2}$
*q* = 5	${sf}_{1}=0.7426$ ${sf}_{2}=0.6359$ ${sf}_{3}=0.8809$ ${sf}_{4}=0.8457$ ${sf}_{5}=0.9232$	${S}_{1}=0.5114$ ${S}_{2}=0.2264$ ${S}_{3}=0.4889$ ${S}_{4}=0.4100$ ${S}_{5}=0.6290$	${C}_{1}=0.4594$ ${C}_{2}=0.4037$ ${C}_{3}=0.6189$ ${C}_{4}=0.5164$ ${C}_{5}=0.8250$	${U}_{1}=0.2362$ ${U}_{2}=-0.0267$ ${U}_{3}=0.5453$ ${U}_{4}=0.4127$ ${U}_{5}=0.8318$	MACBETH ${x}_{5}>{x}_{3}>{x}_{4}>{x}_{1}>{x}_{2}$
					MABAC ${x}_{5}>{x}_{1}>{x}_{3}>{x}_{4}>{x}_{2}$
					TOPSIS ${x}_{5}>{x}_{3}>{x}_{4}>{x}_{1}>{x}_{2}$
					ARAS ${x}_{5}>{x}_{3}>{x}_{4}>{x}_{1}>{x}_{2}$
*q* = 6	${sf}_{1}=0.6658$ ${sf}_{2}=0.5498$ ${sf}_{3}=0.8252$ ${sf}_{4}=0.7823$ ${sf}_{5}=0.8738$	${{S}}_{1}=0.4749$ ${S}_{2}=0.2119$ ${S}_{3}=0.4543$ ${S}_{4}=0.3796$ ${S}_{5}=0.5785$	${C}_{1}=0.4614$ ${C}_{2}=0.4095$ ${C}_{3}=0.6237$ ${C}_{4}=0.5184$ ${C}_{5}=0.8204$	${U}_{1}=0.2408$ ${U}_{2}=-0.0263$ ${U}_{3}=0.5486$ ${U}_{4}=0.4148$ ${U}_{5}=0.8428$	MACBETH ${x}_{5}>{x}_{3}>{x}_{4}>{x}_{1}>{x}_{2}$
					MABAC ${x}_{5}>{x}_{1}>{x}_{3}>{x}_{4}>{x}_{2}$
					TOPSIS ${x}_{5}>{x}_{3}>{x}_{4}>{x}_{1}>{x}_{2}$
					ARAS ${x}_{5}>{x}_{3}>{x}_{4}>{x}_{1}>{x}_{2}$
*q* = 7	${sf}_{1}=0.5914$ ${sf}_{2}=0.4706$ ${sf}_{3}=0.7646$ ${sf}_{4}=0.7173$ ${sf}_{5}=0.8136$	${S}_{1}=0.4382$ ${S}_{2}=0.1890$ ${S}_{3}=0.4152$ ${S}_{4}=0.3446$ ${S}_{5}=0.5259$	${C}_{1}=0.4622$ ${C}_{2}=0.4127$ ${C}_{3}=0.6266$ ${C}_{4}=0.5192$ ${C}_{5}=0.8131$	${U}_{1}=0.2408$ ${U}_{2}=-0.0289$ ${U}_{3}=0.5487$ ${U}_{4}=0.4138$ ${U}_{5}=0.8544$	MACBETH ${x}_{5}>{x}_{3}>{x}_{4}>{x}_{1}>{x}_{2}$
					MABAC ${x}_{5}>{x}_{1}>{x}_{3}>{x}_{4}>{x}_{2}$
					TOPSIS ${x}_{5}>{x}_{3}>{x}_{4}>{x}_{1}>{x}_{2}$
					ARAS ${x}_{5}>{x}_{3}>{x}_{4}>{x}_{1}>{x}_{2}$

It can be seen from Fig. 5 that the optimal alternative is ${x}_{5}$ obtained by the ARAS, MACBETH, MABAC and TOPSIS methods under the environment of IVq-ROFS. It can be proved that they can obtain the same optimal alternative. At the same time, it can be seen from Fig. 5 that the alternative ranking results obtained by ARAS, MACBETH and TOPSIS are completely consistent: ${x}_{5}>{x}_{3}>{x}_{4}>{x}_{1}>{x}_{2}$, which proves that the ARAS is effective and feasible. The ranking result of MABAC is ${x}_{5}>{x}_{1}>{x}_{3}>{x}_{4}>{x}_{2}$, implying that ${x}_{1}$ is superior to alternative ${x}_{3}$ and alternative ${x}_{4}$. However, since the alternatives ${x}_{3}$ and ${x}_{4}$ are preferable to ${x}_{1}$ for ARAS, MACBETH and TOPSIS, ARAS is more stable than MABAC. At the same time, it can be seen from Fig. 5 that the deviation of alternatives score obtained by ARAS is easier to distinguish than MACBETH, MABAC and TOPSIS, allowing decision-makers to easily obtain the ranking results.

#### Comparative analysis of aggregate operators

To analyze the influence of different aggregating operators, the IVq-ROFWFAWA (WFAWA) operator is compared with the WA [67], NA [18] and PA [69] aggregate operators in the decision-making process under an IVq-ROFS environment. The comparison results are presented in Table 21 and Fig. 6.

**Table 21:** Comparative analysis of aggregate operators

	WA	NA	PA	WFAWA (our)	Ranking
*q* = 3	${U}_{1}=0.2415$ ${U}_{2}=0.0307$ ${U}_{3}=0.5370$ ${U}_{4}=0.4424$ ${U}_{5}=0.7494$	${U}_{1}=0.2039$ ${U}_{2}=-0.0420$ ${U}_{3}=0.4659$ ${U}_{4}=0.3630$ ${U}_{5}=0.7647$	${U}_{1}=0.2476$ ${U}_{2}=0.0007$ ${U}_{3}=0.5064$ ${U}_{4}=0.4175$ ${U}_{5}=0.8035$	${U}_{1}=0.1980$ ${U}_{2}=-0.0476$ ${U}_{3}=0.5136$ ${U}_{4}=0.3880$ ${U}_{5}=0.8082$	WA ${x}_{5}>{x}_{3}>{x}_{4}>{x}_{1}>{x}_{2}$
					NA ${x}_{5}>{x}_{3}>{x}_{4}>{x}_{1}>{x}_{2}$
					PA ${x}_{5}>{x}_{3}>{x}_{4}>{x}_{1}>{x}_{2}$
					WFAWA ${x}_{5}>{x}_{3}>{x}_{4}>{x}_{1}>{x}_{2}$
*q* = 4	${U}_{1}=0.2611$ ${U}_{2}=0.0513$ ${U}_{3}=0.5636$ ${U}_{4}=0.4763$ ${U}_{5}=0.7534$	${U}_{1}=0.2229$ ${U}_{2}=-0.0277$ ${U}_{3}=0.4858$ ${U}_{4}=0.3850$ ${U}_{5}=0.7618$	${U}_{1}=0.2779$ ${U}_{2}=0.0200$ ${U}_{3}=0.5307$ ${U}_{4}=0.4491$ ${U}_{5}=0.8017$	${U}_{1}=0.2239$ ${U}_{2}=-0.0323$ ${U}_{3}=0.5354$ ${U}_{4}=0.4052$ ${U}_{5}=0.8218$	WA ${x}_{5}>{x}_{3}>{x}_{4}>{x}_{1}>{x}_{2}$
					NA ${x}_{5}>{x}_{3}>{x}_{4}>{x}_{1}>{x}_{2}$
					WA ${x}_{5}>{x}_{3}>{x}_{4}>{x}_{1}>{x}_{2}$
					WFAWA ${x}_{5}>{x}_{3}>{x}_{4}>{x}_{1}>{x}_{2}$
*q* = 5	${U}_{1}=0.2694$ ${U}_{2}=0.0668$ ${U}_{3}=0.5820$ ${U}_{4}=0.4988$ ${U}_{5}=0.7624$	${U}_{1}=0.2339$ ${U}_{2}=-0.0171$ ${U}_{3}=0.5004$ ${U}_{4}=0.4020$ ${U}_{5}=0.7593$	${U}_{1}=0.2964$ ${U}_{2}=0.0343$ ${U}_{3}=0.5481$ ${U}_{4}=0.4733$ ${U}_{5}=0.8013$	${U}_{1}=0.2362$ ${U}_{2}=-0.0267$ ${U}_{3}=0.5453$ ${U}_{4}=0.4127$ ${U}_{5}=0.8318$	WA ${x}_{5}>{x}_{3}>{x}_{4}>{x}_{1}>{x}_{2}$
					NA ${x}_{5}>{x}_{3}>{x}_{4}>{x}_{1}>{x}_{2}$
					PA ${x}_{5}>{x}_{3}>{x}_{4}>{x}_{1}>{x}_{2}$
					WFAWA ${x}_{5}>{x}_{3}>{x}_{4}>{x}_{1}>{x}_{2}$
*q* = 6	${U}_{1}=0.2722$ ${U}_{2}=0.0780$ ${U}_{3}=0.5948$ ${U}_{4}=0.5129$ ${U}_{5}=0.7754$	${U}_{1}=0.2405$ ${U}_{2}=-0.0088$ ${U}_{3}=0.5119$ ${U}_{4}=0.4153$ ${U}_{5}=0.7573$	${U}_{1}=0.3083$ ${U}_{2}=0.0452$ ${U}_{3}=0.5608$ ${U}_{4}=0.4921$ ${U}_{5}=0.8022$	${U}_{1}=0.2408$ ${U}_{2}=-0.0263$ ${U}_{3}=0.5486$ ${U}_{4}=0.4148$ ${U}_{5}=0.8428$	WA ${x}_{5}>{x}_{3}>{x}_{4}>{x}_{1}>{x}_{2}$
					NA ${x}_{5}>{x}_{3}>{x}_{4}>{x}_{1}>{x}_{2}$
					PA ${x}_{5}>{x}_{3}>{x}_{4}>{x}_{1}>{x}_{2}$
					WFAWA ${x}_{5}>{x}_{3}>{x}_{4}>{x}_{1}>{x}_{2}$
*q* = 7	${U}_{1}=0.2743$ ${U}_{2}=0.0864$ ${U}_{3}=0.6032$ ${U}_{4}=0.5203$ ${U}_{5}=0.7890$	${U}_{1}=0.2461$ ${U}_{2}=-0.0012$ ${U}_{3}=0.5204$ ${U}_{4}=0.4249$ ${U}_{5}=0.7562$	${U}_{1}=0.3158$ ${U}_{2}=0.0536$ ${U}_{3}=0.5710$ ${U}_{4}=0.5071$ ${U}_{5}=0.8038$	${U}_{1}=0.2408$ ${U}_{2}=-0.0289$ ${U}_{3}=0.5487$ ${U}_{4}=0.4138$ ${U}_{5}=0.8544$	WA ${x}_{5}>{x}_{3}>{x}_{4}>{x}_{1}>{x}_{2}$
					NA ${x}_{5}>{x}_{3}>{x}_{4}>{x}_{1}>{x}_{2}$
					PA ${x}_{5}>{x}_{3}>{x}_{4}>{x}_{1}>{x}_{2}$
					WFAWA ${x}_{5}>{x}_{3}>{x}_{4}>{x}_{1}>{x}_{2}$

As can be seen from Fig. 6, for IVq-ROFWFAWA, WA, NA and PA aggregate operators, the results of the ranking of each alternative are generally consistent across all four operators. At the same time, the IVq-ROFWFAWA operator is still the most concise and efficient and outperforms the other operators when choosing the best alternative.

Based on the implementation and comparative analysis of the case in this paper, the optimal HFU system can be selected from the five HFU systems by our developed GDM method. In addition, our research work has advantages. (1) Compared with q-ROFSs, the proposed GDM method can expand the freedom of DMs under IVq-ROFS. (2) The new score function can better distinguish IVq-ROFNs which further improves the processing ability of the proposed GDM method. (3) Our developed GDM method does not need attribute weights and expert weights, which can save evaluating time and reduce decision-making costs. (4) The proposed method for deriving expert weights makes each alternative have its own expert weights, which can more objectively reflect the expert’s real situation, and which makes the proposed GDM method more suitable for evaluating a small number of alternatives in a dynamic environment. (5) The neutral IVq-ROFWFAWA operator can improve the information carry capacity of the developed GDM method, which can more accurately preserve the attitude characteristics of DMs. Therefore, our proposed GDM method can be more conveniently, objectively, and cost-effectively used for evaluating a small number of alternatives.

## Conclusion

To evaluate the differences among HFU management systems, this paper develops a MAGDM method that integrates an IVq-ROFWFAWA operator, the LINMAP method and the ARAS method under IVq-ROFNs. We designed our indices according to the features of medical software that are measured in quality reviews and evaluations. Then, we propose a novel score function that overcomes the deficiency of existing score functions for measuring IVq-ROFNs, and extend WFA operator to IVq-ROFWFAWA operator under IVq-ROFSs for aggregating information neutrally. Afterward, the attribute weights are solved through a linear programming model constructed by LINMAP, and expert weights of different alternatives are obtained based on the similarity of IVq-ROFNs. Finally, the integrated GDM method is developed to evaluate the quality of the HFU system under IVq-ROFSs. The results of the evaluation are consistent with the results provided by the experts in advance. The sensitivity analysis and comparative analysis further verify the effectiveness and feasibility of the proposed MAGDM method.

Nevertheless, our developed method is only applicable to the case of a small number of experts and a small number of alternatives, with the rapid growth of complexity and uncertainty of the system, it is necessary to combine big data and artificial intelligence to deal with complex problems in the decision-making process. For future research, we will concentrate on the study of big data decision problems. On the other hand, experts often give preference information between alternatives and provide a judgment matrix according to their own experience and knowledge. Therefore, decision-making methods based on preference relations will also be our research focus.

## Data Availability

All data used to support the findings of the study are included within the article.
